# Disassembly Mechanisms and Energetics of Polymetallic Rings and Rotaxanes

**DOI:** 10.1021/jacs.2c07522

**Published:** 2022-12-02

**Authors:** Niklas Geue, Tom S. Bennett, Alexandra-Ana-Maria Arama, Lennart A. I. Ramakers, George F. S. Whitehead, Grigore A. Timco, P. B. Armentrout, Eric J. L. McInnes, Neil A. Burton, Richard E. P. Winpenny, Perdita E. Barran

**Affiliations:** † Michael Barber Centre for Collaborative Mass Spectrometry, Department of Chemistry, Manchester Institute of Biotechnology, The University of Manchester, 131 Princess Street, ManchesterM1 7DN, U.K.; ‡ Department of Chemistry, The University of Manchester, Oxford Road, ManchesterM13 9PL, U.K.; § Department of Chemistry, University of Utah, Salt Lake City, Utah84112, United States

## Abstract

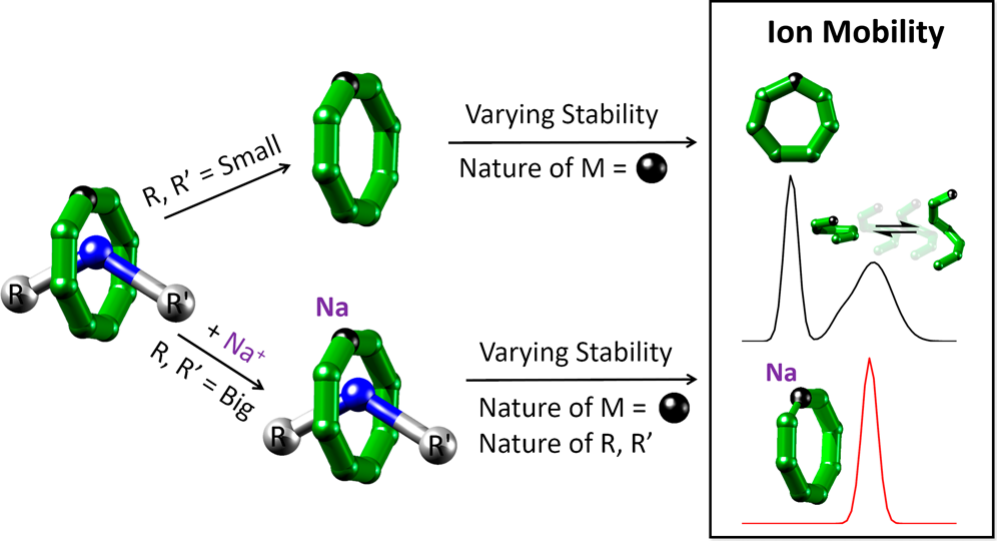

Understanding the fundamental reactivity of polymetallic complexes is challenging due to the complexity of their structures with many possible bond breaking and forming processes. Here, we apply ion mobility mass spectrometry coupled with density functional theory to investigate the disassembly mechanisms and energetics of a family of heterometallic rings and rotaxanes with the general formula [NH_2_RR’][Cr_7_MF_8_(O_2_C^*t*^Bu)_16_] with M = Mn^II^, Fe^II^, Co^II^, Ni^II^, Cu^II^, Zn^II^, Cd^II^. Our results show that their stability can be tuned both by altering the d-metal composition in the macrocycle and by the end groups of the secondary ammonium cation [NH_2_RR’]^+^. Ion mobility probes the conformational landscape of the disassembly process from intact complex to structurally distinct isobaric fragments, providing unique insights to how a given divalent metal tunes the structural dynamics.

## Introduction

Ion mobility mass spectrometry (IM-MS) allows both the mass and structure of isolated ions to be measured in the same experiment.^1−4^ Ion mobility measurements for any given analyte can be converted to the structural parameter collision cross sections (CCS), which can be compared to those predicted from theoretical candidate geometries. IM-MS has been widely applied to examine the dynamics of the disassembly of protein complexes^5−8^ but far less often to determine the relationship between conformation and stability of synthetic macromolecular complexes. A number of studies have demonstrated the use of IM-MS for the characterization of polymetallic compounds,^9−60^ but only a handful have used it to explore disassembly processes. For example, Wesdemiotis and co-workers showed the collisional activation of a terpyridine-based hexameric Cd-complex and subsequently used ion mobility to separate the macrocyclic precursor ion from linear isomers.^14^ In another study, Mallis *et al.* investigated pyridine-based Pt-rhomboids and demonstrated, among other things, different gas phase stabilities of isobaric complexes with activated IM-MS.^15^ More recently, Baksi *et al.* used IM-MS to visualize the energetically driven reaction between coin metal thiolate clusters, in which silver atoms were incrementally exchanged for gold centers.^16^ Although these are interesting examples, a systematic application of IM-MS to investigate the reactivity or disassembly of polymetallic complexes as a function of structure and metals present has not yet been reported.

We hypothesized that IM-MS would be an ideal method to investigate these systems and that it could be used to rationalize the stability of isostructural complexes as a function of the metal present along with conformation-specific information. An attractive chemical problem to answer with this approach is the conformational rigidity of rotaxanes with respect to their threads (herein used as a synonym for “axles”). To address these challenges, we use IM-MS supported by density functional theory (DFT) to characterize the heterometallic rings [Cr_7_MF_8_(O_2_C^*t*^Bu)_16_]^−^ (“[**Ring_M_**]^−^”, M = Mn^II^, Fe^II^, Co^II^, Ni^II^, Cu^II^, Zn^II^, Cd^II^) alone as well as in hybrid organic–inorganic [2]-rotaxane families of the formula [NH_2_RR’][**Ring_M_**] (“**Ph_M_**” and “**Am_M_**”), where R and R’ are terminated by bulky phenyl or *tert*-butyl groups such that the secondary ammonium cation threads through [**Ring_M_**]^−^ (**Ph_M_**: thread “**TPh^+^**” (“Phenyl”) = [NH_2_(CH_2_C_6_H_5_)(CH_2_CH_2_C_6_H_5_)]^+^; **Am_M_**: thread “**TAm^+^**” (“Amide”) = [NH_2_(C_6_H_12_NHC(O)^*t*^Bu)_2_]^+^). The studied compounds and their building blocks are illustrated in Table 1.

**Table 1:**
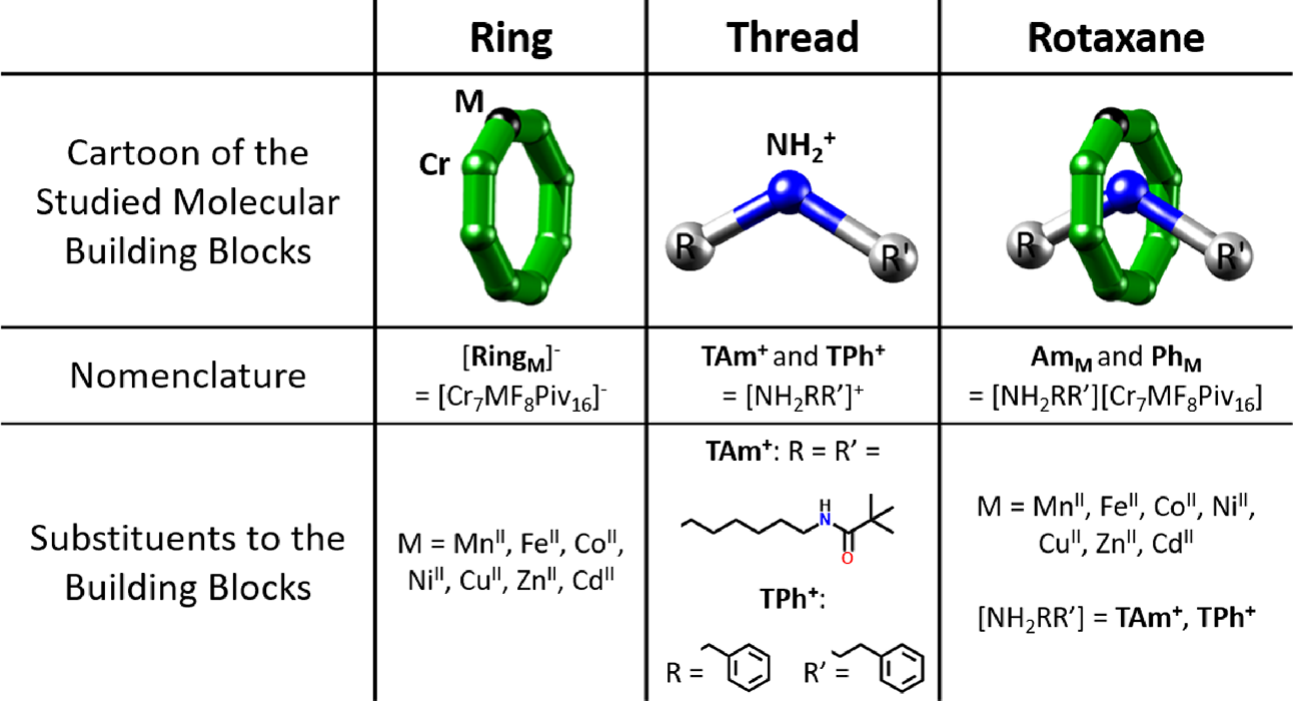
Overview of the Studied Polymetallic Systems (Rings, Threads, and Rotaxanes), Including their Chemical Formulas and the Substituents to the Building Blocks

In our analysis, we consider if the Irving–Williams series and other arguments, often developed for monometallic complexes and rationalized by crystal field theory, are transferable to polymetallic complexes. We show that IM-MS can qualitatively and quantitatively investigate the disassembly, conformational dynamics, and stabilities of these compounds. The specific systems studied have been proposed as qubits for quantum information processing,^17^ and the ability to link the rings into multiple qubit arrays^18^ is a key advantage that this approach has over more conventional approaches. Understanding the stability of the compounds is important in order to make increasingly complex molecules for specific quantum applications, e.g., synthesis of a five-spin supramolecule that could be used to simulate decoherence in Bell states.^19^ As supramolecular chemistry using polymetallic units as building blocks develops,^21−63^ the understanding of ligand metathesis achieved here should be important in designing future synthesis routes.

## Results

### Heterometallic Rings [Cr_7_MF_8_(O_2_C^*t*^Bu)_16_]^−^ = “[Ring_M_]^−^”

**Figure 1:**
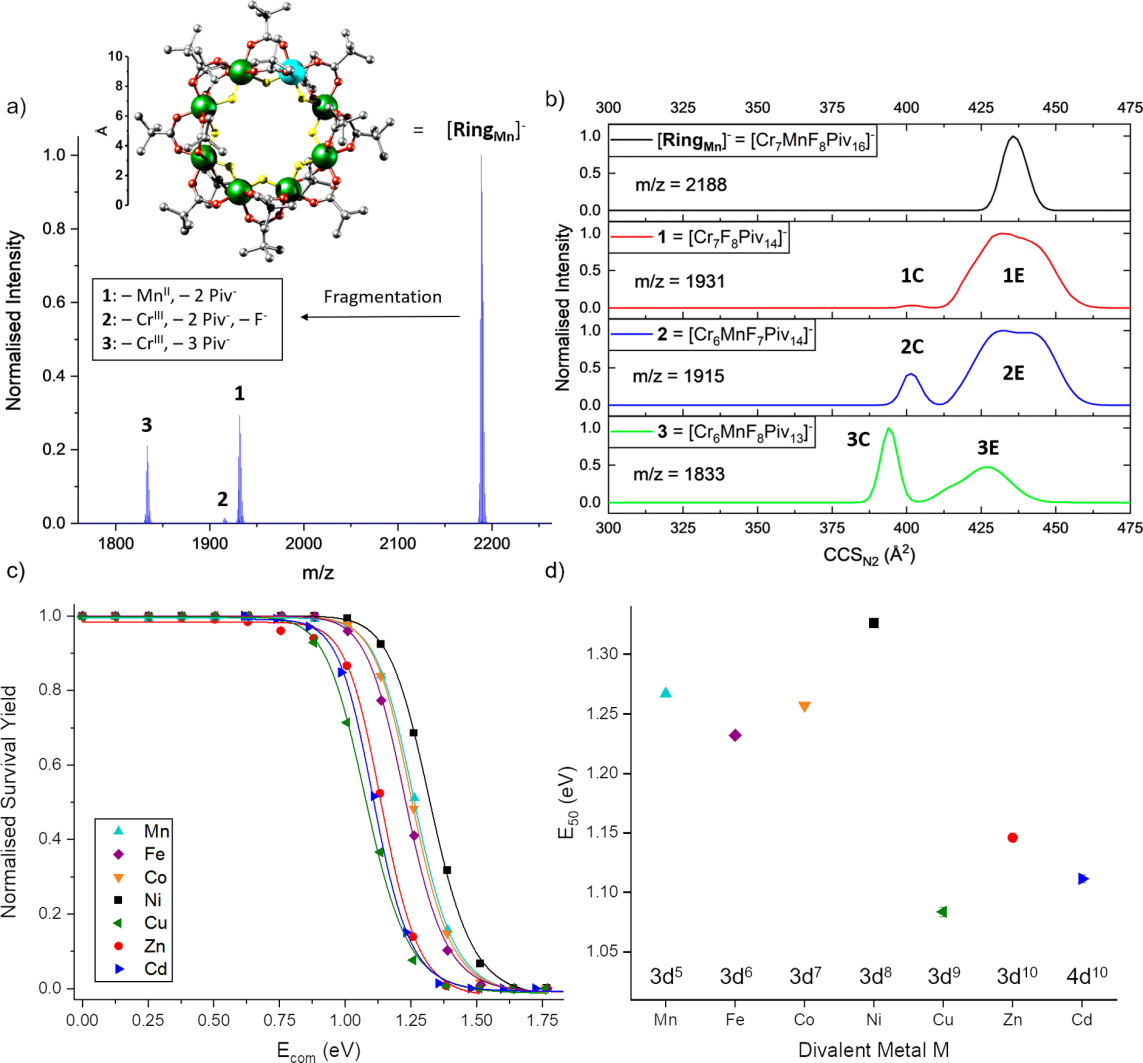
(a) MS^2^ data from [NH_2_(CH_3_)_2_][**Ring_Mn_**] (10 μM in 500 μM NaI and 4:1 toluene/methanol) at *E*_lab_ = 110 eV, following selection of [**Ring_Mn_**]^−^ (*m*/*z* = 2188). Inset: structure of [**Ring_Mn_**]^−^ (Cr: green, Mn: cyan, F: yellow, O: red, C: gray) with an Å ruler based on crystal structure coordinates. Hydrogen atoms in the *tert*-butyl groups were omitted for clarity. (b) CCS_N2_ Distributions of [**Ring_Mn_**]^−^ and selected fragments (**1**–**3**) at E_lab_ = 110 eV. (c) Normalized survival yield vs *E*_com_ for [**Ring_M_**]^−^ fitted to a sigmoidal Hill function (M = Mn: cyan, Fe: purple, Co: orange, Ni: black, Cu: green, Zn: red, Cd: blue). (d) *E*_50_ values for the dissociation of the anionic rings [**Ring_M_**]^−^ with respect to M (Table S1). Error bars are shown and in most cases are smaller than the symbol size.

The heterometallic rings [**Ring_M_**]^−^ studied consist of seven chromium(III) ions and one divalent metal center, arranged in a regular octagon with each edge bridged by one fluoride inside the ring and two pivalate ligands (O_2_C^*t*^Bu = Piv) outside the ring (Figure 1a, inset for M = Mn^II^).^23^ On each edge, there is one axial and one equatorial pivalate, with respect to the Cr_7_M plane, with the axial position alternating “up” and “down” around the ring. Following optimization of the ionization source and solvent conditions, mass spectra of [NH_2_RR’][**Ring_M_**] (R = R’ = CH_3_ for M = Mn^II^, Co^II^, Ni^II^, Cu^II^, Zn^II^, Cd^II^ and R = R’ = C_2_H_5_ for M = Fe^II^) were recorded in positive and negative modes. For all species, intact rings were observed as anions (Figure S1a for M = Mn^II^, Supplementary Data Set for other M). The assignments of all [**Ring_M_**]^−^ species, and all other ions discussed in this study, were informed by accurate masses and isotopic distributions, which were typically in high accordance with prediction (Figure S1b for [**Ring_Mn_**]^−^).

#### Disassembly and Stability Trend of [**Ring_M_**]^−^

To understand how altering the d-metal composition affects the ring energetics, we isolated each ring and ramped the energy at which it is activated by collisions while recording the arrival time distribution (ATD) of the precursor and product ions. We extracted the CCS as a function of the collision energy for all ions. In the tandem mass spectra (MS^2^), common product ions were observed, indicating similar fragmentation pathways. For example, the precursor ion [**Ring_Mn_**]^−^ at *m*/*z* = 2188 follows dissociation channels that all involve the loss of a metal center along with ligands (Figure 1a), suggesting substantial perturbation of the ring geometry. We identified the products and reaction pathways including the loss of the manganese and two pivalate ligands (reaction to [Cr_7_F_8_Piv_14_]^−^ = **1**), the loss of one chromium center, two pivalates and one fluoride (reaction to [Cr_6_MnF_7_Piv_14_]^−^ = **2**), or one chromium and three pivalate ligands (reaction to [Cr_6_MnF_8_Piv_13_]^−^ = **3**, Figure 1a). Ion mobility allows us to consider the conformations of the precursor and product ions. While [**Ring_Mn_**]^−^ presents a discrete unimodal conformer centered at CCS_N2_ = 438 Å^2^ at this collision energy, remarkably for all fragmentation channels, the product ions are structurally diverse, with narrow, unimodal conformers at lower CCS_N2_ (compact, “**C**”) as well as wide, multimodal distributions with higher CCS_N2_ (extended, **“E**”, Figure 1b). The nature of these disassembled products is investigated further below.

For all [**Ring_M_**]^−^ (M = Mn^II^, Fe^II^, Co^II^, Ni^II^, Cu^II^, Zn^II^, Cd^II^), the fragmentation behavior is similar and results in **1** as the main product. This preference for **1** over the structures similar to **2** and **3** (different M) is more pronounced for Ni^II^, Zn^II^, Cd^II^, and particularly Cu^II^ (Supplementary Data Set). The energy needed to break the [**Ring_M_**]^−^ structure, predominantly yielding **1**, also varies (Figure 1c) and was quantified with *E*_50_ values (Table S1, Figure 1d) obtained from the corresponding survival yield plots (Figure 1c). The results show that [**Ring_Ni_**]^−^ is the most stable ion and exhibits a 22% higher *E*_50_ value than the least stable species [**Ring_Cu_**]^−^.

#### Combining Ion Mobility and DFT for [**Ring_M_**]^−^ and Their Fragments

The CCS_N2_ values for each [**Ring_M_**]^−^ were similar, with [**Ring_Cd_**]^−^ and [**Ring_Mn_**]^−^ being slightly larger than the other species (Table S2). DFT optimized structures were generated for all [**Ring_M_**]^−^ (Figure S2 for M = Mn^II^), from which we obtained CCS_N2_ values using the trajectory method of IMoS.^24^ These were found to be ∼8% larger than experiment (Table S2). To investigate this slight discrepancy, two hypothetical ring conformers (Figures S3 and S4) and two open isomers (Figures S5–S7) were generated for [**Ring_Mn_**]^−^ and their theoretical CCS_N2_ values enumerated (Table S3). The two conformers yield slightly smaller but similar values to that of the DFT optimized [**Ring_Mn_**]^−^ structure, whereas the open isomers gave even higher values. Therefore, the candidate structures unlikely account for the observed differences between the experimental and predicted CCS_N2_ values of [**Ring_M_**]^−^. We attribute this systematic difference to the lack of refinement of the trajectory method for metallosupramolecular systems (Figures S8 and S9).

As discussed above, changes in arrival time distribution of [**Ring_Mn_**]^−^ were recorded as a function of incremental increases in collision energy, showing the onset of ring dissociation and the corresponding appearance of fragment ions (Figure S10). Although there is little evidence of any substantial change in the conformation of the ring prior to dissociation, at low energies, [**Ring_Mn_**]^−^ contracts slightly before expanding again. The product ions present distinct conformational families **C** and **E** (Figure 1b). The relative population of these distributions differ significantly between the fragments but also with respect to the divalent metal M as observed from measurements on other [**Ring_M_**]^−^ precursors (Supplementary Data Set). Surprisingly, the relative populations of **C** and **E** in the homometallic Cr_7_-fragment **1** allow us to distinguish which divalent metal M was present in the precursor [**Ring_M_**]^−^. Significant amounts of **1C** are observed for Cu^II^, Zn^II^, and Cd^II^ (Figure S11 for M = Cu^II^), whereas almost no **1C** is seen for Mn^II^, Fe^II^, Co^II^, and Ni^II^ (Figures S12 and S13 for M = Mn^II^, Ni^II^). For the less dominant product ions **2** and **3**, the observed differences between M are more subtle.

To rationalize this behavior, additional experiments and calculations were carried out for fragment **3** with M = Mn^II^. Each distribution was selected, reinjected in the drift ring, and again, ion mobility separated in one to three passes (IMS^2^). For **3C**, no further change is seen to the ATD, even after three passes, suggesting that this conformer is highly stable and does not interconvert (Figure S14). For the extended distribution **3E**, multiple passes broaden the ATD, but no further resolution is achieved, indicating that this feature contains interconverting species that are conformationally dynamic on the experimental timescale (∼250 ms, Figure S15). We also examined how collision energy influences the population of these features in the activated IM-MS spectra of [**Ring_Mn_**]^−^. For all fragments **1**–**3**, the **C** distributions form and subsequently dissociate at slightly lower *E*_lab_ than the extended distributions **E** (Figure S16). DFT calculations were performed to discern the structures of the **3** conformers (Table S4), including a seven-membered Cr_6_Mn-ring as a candidate for **3C** (Figure S17) and different opened helical forms as candidates for **3E** (Figures S18–S22). A comparison between their predicted CCS_N2_ values with those found experimentally is instructive in understanding the disassembly processes for [**Ring_M_**]^−^ (see below).

### [2]-Rotaxane Families [NH_2_RR’][Ring_M_] = “Ph_M_ and Am_M_”

**Figure 2:**
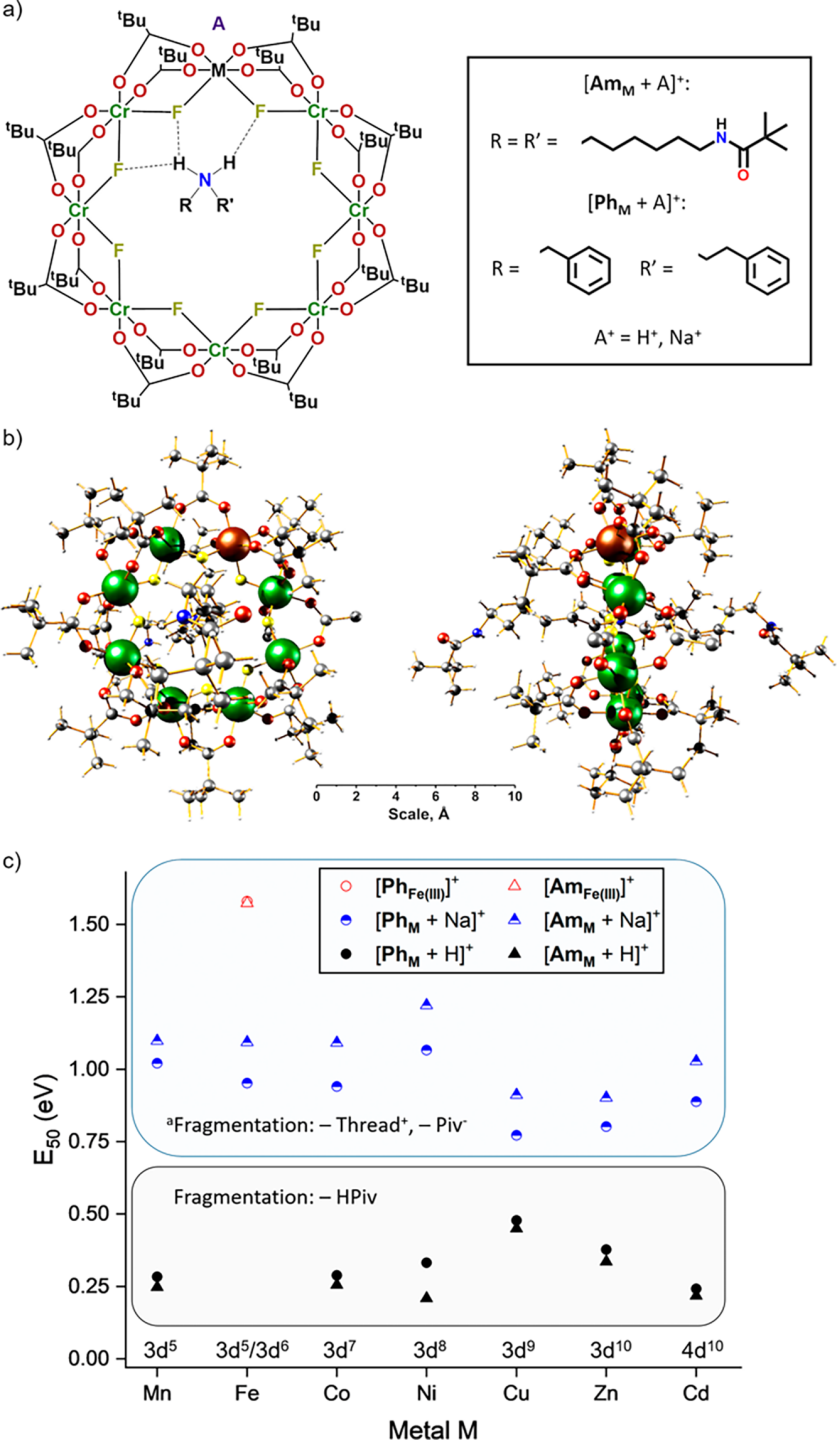
(a) Schematic of [**Am_M_** + A]^+^ and [**Ph_M_** + A]^+^. The thread is found at the ring center with the two protons on the ammonium nitrogen exhibiting hydrogen bonds to the fluorides. Hydrogen bonding, shown in the figure (dashed lines), partially localizes the divalent metal site due to the greater electron density on the fluorides bound to M^II^. The location of the charge carrier A^+^ likely depends on both A^+^ and the thread, which is further discussed in another report.^28^ (b) Single crystal X-ray structure of **Am_Ni_**(29) in front (left) and side views (right) including dimensions in Å (Ni: brown, Cr: dark green, F: yellow, O: red, N: blue, C: gray, H: white). Solvent molecules in the asymmetric unit have been removed for clarity. The Ni sites in the [**Ring_Ni_**]^−^ unit are disordered over multiple positions. (c) *E*_50_ values for the fragmentation of the rotaxane cations [**Ph_M_** + A]^+^ and [**Am_M_** + A]^+^ (A^+^ = H^+^, Na^+^) with respect to M, as well as the *E*_50_ values of the oxidized Fe^III^ species [**Ph_Fe(III)_**]^+^ and [**Am_Fe(III)_**]^+^. An overlap in the mass spectra with these species prevented further investigation of the protonated ions [**Ph_Fe_** + H]^+^ and [**Am_Fe_** + H]^+^. ^a^For the ion [**Am_Cu_** + Na]^+^, the main fragmentation channel is −Cu^II^, −2 Piv^–^ (Figure S29). Error bars are omitted for clarity but in all cases are smaller than the symbol size (Table S1). The electron configuration of Fe depends on the oxidation state (Fe^II^: 3d^6^, Fe^III^: 3d^5^).

The same workflows were applied to the rotaxane families **Ph_M_** and **Am_M_**, where the secondary ammonium cation [NH_2_RR’]^+^ (**Ph_M_**: R = CH_2_Ph, R’ = CH_2_CH_2_Ph (“**TPh**^+^”) and **Am_M_:** R = R’ = C_6_H_12_NHC(O)^*t*^Bu (“**TAm**^+^”)), referred to as the thread, was postulated to not easily dissociate from the heterometallic rings [**Ring_M_**]^−^ due to the large R, R’ groups (Figure 2a,b).^23^

#### Disassembly and Stability Trends of the Rotaxane Ions [**Ph_M_** + A]^+^ and [**Am_M_** + A]^+^ (A^+^ = H^+^, Na^+^)

**Ph_M_** and **Am_M_** were transferred to the gas phase from solutions of NaI and yielded protonated cations and sodiated forms [**Ph_M_** + A]^+^ and [**Am_M_** + A]^+^, among others (A^+^ = H^+^, Na^+^; Figure S23 for M = Co^II^, Supplementary Data Set for other M). Considerable differences are observed between their fragmentation behavior, with the sodiated forms being significantly more stable than the protonated species (Figure 2c and Figures S24–S29).

We performed a quantitative investigation of the rotaxane fragmentation and determined the *E*_50_ values for all ions [**Ph_M_** + A]^+^ and [**Am_M_** + A]^+^ (A^+^ = H^+^, Na^+^) with M = Mn^II^, Fe^II^, Co^II^, Ni^II^, Cu^II^, Zn^II^, Cd^II^ (Table S1 and Figure 2c), as well as for [**Ph_Fe(III)_**]^+^ and [**Am_Fe(III)_**]^+^, which showed the same disassembly pathway (Figure S30). The results demonstrate that three factors influence the stability of the rotaxane ions [**Ph_M_** + A]^+^ and [**Am_M_** + A]^+^: first, the metal M in the heterometallic ring and its oxidation state, second, the thread and its end group (**TPh**^+^ vs **TAm**^+^), and finally, the charge carrying species A^+^ (A^+^ = H^+^, Na^+^), where we observed a significant difference between protonated species and sodiated forms. The impact of these and other charge carriers on the disassembly, stability, and conformations of these complexes is the subject of another report.^28^

#### Ion Mobility Investigations of the Rotaxane Ions [**Ph_M_** + Na]^+^, [**Am_M_** + Na]^+^ and Their Fragments

**Figure 3:**
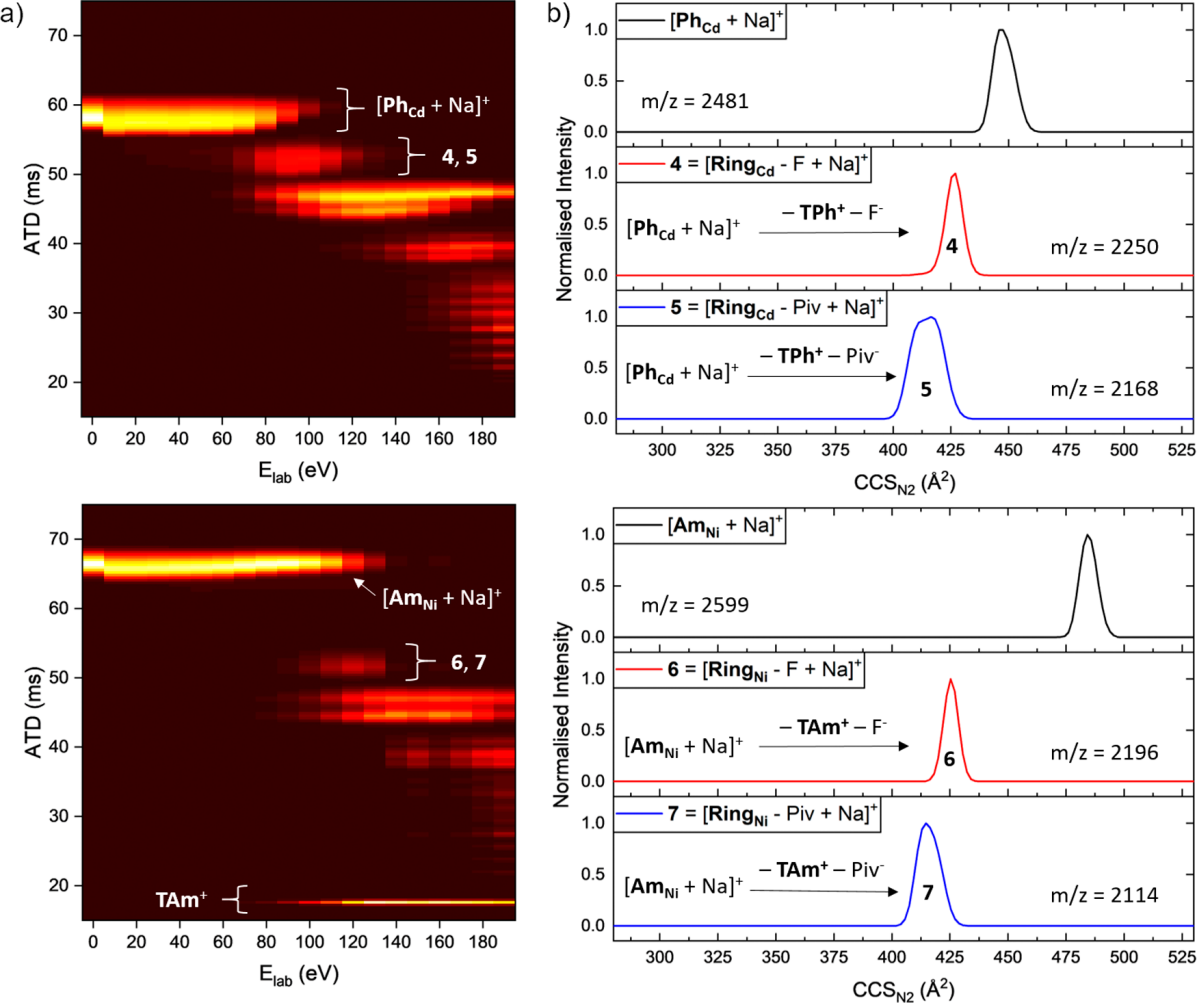
(a) Collision energy vs arrival time distribution heat map for [**Ph_Cd_** + Na]^+^ (*m*/*z* = 2481, top) and [**Am_Ni_** + Na]^+^ (*m*/*z* = 2599, bottom) as well as the corresponding fragments. 10 μM **Ph_Cd_** and **Am_Ni_** were used in 500 μM NaI and 4:1 toluene/methanol (**Ph_Cd_**) or 7:3 methanol/DCM (**Am_Ni_**). The intact thread cation **TAm**^+^ was observed, whereas no **TPh**^+^ ions could be detected (Figure S31). (b) CCS_N2_ distributions of [**Ph_Cd_** + Na]^+^ and its fragments (**4**, **5**) at *E*_lab_ = 90 eV (top) as well as [**Am_Ni_** + Na]^+^ and its fragments (**6**, **7**) at *E*_lab_ = 120 eV (bottom).

We use the same activated ion-mobility workflow as for [**Ring_M_**]^−^ to consider by what route the thread detaches from the ring in the sodiated forms. Examples of [**Ph_Cd_** + Na]^+^ and [**Am_Ni_** + Na]^+^ are presented (Figure 3), although other species were also examined and qualitatively show the same behavior (Supplementary Data Set).

The heat maps in Figure 3a illustrate the collision induced fragmentation of [**Ph_Cd_** + Na]^+^ (top) and [**Am_Ni_** + Na]^+^ (bottom) *via* changes in arrival time distribution. The dissociation of the rotaxane species occurs at different energies, with [**Am_Ni_** + Na]^+^ being significantly more stable than [**Ph_Cd_** + Na]^+^ (Figure 2c). Both precursor ions show similar behavior to [**Ring_Mn_**]^−^ (Figure S10a) with no major structural change.

As discussed before, the fragmentation channels are similar for both ions and involve the loss of the thread and one anionic ligand (F^−^: **4**, **6** and Piv^−^: **5**, **7**). The ATD of the precursor ions [**Ph_Cd_** + Na]^+^ and [**Am_Ni_** + Na]^+^ as well as their direct fragments **4**–**7** were converted to CCS_N2_ (Figure 3b), showing that [**Am_Ni_** + Na]^+^ is 9% larger than [**Ph_Cd_** + Na]^+^ due to the size of the thread end groups. Fragments **4–7** have very close CCS_N2_ values, with **5** and **7** being only slightly smaller than **4** and **6**. Fragments **4**, **6**, and **7** are unimodal, whereas **5** indicates two closely related conformers; however, none exhibit the conformational diversity shown for the fragments of [**Ring_Mn_**]^−^ (Figure 1b). The small CCS_N2_ differences between the two fragmentation channels occur because of the number of bulky pivalate ligands present (**4**, **6**: 16 Piv^−^; **5**, **7**: 15 Piv^−^), whereas the asymmetric peak shapes of **5** and **7** are possibly attributable to pivalate loss from different coordination sites, either in or perpendicular to the metal plane.

## Discussion

### Disassembly and Stability Trends

#### Heterometallic Rings [**Ring_M_**]^−^

**Figure 4:**
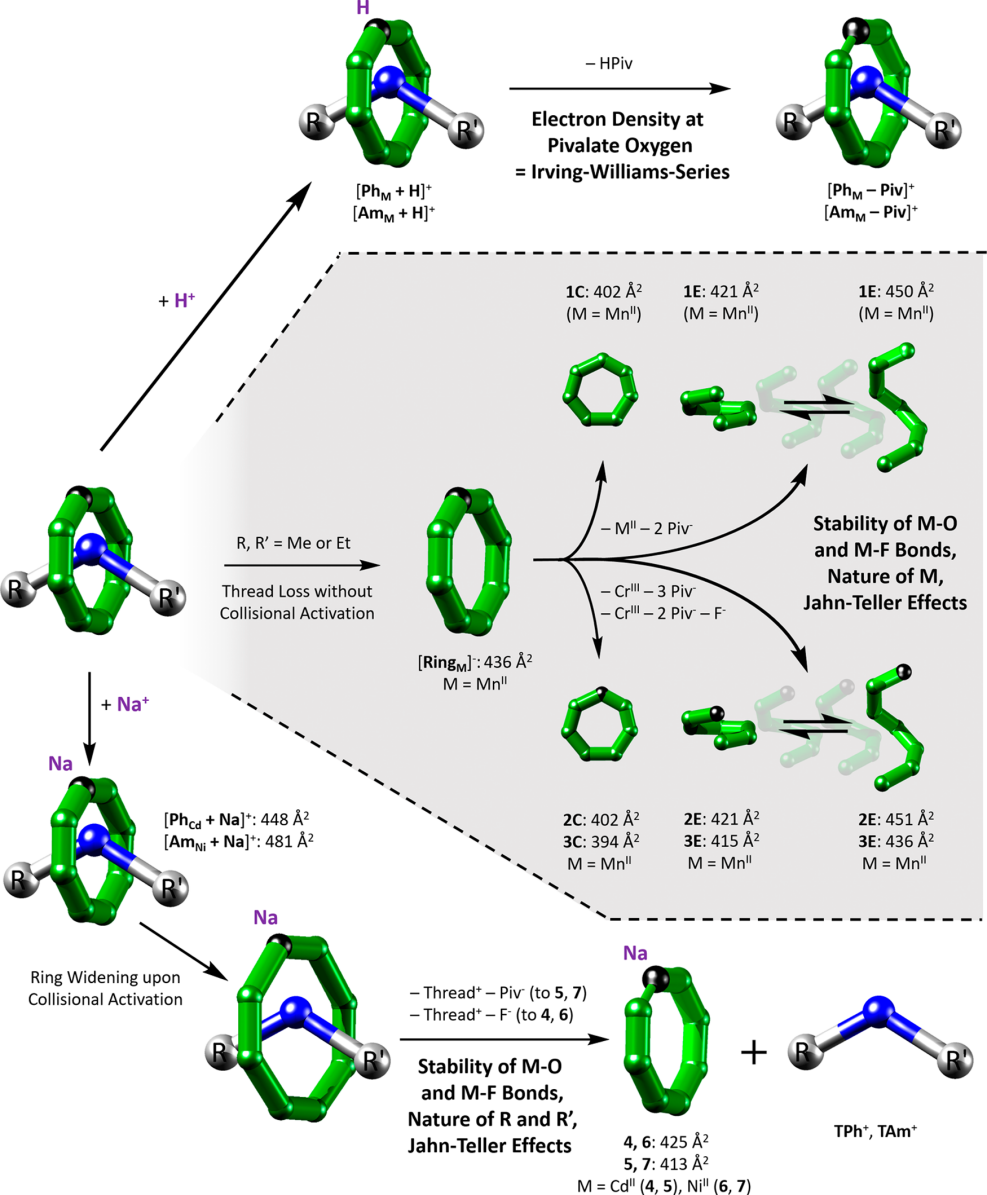
Disassembly of [**Ring_M_**]^−^, [**Ph_M_** + A]^+^ and [**Am_M_** + A]^+^ (A^+^ = H^+^, Na^+^) including fragmentation channels, determining stability factors, and CCS_N2_ (Cr: green, M: black, N: blue, Rest: white). Top: suggested loss of pivalic acid adjacent to M for the protonated species [**Ph_M_** + H]^+^ and [**Am_M_** + H]^+^. Center: predicted conformations of [**Ring_M_**]^−^ fragments **1**–**3** involving interconverting helical structures **E** and closed rings **C**. CCS_N2_ values are given for M = Mn^II^. ATD for other d-metals are found in the Supporting Information and the supplementary data set. Bottom: observed slipping mechanism for the sodiated forms [**Ph_M_** + Na]^+^ and [**Am_M_** + Na]^+^ including ring widening, showing the intact ring fragments **4**–**7** in the activated IM-MS spectra.

The trends in *E*_50_ values for [**Ring_M_**]^−^ (Figure 1d, Table S1) can be explained by considering the varying strengths of M–O and M–F bonds and the effective nuclear charge at the metal centers (Figure 4 center). The increased stability from Mn^II^ (3d^5^) to Ni^II^ (3d^8^) is attributable to the increasing effective nuclear charge and occupation of the stabilizing t_2g_-orbitals in the octahedral crystal field. For Cu^II^, Zn^II^, and Cd^II^, the reverse trend would be expected due to occupation of the destabilizing e_g_-orbitals and for Cd^II^ because of a larger ionic radius (Zn^II^ vs Cd^II^, Figure 1d and Table S1). Small deviations from this trend are shown by Mn^II^, which we would predict to be less stable than Fe^II^ (3d^5^ vs 3d^6^), and also by Cu^II^, which should be more stable than Zn^II^ (3d^9^ vs 3d^10^). We suggest that the repulsive forces between the pivalate ligands are less in [**Ring_Mn_**]^−^ than for the other rings because of the larger ionic radius for Mn^II^, compensating for the smaller Mn^II^–O bond energy.^30^ The lability of [**Ring_Cu_**]^−^ is a result of Jahn–Teller distortions causing the elongation of one of the F–Cu^II^–O axes,^31^ which is well-known from other Cu^II^ complexes and its impact on periodic stability trends previously reported.^32,33^ Jahn–Teller effects could also make [**Ring_Cu_**]^−^ more flexible, as observed before for related containing five-coordinate Cu^II^ sites.^34^ Notably, our data agree well with the M^II^ trend for water-exchange reaction rate constants of the hexaaqua ions [M^II^(H_2_O)_6_]^2+^ (Table S5, Figure S32), although a different stability trend from ESI-MS experiments has recently been reported for supramolecular terpyridine-based fractal complexes (Ni^II^ > Co^II^ > Zn^II^ > Fe^II^ > Cu^II^ > Cd^II^ > Mn^II^), indicating that the electronic environment of M^II^ may be altered in the case of some ligands.^35^

#### Sodiated Rotaxane Ions [**Ph_M_** + Na]^+^ and [**Am_M_** + Na]^+^

The loss of the thread must involve significant disruption of the ring, perhaps complete opening. This process would certainly involve lengthening or even breaking M^II^–O and M^II^–F, or less likely Cr^III^–O and Cr^III^–F bonds. We find a similar trend in stability, with respect to M^II^, for the sodiated species [**Ph_M_** + Na]^+^ and [**Am_M_** + Na]^+^ (Figures 2b and 4 bottom) to the data of the heterometallic ring anions [**Ring_M_**]^−^ (Figure 1d). Again, the trend agrees with water-exchange reaction rate constants of the hexaaqua ions [M^II^(H_2_O)_6_]^2+^ (Figure S33). A difference between the metal trends of the sodium adducts and the ring anions are the species with M = Zn^II^ and Cd^II^, where we observe a higher stability for M = Cd^II^ in both. Cd^II^ is the only 4d metal in this study and its bigger size and lower charge density might lead to a more favorable chelation of the sodium cation and/or stronger H–F hydrogen bonds in the thread resulting from weaker Cd^II^–F bonds.

The thread and its functionality have a significant impact on the *E*_50_ values of the sodiated ions [**Ph_M_** + Na]^+^ and [**Am_M_** + Na]^+^ (Figures 2b and 4 bottom, Table S1). The ions [**Am_M_** + Na]^+^ are all more stable than [**Ph_M_** + Na]^+^, which can be attributed to both steric and electronic effects. The *tert*-butyl groups in **TAm**^+^ are larger than the phenyl end groups in **TPh**^+^(36), making it harder for the thread to dissociate from the ring. Further, the amide end groups will form stronger charge–charge interactions with the macrocycle than the phenyl groups, making [**Am_M_** + Na]^+^ more stable. We also examined [**Ph_Fe(III)_**]^+^ and [**Am_Fe(III)_**]^+^ (Figure S30), which allows us to consider the effect of oxidation state when compared to the divalent metals M^II^. These exhibit strongly enhanced stabilities resulting from stronger charge–charge interactions of Fe^III^ and the absence of an additional cation, which presumably weakens the M–O and M–F bonds (Figure 2c).

#### Protonated Rotaxane Ions [**Ph_M_** + H]^+^ and [**Am_M_** + H]^+^

The fragmentation of the protonated rotaxane ions takes place at significantly lower *E*_50_ values (Figure 2c) than the sodiated forms and also leads to a different fragmentation pathway, which always involves the loss of one pivalic acid (Figure S24). Protonation followed by pivalic acid dissociation is more likely on a carboxylate oxygen bound to M^II^ than one adjacent to Cr^III^ due to higher charge density on the O-donor, which is why the *E*_50_ values of [**Ph_M_** + H]^+^ and [**Am_M_** + H]^+^ depend also on the divalent metal M^II^ (Figure 4 top). The *E*_50_ trend agrees well with the Irving–Williams series across the measured 3d-metal rings,^41^ with the exception of Cd^II^ which is not part of the Irving–Williams series as it is a 4d metal and shows a smaller *E*_50_ value than Zn^II^ (both d^10^).This is presumably because of its larger size that destabilizes the M^II^–O bond (Table S1). Another interesting correlation occurs with the *pK_A_* values of the hexaaqua ions [M^II^(H_2_O)_6_]^2+^ (Figure S34). The significantly lower *E*_50_ values of [**Ph_M_** + H]^+^ and [**Am_M_** + H]^+^ compared to the sodium adducts suggest a strong involvement of the proton, weakening the M–O bond significantly. As the metal with the lowest *pK_A_*(37) (M = Cu^II^) in [M^II^(H_2_O)_6_]^2+^ forms the weakest O–H and hence the strongest M–O bond, [**Ph_Cu_** + H]^+^ and [**Am_Cu_** + H]^+^ are expected to show the highest *E*_50_ values. This was confirmed by our experimental data and the complete correlation with the *pK_A_* trend of the hexaaqua ions, as shown in Figure S34.

### Ion Mobility Investigations and DFT Studies

#### Heterometallic Rings [**Ring_M_**]^−^

Activated ion mobility analysis of [**Ring_Mn_**]^−^ shows that the conformation is largely unaltered until fragmentation (Figure S10a). The ATD for each fragment are diagnostic and support disassembly into two conformational forms: a compact, rigid species **C** and an extended, flexible species **E** (Figure 1b). The relative intensities of the bimodal ATD found for product ions **1**–**3** alter with the number of pivalate ligands. For **1** and **2**, the metal centers proximal to the cleavage point now possess two pivalate groups and repulsive interactions between these bulky ligands could lead to the preference of extended conformations (**1E** and **2E**: CCS_N2_ ≈ 412–463 Å^2^, Figure 1b). Conversely, **3** has only one remaining pivalate ligand at the cleavage point, which could preferentially rearrange to be closer to the metal center, resulting in a more contracted conformation (**3C**: CCS_N2_ = 394 Å^2^). IMS^2^ experiments on the extended species **3E** did not provide any better separation after multiple passes in the cyclic drift ring, indicating that **3E** contains a dynamic equilibrium of interconverting conformers (Figure S15).

The collected data suggest that two disassembly mechanisms occur for [**Ring_M_**]^−^ (Figure 4 center), depending on both M (Figures S11–S13) and the collision energy (Figure S16). The metals with smaller *E*_50_ values, namely, Cu^II^, Zn^II^, and Cd^II^ (Figure 1d), have a stronger tendency to form **1** over **2** and **3** and also to yield **1C** over **1E**. This argues that this low energy mechanism leads only to minor ring disruption, favoring retention of compact conformers **C**. For the divalent metals with higher *E*_50_ values, more **1E** is seen, indicating a greater perturbation of the structure. The fact that Cu^II^, Zn^II^, and Cd^II^ decompose more to **C** than the other metals can be explained with their size (Cd^II^) and their tendency to form complexes with lower coordination numbers, facilitating the departure of the metal center without major ring disruption. Surprisingly, the divalent metal M in the precursor [**Ring_M_**]^−^, influences the conformations seen for the main fragment **1**, despite **1** only containing chromium (Figures S11–S13). This strongly suggests that the stability of the leaving group {MPiv_2_}, and the ease with which it can depart, is a driving factor for the preference of **C** or **E**, agreeing with the relative preference of Cu^II^, Zn^II^, and Cd^II^ for tetrahedral coordination geometries.

We hypothesize that the contracted conformers **C** are seven-membered rings (Figure 4 center), which have been reported once previously, for a Cr_6_Ce ring where the large ionic radius of the heterometal was crucial.^38^ Such species, assuming a similar connectivity as for [**Ring_M_**]^−^, can most easily form when exactly three ligands are present per metal center as in **2** and **3**, which would explain the preference for **1E** over **1C**. The broader peaks at higher CCS_N2_ could correspond to extended horseshoe structures with seven metal centers, which are more common than the closed seven-membered rings in solution.^39^

Informed by the structures obtained from DFT and their predicted CCS_N2_, we can rationalize the disassembly process of [**Ring_Mn_**]^−^. On the basis of systematic differences for each [**Ring_M_**]^−^, we apply a scaling factor of 0.92 to all predicted CCS_N2_ values for hypothetical fragment structures of **3** (Table S4). These scaled CCS_N2(s)_ values of a Cr_6_Mn-ring (Figure S17) and a slightly opened helix (Figure S20) agree well with the experimental CCS_N2_ of **3C**, suggesting that only minor ring perturbation takes place. By contrast, the CCS_N2(s)_ of the more open helical conformers (Figures S21 and S22) are in the experimental CCS_N2_ range of **3E**, agreeing with our predictions that these are highly disrupted opened ring structures.

#### Sodiated Rotaxane Ions [**Ph_M_** + Na]^+^ and [**Am_M_** + Na]^+^

The disassembly of the sodiated rotaxanes involves the loss of the thread (**TPh**^+^ or **TAm**^+^), for which two mechanisms can be considered. The first proceeds by an opening of the ring structure followed by thread release, and the second proceeds by a slipping mechanism through the cavity of the ring. The latter is relatively small for [**Ring_M_**]^−^ compared to common organic macrocycles, largely because of the bulky pivalate ligands, and [**Ring_M_**]^−^ therefore appears to hold the thread even with small stopper groups such as in **TPh^+^** and **TAm^+^** (Figure 2b). Space-filling models of the crystal structures **Ph_Cd_** and **Am_Ni_**(29) suggest that the cavity of the ring (diameter ≈ 3.5 Å) and the R and R’ groups (width ≈ 5.8–5.9 Å) will not permit a slipping mechanism (Figures S35 and S36, Table S6). We previously studied the kinetic stability of **Am_Co_** where we added a similar, isotopically labeled rotaxane at 60 °C. No ring or thread exchange occurred between the molecules after stirring the solution for one week.^29^ These data did not point to a slipping mechanism for disassembly in solution, but ambiguity in the analysis from new bulk phase measurements warranted further investigation.

The observed gas phase stability trends for [**Ph_M_** + Na]^+^ and [**Am_M_** + Na]^+^ (Figure 2c) are similar to the one obtained for the [**Ring_M_**]^−^ series (Figure 1d) as the stability of M–O and M–F bonds likely determines the *E*_50_ value here as well. This can be due to either bond breaking or lengthening and does not help to determine the dethreading mechanism. The evidence from ion mobility measurements is more useful, and following thread loss, the rotaxane ring fragments (**4**–**7**) present have narrow ATD, indicative of compact structures with no evidence of extended conformers as seen for [**Ring_Mn_**]^−^ (Figure 1b). In the ion mobility heat maps of [**Ph_Cd_** + Na]^+^ and [**Am_Ni_** + Na]^+^ (Figure 3a), the observed precursor ATD narrows between *E*_lab_ = 60–90 eV, which suggests minimal disruption of the ring structure prior to the loss of thread.

Our hypothesis, therefore, that the ring has widened prior to thread release is further strengthened by comparison of the CCS_N2_ distributions of fragments **4** and **6** (Figure 3b) with corresponding [**Ring_M_**]^−^ anions (Figure 1b), where we find highly similar experimental CCS_N2_ values for the rotaxane ring fragment ions [**(Ring_M_** − F) + Na]^+^ (**4**, **6**) after the thread has been lost. As we assign the [**Ring_M_**]^−^ ions as closed rings, we attribute a slipping mechanism to the fragmentation of the sodiated forms [**Ph_M_** + Na]^+^ and [**Am_M_** + Na]^+^ (Figure 4 bottom), although ring opening followed by thread release and fast reclosing of the ring is also possible. The almost identical CCS_N2_ values of the fragments **4**, **6** and **5**, **7** (Figure 3b) can be explained by their nearly equal composition (only difference in M) and strongly implies that the same dethreading mechanism occurs for both ions [**Am_Ni_** + Na]^+^ and [**Ph_Cd_** + Na]^+^. Considering that the thread end groups in both **Am_M_** and **Ph_M_** are significantly larger than the ring diameter, as shown by space-filling models from the crystal structures (Figures S35 and S36, Table S6), this result is noteworthy and warrants further investigation.

## Conclusions

We have shown that energy-resolved MS^2^ and IM-MS coupled with DFT combine effectively to characterize polymetallic complexes in the gas-phase, yielding information on the compounds’ disassembly, energetics, and conformational dynamics. Our study demonstrates that these methods, more commonly applied to the disassembly of protein complexes,^6,40−65^ can be usefully applied for supramolecular and inorganic chemistry. The results show that the stability of the studied ring and rotaxane ions is tuned by altering the d-metal composition in the heterometallic ring, the end groups in the thread, and the charge carrying ion, providing a framework to follow in for the future design of self-assembled polymetallic complexes.

IM-MS was applied to investigate the dissociation mechanism of the rotaxane ions in the gas phase, suggesting that the thread slips through the cavity of the ring after bond lengthening near M. Examination of the [**Ring_M_**]^−^ disassembly with IM-MS disclosed two mechanistic routes, leading to compact seven-membered rings as well as conformationally dynamic open horseshoes. Perhaps most curiously, we find that the structure of the homometallic fragment **1** = [Cr_7_F_8_Piv_14_]^−^ is differentiated into compact or extended product ions depending on the divalent metal in the precursor ion, even though M is no longer present.

For all studied species, the trends in stability and ensuing disassembly mechanisms can be rationalized using concepts from crystal field theory, demonstrating that classic observations such as Jahn–Teller effects and the Irving-Williams series are applicable to large polymetallic compounds. Extending this to include ligand field theory would suggest other factors such as interelectronic repulsion (the nephelauxetic effect), spin–orbit coupling and variation in bond lengths could be evaluated.^42^ In the future, it may be possible to compare the simple crystal field theory explanation here with a more sophisticated ligand field theory approach. For now, we conclude that the use of IM-MS coupled with *ab initio* calculations to examine (metallo-)supramolecular complexes has considerable promise.^13^

## Experimental/Methods

### Synthesis and Materials

The rotaxanes **Am_M_** (M = Mn^II^, Fe^II^, Co^II^, Ni^II^, Cu^II^, Zn^II^, Cd^II^)^29^ and [NH_2_(CH_3_)_2_][**Ring_M_**] (M = Mn^II^, Co^II^, Ni^II^, Cu^II^, Zn^II^, Cd^II^),^34,43^ as well as [NH_2_(C_2_H_5_)_2_][**Ring_Fe_**]^43^ were prepared using methods previously reported by our group. The rotaxane family **Ph_M_** (M = Mn^II^, Fe^II^, Co^II^, Ni^II^, Cu^II^, Zn^II^, Cd^II^) and the corresponding secondary amine (deprotonated form of **TPh**^+^) were synthesized according to experimental procedures described in the SI (Figure S37). Crystal structures were obtained for **Ph_M_** (M = Mn^II^, Fe^II^, Co^II^, Ni^II^, Cu^II^, Zn^II^, Cd^II^, Figures S38–S44, Table S7).

All reagents and solvents were purchased from Sigma-Aldrich, Alfa, Fisher Scientific, or Fluorochem and used without further purification. The syntheses of the hybrid organic–inorganic rotaxanes were carried out in Erlenmeyer Teflon FEP flasks supplied by Fisher Scientific. Column chromatography was performed using either 40–63 μm silica from Sigma-Aldrich or a Grace Reverelis X2 Autocolumn with Grace Reverelis NP cartridges.

### Sample Preparation

Samples were typically prepared in 500 μM NaI and 7:3 methanol/toluene (**Am_M_**) or 4:1 toluene/methanol (**Ph_M_**, [NH_2_(CH_3_)_2_][**Ring_M_**], [NH_2_(C_2_H_5_)_2_][**Ring_Fe_**]) respectively. Concentrations of 2 μM (Q Exactive UHMR) and 10 μM (Cyclic IMS) were typically used. When necessary, 0.5−1% formic acid was added to enhance the signal of the protonated ions. For the activated ion mobility data of [**Ring_Cd_**]^−^, a sample of **Am_Cd_** was used instead of [NH_2_(CH_3_)_2_][**Ring_Cd_**].

### Mass Spectrometry (MS) and Ion-Mobility Mass Spectrometry (IM-MS)

All samples were ionized with a nanoESI source and sprayed from borosilicate glass capillaries (World Precision Instruments, Stevenage, UK), which were pulled on a Flaming/Brown P-2000 laser puller (Sutter Instrument Company, Novato, CA, US). A potential of 1.0–1.5 kV was applied through a platinum wire (diameter = 0.125 mm, Goodfellow, Huntingdon, UK) inserted into the nanoESI capillaries. The source temperature was set to 23° (Cyclic IMS) and 30° (Q Exactive UHMR), respectively.

The Q Exactive ultra-high mass range (UHMR) hybrid quadrupole-Orbitrap mass spectrometer (Thermo Fisher)^44^ was used for the derivation of all *E*_50_ values *via* tandem mass spectrometry (MS^2^) experiments involving collision-induced dissociation (CID) studies. The complete isotopic envelope of the target ions were typically *m*/*z*-selected in a quadrupole filter, accelerated to a determined kinetic energy (*E*_lab_: 0–300 eV) and subsequently injected into the high-energy C-trap dissociation (HCD) cell, which contained nitrogen gas at a constant pressure (trapping gas pressure parameter: 2.0). Fragment ions as well as precursor ions that did not fragment, were mass-analyzed in the Orbitrap mass analyzer (resolution: 25000, AGC target: 3E6 ions, maximum inject time: 100 ms).

Ion mobility mass spectrometry (IM-MS) experiments were performed on a Select Series Cyclic IMS (Waters).^45^ After the transfer to the gas phase (cone voltage: 20–60 V, source offset: 10–20 V, purge gas: 50–300 L/h), ions of interest were isolated by a quadrupole mass filter, activated in a trap cell where appropriate (trap bias: 2 V, *E*_lab_: 0–200 eV) and subsequently injected into the cyclic ion mobility drift ring. In this region, ions were separated by using a nonuniform electric field under a constant nitrogen pressure. Traveling waves (TW, height: 20 V) pushed the ions through the cyclic drift region. Unless noted otherwise, ions traveled one pass in the cyclic drift ring (“single path”, separation time: 2 ms) and were subsequently transferred (transfer energy: 4–15 V) to a time-of-flight mass analyzer. Details of this method known as traveling-wave ion mobility spectrometry (TWIMS) can be found elsewhere.^3,46−48^

### Data Processing

Mass spectra were recorded for different kinetic energies, and the survival yield (SY) of each precursor ion was calculated from its absolute intensity (*I*_P_) and the sum of the fragment ion intensities (*I*_F_) according to eq 1:
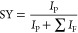
1

Additionally, laboratory kinetic energies *E*_lab_ were converted to center-of-mass energies *E*_com_ using eq 2. This relationship assumes a single collision of the stationary target gas with the mass *m*_g_, for this work nitrogen, with the accelerated precursor ion of the mass *m*_p_. Under these conditions, the maximum amount of kinetic energy accessible for the conversion to internal energy is given by *E*_com_.^49,50^
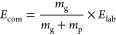
2

The center-of-mass energy where SY = 0.5 (or 50%) can be defined as *E*_50_, which is known as a relative measure of precursor ion stability in the gas phase. Experimental parameters (trapping gas pressure, temperature, and pre-CID voltages) are maintained constant in order to obtain comparable and meaningful *E*_50_ values across different precursor ions; however comparisons across different instruments are not trivial.^50^*E*_50_ values are derived from plots of SY vs *E*_com_ by using a fit with a sigmoidal Hill function (Hill1 function in OriginPro 2020b). For some of the studied protonated forms, contaminating species overlapped and showed significantly different stabilities. In these cases, the share of the contamination was subtracted in the survival yield plots before fitting.

Activated ion mobility mass spectrometry data obtained from a Select Series Cyclic IMS instrument (Waters) were not converted to *E*_com_. Experimentally obtained arrival time distributions were converted to nitrogen collisional cross sections (^TW^CCS_N2_, TW = “Traveling Waves”) *via* published calibration procedures.^51^ The Agilent tune mix was used for all ^TW^CCS_N2_ calibrations.^52^

### Density Functional Theory and Collision Cross Section Calculations

All DFT calculations were carried out with Gaussian 16^53^ utilizing the B3LYP exchange-correlation functional with the Grimme D3 empirical dispersion correction.^54^ An effective core potential and its associated split valence basis set were used for transition metals (LANL2DZ),^55^ and a 6-31G(d) basis set on other atoms. All structures were optimized to the default convergence criteria (RMS force <3·10^–4^ E_h_/a_0_), and the conformers were confirmed to be the minima by vibrational analysis with corresponding formate models (O_2_CH^–^ instead of O_2_C^*t*^Bu). Metal electronic states were high spin, as found experimentally, with low deviations from <*S*^2^> although they were ferromagnetically coupled. Atomic charges were obtained for the optimized structures at the same DFT level using the Merz–Kollman method with UFF-based radii as implemented in Gaussian 16.

Theoretical collision cross section values (^TH^CCS_N2_, TH = “Theoretical”) were obtained from the software IMoS by using the trajectory method in nitrogen gas including quadrupole potential (number of orientations: 3, gas molecules per orientation: 300,000, temperature: 298 K, pressure: 101,325 Pa = 1 atm).^24^

### Crystallographic Data

Single crystal XRD data was collected on an Agilent SuperNova CCD diffractometer with Mo Kα radiation (λ = 0.71073 Å) and a Rigaku FR-X with Cu Kα radiation (λ = 1.5418 Å) equipped with a Hypix6000HE detector. Data was measured using the CrysAlisPro suite of programs^56^ and was solved using the SHELXL and Olex 2 suite of programs.^57,58^

## Data Availability

The supplementary data set is available on Figshare 10.6084/m9.figshare.20324448 and contains the raw ion mobility mass spectrometry and mass spectrometry data files as well as the outputs from DFT calculations.

## Supporting Information Available

Mass spectrometry, tandem mass spectrometry, and ion mobility mass spectrometry data of [**Ring_M_**]^−^, [**Ph_M_** + A]^+^_,_ and [**Am_M_** + A]^+^ as well as related data; density functional theory studies and predicted collision cross sections of [**Ring_M_**]^−^, its conformers and of fragment **3;** crystal data and studies of **Ph_M_** and **Am_M_**; synthetic procedure for **Ph_M_** (PDF)
