# Supplementary material for: Disassembly Mechanisms and Energetics of Polymetallic Rings and Rotaxanes
Source: J Am Chem Soc. 2022 Dec 2;144(49):22528–39. doi: 10.1021/jacs.2c07522 (PMC9756338; doi:10.1021/jacs.2c07522)
Supplement: Supplementary file 1 — ja2c07522_si_001.pdf [file ja2c07522_si_001.pdf]

Supporting Information

Disassembly Mechanisms and Energetics of Polymetallic Rings and Rotaxanes

Niklas Geue[1], Tom S. Bennett[2], Alexandra-Ana-Maria Arama[2], Lennart A. I. Ramakers[1], George F. S. Whitehead[2], Grigore A. Timco[2], P. B. Armentrout[3], Eric J. L. McInnes[2], Neil A. Burton[2], Richard E. P. Winpenny[2] and Perdita E. Barran[1,*]

[1]Michael Barber Centre for Collaborative Mass Spectrometry, Manchester Institute of Biotechnology, Department of Chemistry, The University of Manchester, 131 Princess Street, Manchester, M1 7DN, UK. [2]Department of Chemistry, The University of Manchester, Oxford Road, Manchester, M13 9PL, UK. [3]Department of Chemistry, University of Utah, Salt Lake City, Utah 84112, USA.

[*]Corresponding Author: perdita.barran@manchester.ac.uk

Table of Contents

*Figure S1: a) Mass spectra of $[NH_2(CH_3)_2]$[**Ring$_{Mn}$**] = **Me$_{Mn}$** and b) Comparison of predicted and observed isotopic distributions for [**Ring$_{Mn}$**]$^-$* 4

*Table S1: E_{50} values of [**Ring$_M$**]$^-$, [**Ph$_M$** + A]$^+$ and [**Am$_M$** + A]$^+$* 5

*Table S2: Experimental $^{TW}CCS_{N2}$ and predicted $^{TH}CCS_{N2}$ values of [**Ring$_M$**]$^-$* 6

*Figure S2: DFT optimised structure of [**Ring$_{Mn}$**]$^-$* 7

*Figure S3: DFT optimised structure of ring conformer 1 of [**Ring$_{Mn}$**]$^-$* 8

*Figure S4: DFT optimised structure of ring conformer 2 of [**Ring$_{Mn}$**]$^-$* 9

Figure S5: View orientation for used nomenclature 10

*Figure S6: DFT optimised structure of open 0a of [**Ring$_{Mn}$**]$^-$* 11

*Figure S7: DFT optimised structure of open 0b of [**Ring$_{Mn}$**]$^-$* 12

Table S3: $^{TH}CCS_{N2}$ values of ring conformers 1 and 2 and open structures 0a and 0b 13

*Figure S8: $^{TH}CCS_{N2}$ values of [**Ring$_{Ni}$**]$^-$ for different effective temperatures* 14

*Figure S9: Averaged metal-metal distances for [**Ring$_M$**]$^-$* 15

*Figure S10: a) Collision Energy vs. ATD heat map for [**Ring$_{Mn}$**]$^-$ and its fragments. b) Corresponding MS^2 spectra at selected E_{lab}* 17

*Figure S11: Stacked ATD plot for **1** at E_{lab} = 50 – 150 eV following activation of [**Ring$_{Cu}$**]$^-$* 18

*Figure S12: Stacked ATD plot for **1** at E_{lab} = 50 – 150 eV following activation of [**Ring$_{Mn}$**]$^-$* 19

*Figure S13: Stacked ATD plot for **1** at E_{lab} = 50 – 150 eV following activation of [**Ring$_{Ni}$**]$^-$* 20

*Figure S14: IMS^2 separation of **3C** at E_{lab} = 110 eV* 21

*Figure S15: IMS^2 separation of **3E** at E_{lab} = 110 eV* 22

*Figure S16: Share of **C** (black) and **E** (red) of fragment ions **1**-**3** in dependence of E_{lab}* 23

*applied to the precursor [**Ring$_{Mn}$**]$^-$.* 23

*Table S4: Predicted $^{TH}CCS_{N2}$ values of different candidate structures for **3*** 24

*Figure S17: DFT optimised structure of closed ring **3$_I$*** 25

*Figure S18: DFT optimised structure of open **3$_{II}$*** 26

*Figure S19: DFT optimised structure of open **3$_{III}$*** 27

*Figure S20: DFT optimised structure of open **3$_{IV}$*** 28

*Figure S21: DFT optimised structure of open **3$_V$*** 29

*Figure S22: DFT optimised structure of open **3$_{trans}$*** 30

*Figure S23: Mass spectra of **Ph$_{Co}$** and **Am$_{Co}$** in NaI.* 31

*Figure S24: a) MS^2 spectra of [**Ph_{Mn}** + H]$^+$ at E_{lab} = 25 eV and [**Ph_{Mn}** + Na]$^+$ at E_{lab} = 90 eV. b) Normalised survival yield vs. E_{com} for [**Ph_{Mn}** + H]$^+$ and [**Ph_{Mn}** + Na]$^+$.......................................32*

*Figure S25: MS^2 spectrum of [**Ph_{Mn}** + H]$^+$ at E_{lab} = 110 eV. 33*

*Figure S26: MS^2 spectrum of [**Ph_{Mn}** + Na]$^+$ at E_{lab} = 150 eV. 34*

*Figure S27: MS^2 spectrum of [**Ph_{Ni}** + H]$^+$ at E_{lab} = 25 eV...35*

*Figure S28: Suggested rearrangement mechanism for [**Ph_M** + H]$^+$..36*

*Figure S29: MS^2 spectrum of [**Am_{Cu}** + Na]$^+$ at E_{lab} = 100 eV..37*

*Figure S30: MS^2 spectrum of [**$Ph_{Fe(III)}$**]$^+$ at E_{lab} = 140 eV. 39*

*Figure S31: MS^2 spectrum of [**Am_{Ni}** + Na]$^+$ at E_{lab} = 110 eV and [**Ph_{Cd}** + Na]$^+$ at E_{lab} = 80 eV...40*

Table S5: Water-exchange reaction rate constants k_{H2O} and pK_A values of hexaaqua ions....41

*Figure S32: E_{50} values of [**$Ring_M$**]$^-$ vs. logarithm of the water-exchange reaction rate constants of $[M^{II}(H_2O)_6]^{2+}$...42*

*Figure S33: E_{50} values of [**Ph_M** + Na]$^+$ and [**Am_M** + Na]$^+$ vs. logarithm of the water-exchange reaction rate constants of $[M^{II}(H_2O)_6]^{2+}$..43*

*Figure S34: E_{50} values of [**Ph_M** + H]$^+$ and [**Am_M** + H]$^+$ vs. pK_A values of $[M^{II}(H_2O)_6]^{2+}$...............44*

*Figure S35: Space-filling model of **Ph_{Cd}**..45*

*Figure S36: Space-filling model of **Am_{Ni}**...46*

*Table S6: Estimated cavity diameters and stopper widths of **Ph_{Cd}** and **Am_{Ni}**..........................47*

*Figure S37: Synthetic procedure for rotaxane family **Ph_M**...48*

*Figure S38: Single crystal X-ray structure of **Ph_{Mn}** 49*

*Figure S39: Single crystal X-ray structure of **Ph_{Fe}**...50*

*Figure S40: Single crystal X-ray structure of **Ph_{Co}** 51*

*Figure S41: Single crystal X-ray structure of **Ph_{Ni}**..52*

*Figure S42: Single crystal X-ray structure of **Ph_{Cu}** 53*

*Figure S43: Single crystal X-ray structure of **Ph_{Zn}** 54*

*Figure S44: Single crystal X-ray structure of **Ph_{Cd}** 55*

*Table S7: Crystal data and structure refinement for **Ph_M**...56*

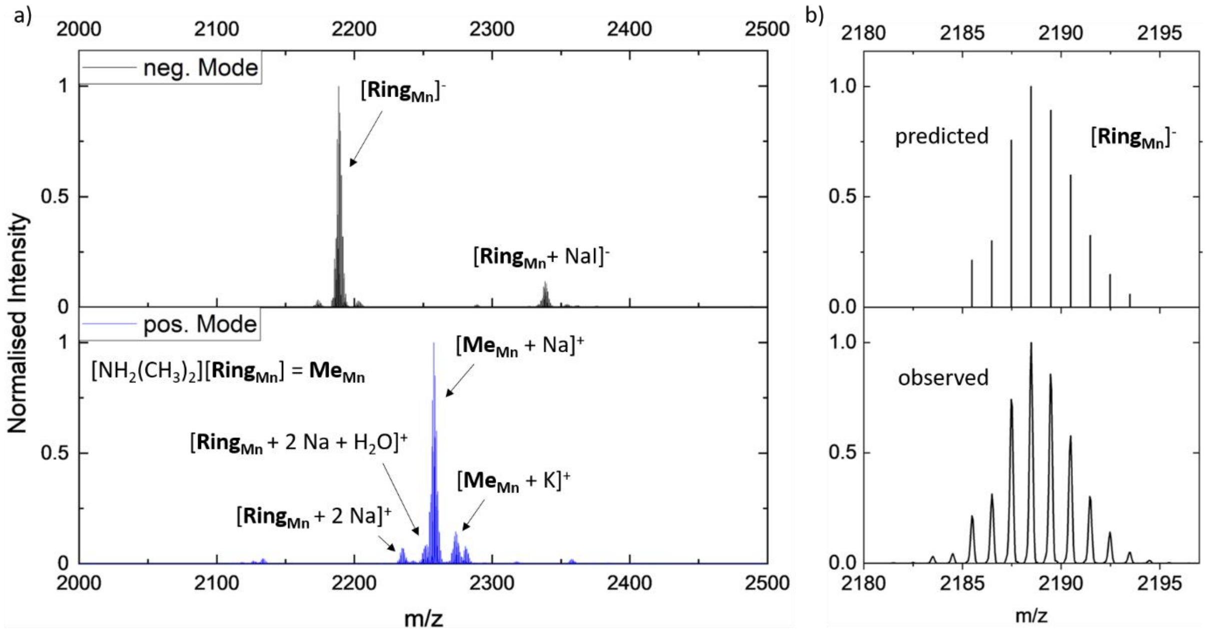

Figure S1: a) Mass spectra of $[NH_2(CH_3)_2]$[**Ring$_{\mathbf{Mn}}$**] = **Me$_{\mathbf{Mn}}$** and b) Comparison of predicted and observed isotopic distributions for [**Ring$_{\mathbf{Mn}}$**]$^-$ (m/z = 2188). In negative mode (top), the mass spectrum shows [**Ring$_{\mathbf{Mn}}$**]$^-$ as the base peak, although no further collisional activation was applied. In positive mode (bottom), the alkali metal species [**Me$_{\mathbf{Mn}}$** + Na]$^+$ and [**Me$_{\mathbf{Mn}}$** + K]$^+$ were observed as well as the ions [**Ring$_{\mathbf{Mn}}$** + 2 Na]$^+$ and [**Ring$_{\mathbf{Mn}}$** + 2 Na + H_2O]$^+$, in which the thread was exchanged for Na^+ and Na^+/H_2O, respectively. 2 µM **Me$_{\mathbf{Mn}}$** were used in 500 µM NaI and 4:1 toluene/methanol.

Table S1: E_{50} values of [**Ring$_M$**]$^-$, [**Ph$_M$** + A]$^+$ and [**Am$_M$** + A]$^+$ ($A^+ = H^+$, Na^+) including experimental error.

**E_{50} in eV**	**Mn^{II}**	**Fe^{II}**	**Co^{II}**	**Ni^{II}**	**Cu^{II}**	**Zn^{II}**	**Cd^{II}**
[**Ring$_M$**]$^-$	1.267 ± 0.002	1.231 ± 0.002	1.257 ± 0.002	1.326 ± 0.002	1.084 ± 0.004	1.146 ± 0.003	1.112 ± 0.003
[**Ph$_M$** + H]$^+$	0.283 ± 0.003	-	0.288 ± 0.005	0.331 ± 0.003	0.478 ± 0.014	0.377 ± 0.008	0.242 ± 0.002
[**Am$_M$** + H]$^+$	0.248 ± 0.003	-	0.256 ± 0.002	0.209 ± 0.002	0.450 ± 0.004	0.336 ± 0.002	0.218 ± 0.003
[**Ph$_M$** + Na]$^+$	1.021 ± 0.001	0.953 ± 0.001	0.940 ± 0.002	1.066 ± 0.002	0.772 ± 0.002	0.802 ± 0.002	0.889 ± 0.002
[**Am$_M$** + Na]$^+$	1.098 ± 0.005	1.094 ± 0.002	1.092 ± 0.005	1.221 ± 0.003	0.912 ± 0.004	0.902 ± 0.003	1.028 ± 0.007

Table S2: Experimental $^{TW}CCS_{N2}$ and predicted $^{TH}CCS_{N2}$ values of [$\mathbf{Ring_M}$]$^-$ (M = Mn^{II}, Fe^{II}, Co^{II}, Ni^{II}, Cu^{II}, Zn^{II}, Cd^{II}) including error (TW = “Travelling Waves”, TH = “Theoretical”).

**Divalent Metal M**	**$^{TW}CCS_{N2}$ in Å²**	**$^{TH}CCS_{N2}$ in Å²**
Mn^{II}	435.6 ± 2.2	470.7 ± 1.3
Fe^{II}	434.1 ± 2.3	470.8 ± 1.5
Co^{II}	434.8 ± 1.3	469.8 ± 1.2
Ni^{II}	432.7 ± 2.4	469.0 ± 1.4
Cu^{II}	432.7 ± 2.0	468.6 ± 2.1
Zn^{II}	434.8 ± 1.8	471.6 ± 1.2
Cd^{II}	437.0 ± 2.5	470.3 ± 1.7

All [$\mathbf{Ring_M}$]$^-$ show unimodal $^{TW}CCS_{N2}$ distributions with similar widths except for M = Cd^{II} that appears with a broader, more asymmetric peak shape. The $^{TW}CCS_{N2}$ differences between the ions [$\mathbf{Ring_M}$]$^-$ are minor, which is not surprising considering their almost identical structure. The slightly higher $^{TW}CCS_{N2}$ for [$\mathbf{Ring_{Cd}}$]$^-$ and less pronounced for [$\mathbf{Ring_{Mn}}$]$^-$ can be attributed to larger covalent radii of Cd^{II} and Mn^{II}. This seems to lead to slightly more extended structures caused by longer ligand-ligand distances,[1] which agreed with the averaged metal-metal distances of the B3LYP optimised [$\mathbf{Ring_M}$]$^-$ structures (Figure S2) and those obtained from $\mathbf{Am_M}$ crystal structures (Figure S9).[2]

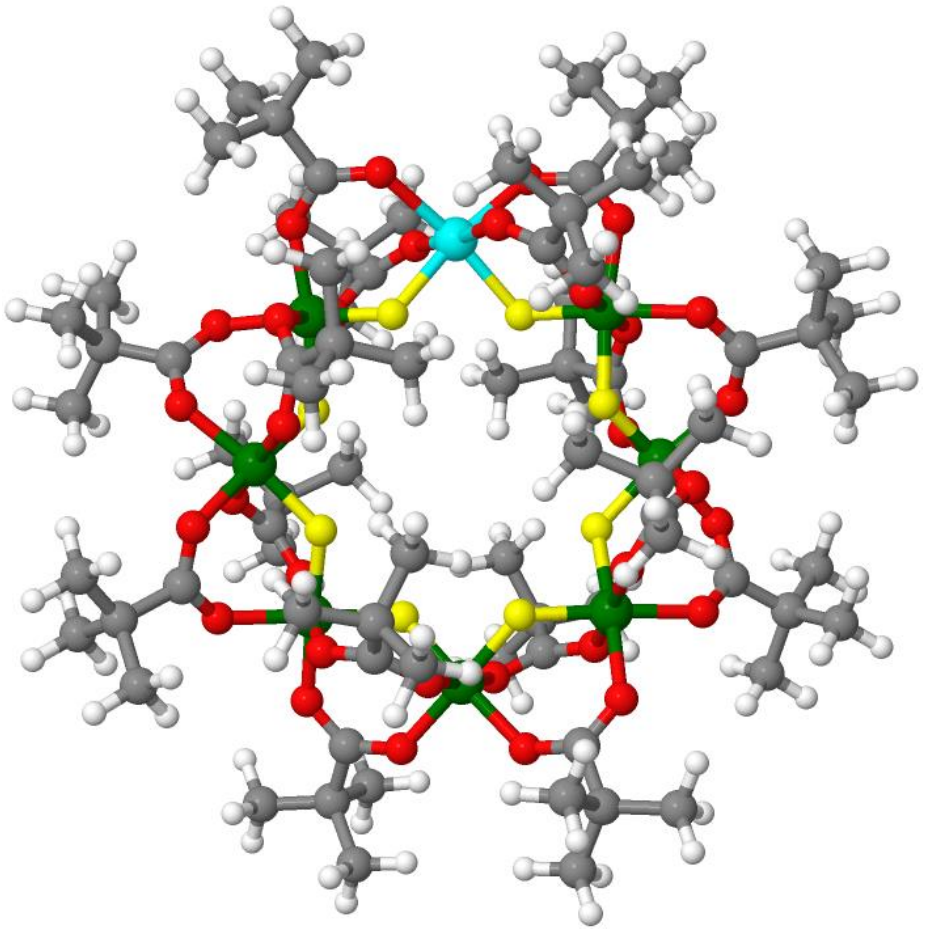

Figure S2: DFT optimised structure of [**Ring$_{\mathbf{Mn}}$**]$^-$ (Mn: cyan, Cr: green, F: yellow, O: red, C: grey, H: white). Optimized coordinates, ESP charges and minimum energies can be found in the Supplementary Dataset for all [**Ring$_{\mathbf{M}}$**]$^-$.

We have investigated a number of low lying [**Ring$_{\mathbf{M}}$**]$^{-}$ conformers originating from minor perturbations in the coordination of one or more of the fluorides, which couple to a variation in the coordination or orientation of the pivalate groups, as well as a range of possible methyl and *tert*-butyl rotamers. Although there is some uncertainty in the exact global minimum for different M, we note that there is little difference in $^{TH}CCS_{N2}$ values of the selected conformers of [**Ring$_{\mathbf{Mn}}$**]$^{-}$ (Figures S3, S4 and Table S3), particularly for the most likely ring conformer 2 (Figure S4), approximately within 10 kJ/mol.

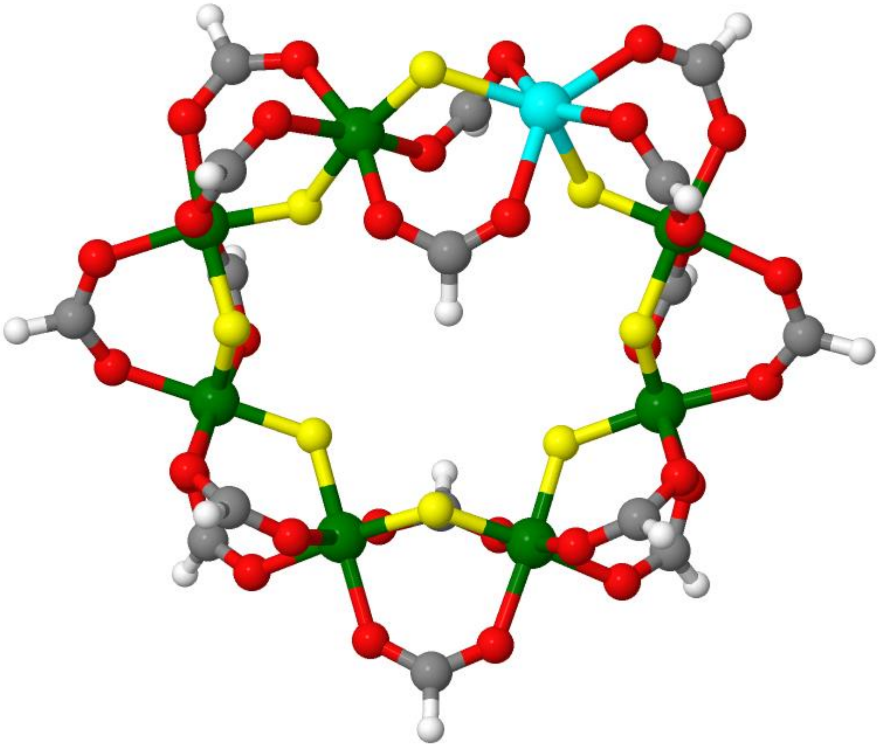

Figure S3: DFT optimised structure of ring conformer 1 of [**Ring$_{\mathbf{Mn}}$**]$^{-}$ (Mn: cyan, Cr: green, F: yellow, O: red, C: grey, H: white). *Tert*-butyl groups were replaced with hydrogen for clarity. Optimized coordinates, ESP charges and minimum energies can be found in the Supplementary Dataset.

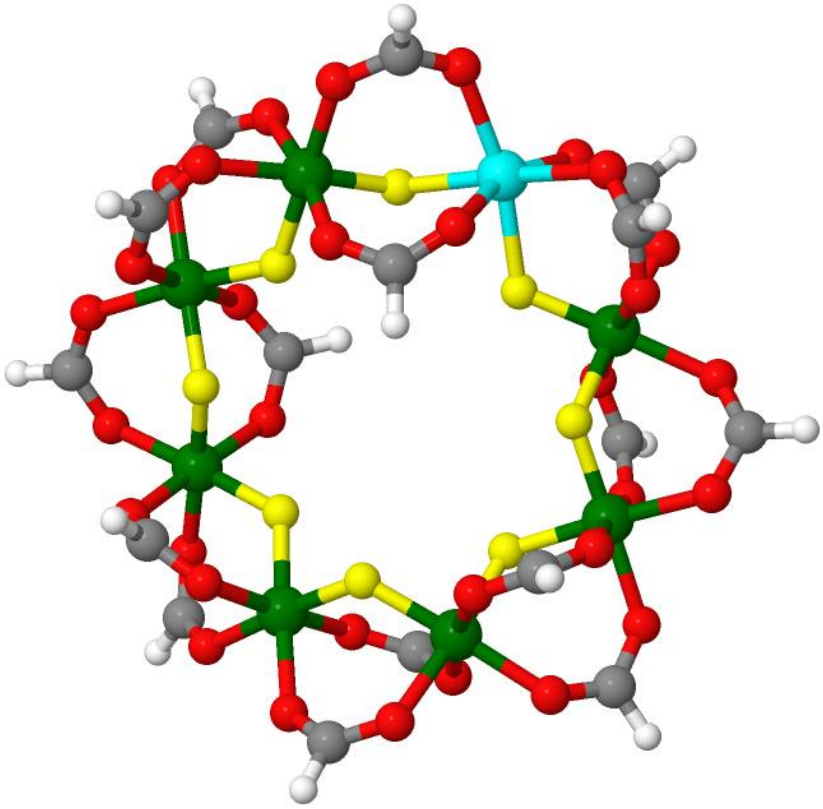

Figure S4: DFT optimised structure of ring conformer 2 of [$\mathbf{Ring_{Mn}}$]$^-$ (Mn: cyan, Cr: green, F: yellow, O: red, C: grey, H: white). *Tert*-butyl groups were replaced with hydrogen for clarity. Optimized coordinates, ESP charges and minimum energies can be found in the Supplementary Dataset.

A nomenclature was developed in order to differentiate between the analysed open structures of [**Ring$_{\mathbf{Mn}}$**]$^-$ (Figures S6, S7 and Table S3) and isomers of fragment **3** (Figures S17-S22 and Table S4). Their bonding situation is similar and almost all of them involve metal centres in an octahedral environment, with two fluoride atoms arranged in a cis configuration. For each conformer, labels *a* (axial) or *e* (equatorial) were assigned for every metal centre in clockwise direction, starting from Cr2 (terminal chromium ion in open structures). The structures were rotated to view along the bond formed by the terminal fluorine ion and the first chromium centre, as illustrated in Figure S5. In this orientation, the second fluoride ion is located in the equatorial position, *e2,* and the first centre was labelled *e2* as a reference. The view was then altered along the second fluoride ion and a label was assigned for the corresponding centre. For each step, the structure was rotated so that the axial carboxyl bridge, pointing towards the viewer, alternated between the positions *a1* and *a3* and the fluorine atom was located in either one of the two positions, *e2* or *a3*. For example, the designated name for open 0a is *e2-e2-e2-e2-a3-e2-e2*, indicating that a rotation was made between the centres Cr6 and Cr7 (Figure S6).

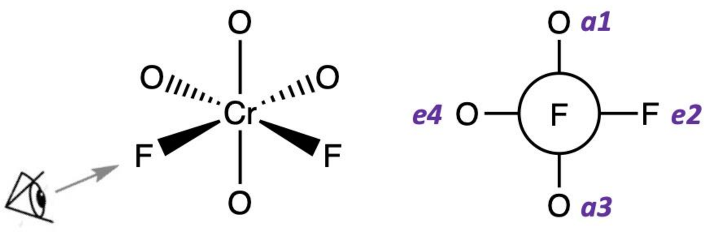

Figure S5: View orientation for used nomenclature. Left: perspective drawing, right: Newman projection.

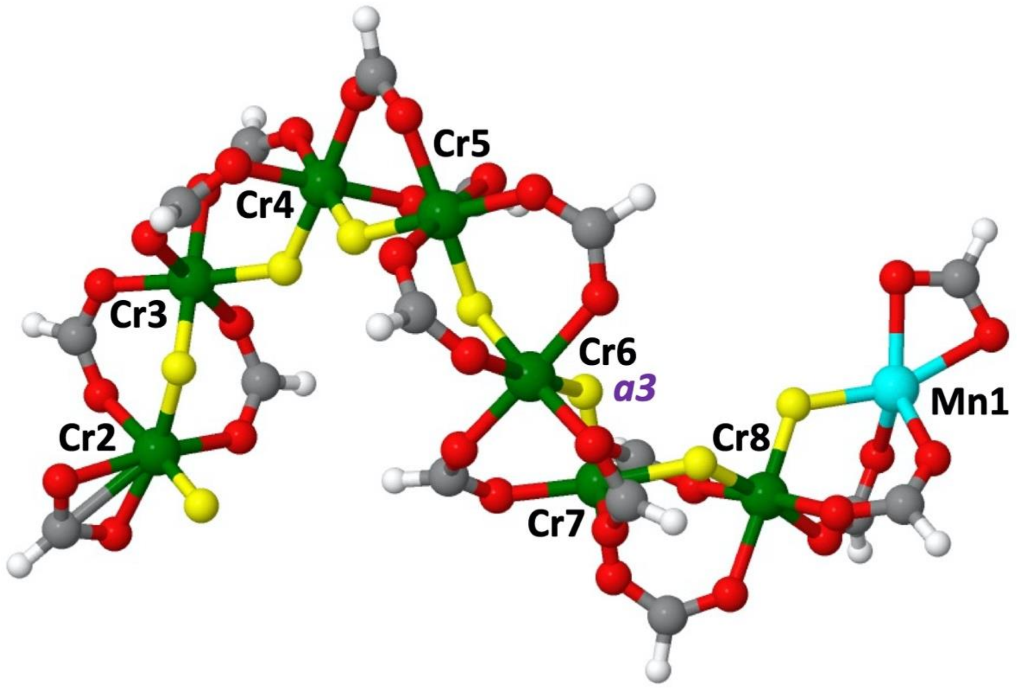

Figure S6: DFT optimised structure of open 0a of [**Ring$_{\mathbf{Mn}}$**]$^{-}$ (Mn: cyan, Cr: green, F: yellow, O: red, C: grey, H: white). *Tert*-butyl groups were replaced with hydrogen for clarity. Optimized coordinates, ESP charges and minimum energies can be found in the Supplementary Dataset.

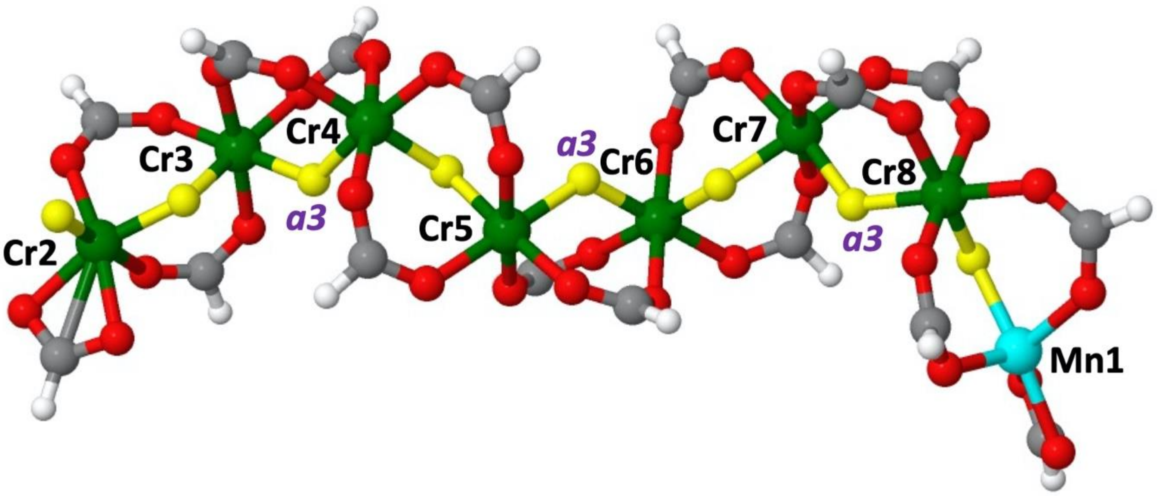

Figure S7: DFT optimised structure of open 0b of [**Ring$_{\mathbf{Mn}}$**]$^-$ (Mn: cyan, Cr: green, F: yellow, O: red, C: grey, H: white). *Tert*-butyl groups were replaced with hydrogen for clarity. Optimized coordinates, ESP charges and minimum energies can be found in the Supplementary Dataset.

Table S3: $^{TH}CCS_{N2}$ values of ring conformers 1 and 2 and open structures 0a and 0b including error. All structures are conformers or isomers of [**$Ring_{Mn}$**]$^{-}$.

**Structure**	**Nomenclature**	**$^{TH}CCS_{N2}$ in Å²**
Ring Conformer 1	-	461.3 ± 1.2
Ring Conformer 2	-	464.6 ± 1.3
Open 0a	e2-e2-e2-e2-a3-e2-e2	507.1 ± 2.1
Open 0b	e2-a3-e2-a3-e2-a3-e2	516.5 ± 1.5

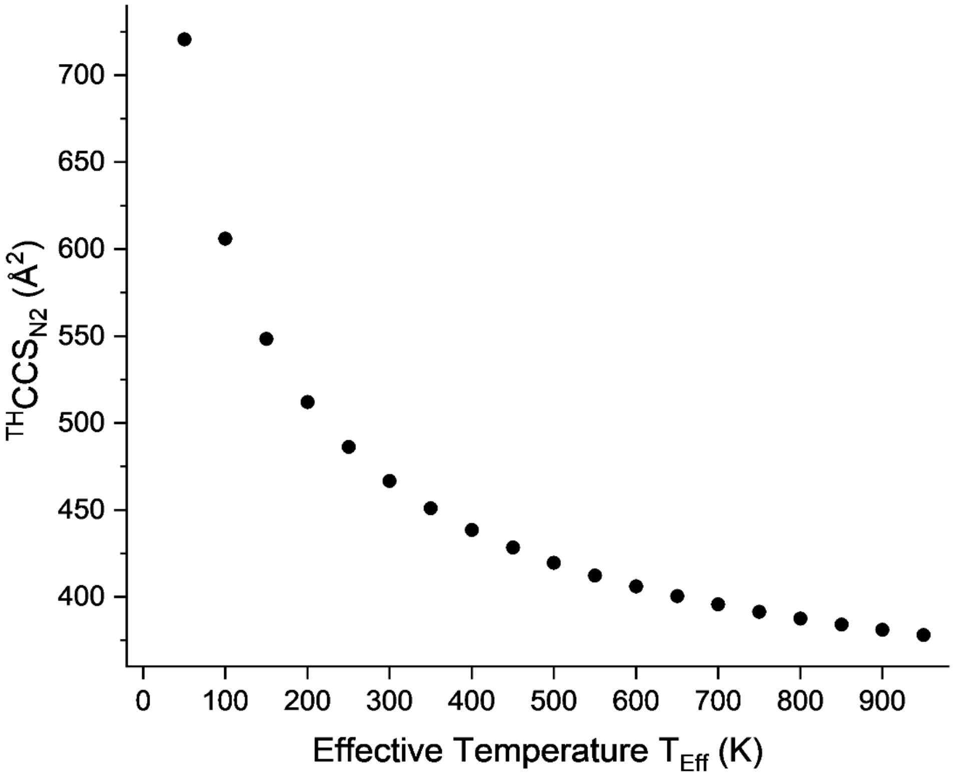

Figure S8: $^{TH}CCS_{N2}$ values of [**Ring$_{Ni}$**]$^-$ for different effective temperatures (Trajectory Method, IMoS).[3]

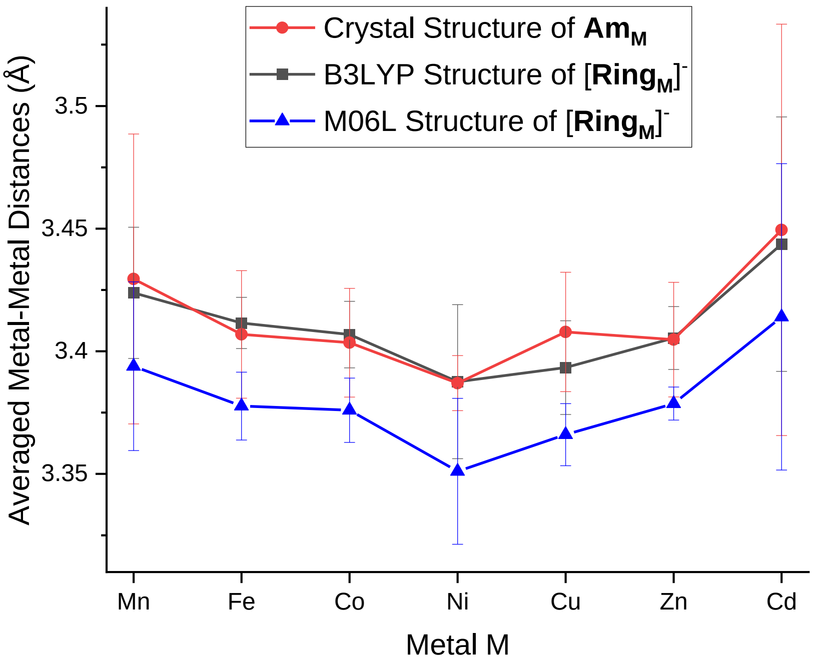

Figure S9: Averaged metal-metal distances for [**Ring$_M$**]$^-$ including error. Values were obtained from B3LYP and M06L optimised structures and as well as from unoptimised **Am$_M$** crystal structures.[2]

Discussion on differences between $^{TW}CCS_{N2}$ and $^{TH}CCS_{N2}$ for [Ring$_M$]$^-$

There is a systematic discrepancy between predicted and experimental CCS_{N2} values for [**Ring$_M$**]$^-$, with the experimental data being smaller in each case. This might be attributed to highly compact gas phase ions, but we consider this to be unlikely since the rings are highly stable in solution and were transferred without further activation into the gas phase.[4] Repulsion forces between the sterically demanding pivalate ligands also cause the [**Ring$_M$**]$^-$ to be kinetically trapped, which makes a spontaneous conversion to contracted conformations unlikely. Additional evidence is that we obtain similar results for both candidate [**Ring$_{Mn}$**]$^-$ conformers (Figures S3, S4), suggesting that these structural rearrangements will not significantly alter the CCS_{N2}. Opening the ring structure leads to higher CCS_{N2} values (Figures S6, S7), so this does not account for the difference either (Table S3).

The use of unsuitable basis sets/functionals for the DFT calculations also seems unlikely as the M06L and B3LYP optimised structure of [**Ring$_{Ni}$**]$^-$ yield highly similar $^{TH}CCS_{N2}$ values. The $^{TH}CCS_{N2}$ of the unoptimised crystal structure of **Am$_{Ni}$**[2] (only [**Ring$_{Ni}$**]$^-$ part was used) was ≈ 1% smaller than the $^{TH}CCS_{N2}$ of the B3LYP optimised structure, suggesting only a minor influence

of the performed geometry optimisations. We also compared the averaged metal-metal distances of the B3LYP and M06L optimised [$\mathbf{Ring_M}$]$^-$ structures with the unoptimised geometries obtained from the crystal structures of $\mathbf{Am_M}$,[2] showing a high agreement between crystal structure and B3LYP optimisation (Figure S9).

The impact of the buffer gas temperature on $^{TH}CCS_{N2}$ is known[5,6] and was simulated for [$\mathbf{Ring_{Ni}}$]$^-$ between effective temperatures of $T_{Eff} = 50 – 950\ K$ (Figure S8). Similar $^{TH}CCS_{N2}$ values than the experiment were obtained at temperatures of $T_{Eff} \approx 400\ K$, which could potentially occur due to ion heating upon injection in the travelling wave ion mobility cell. However, increasing the helium pressure did not show any effect, although is known to lower *CCS* at high temperatures.

The applied *CCS* prediction method, the trajectory method (TM), is considered the gold-standard for that purpose, but was developed, refined and parameterised with biomolecules like peptides and proteins.[7,8] Systematic differences to experimental data were frequently observed for metallosupramolecular compounds, in particular when assemblies with cavities were considered.[9–13] We believe that adapting the TM method for metallosupramolecular assemblies could improve the theoretical results.

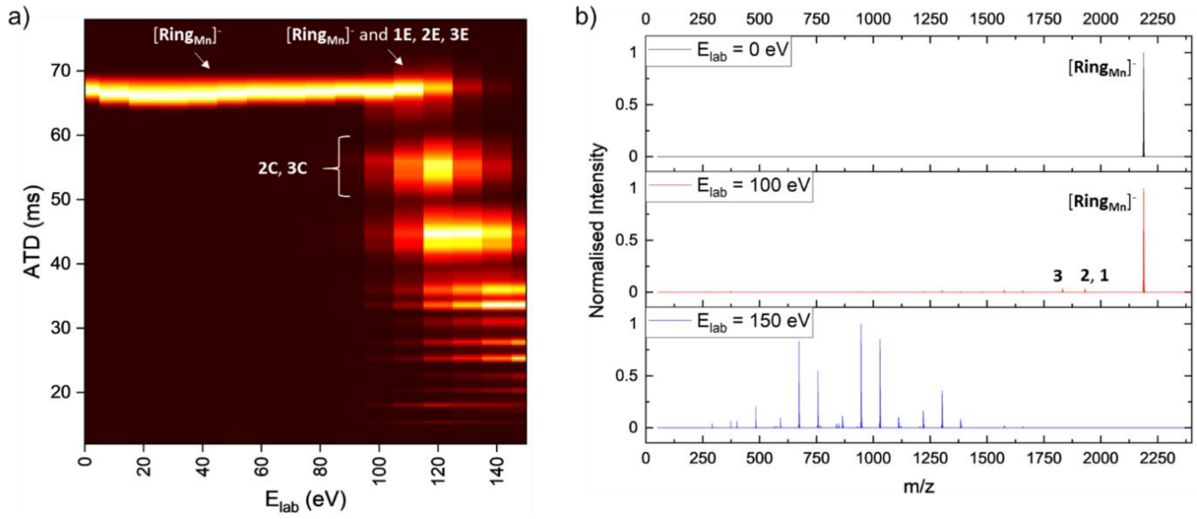

Figure S10: a) Collision Energy vs. ATD heat map for [**Ring$_{Mn}$**]$^-$ and its fragments. b) Corresponding MS^2 spectra at selected E_{lab}. 10 µM [$NH_2(CH_3)_2$][**Ring$_{Mn}$**] were used in 500 µM NaI and 4:1 toluene/methanol and [**Ring$_{Mn}$**]$^-$ was isolated at *m/z* = 2188.

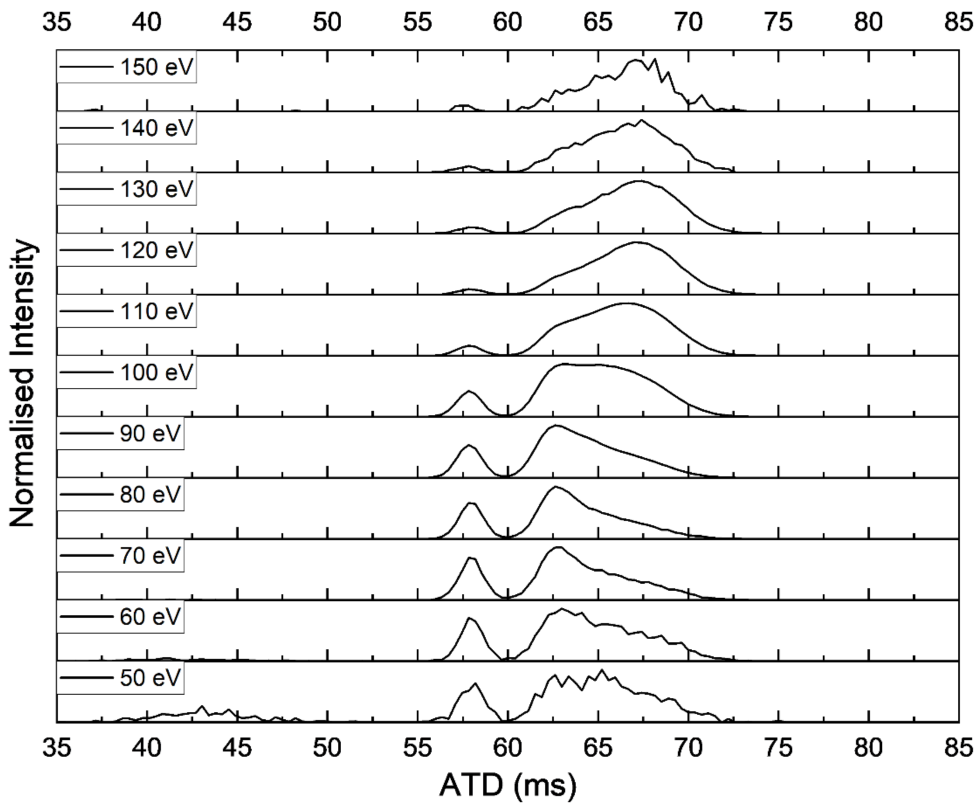

Figure S11: Stacked ATD plot for **1** at E_{lab} = 50 – 150 eV following activation of [**Ring$_{Cu}$**]$^{-}$ (m/z = 2197).

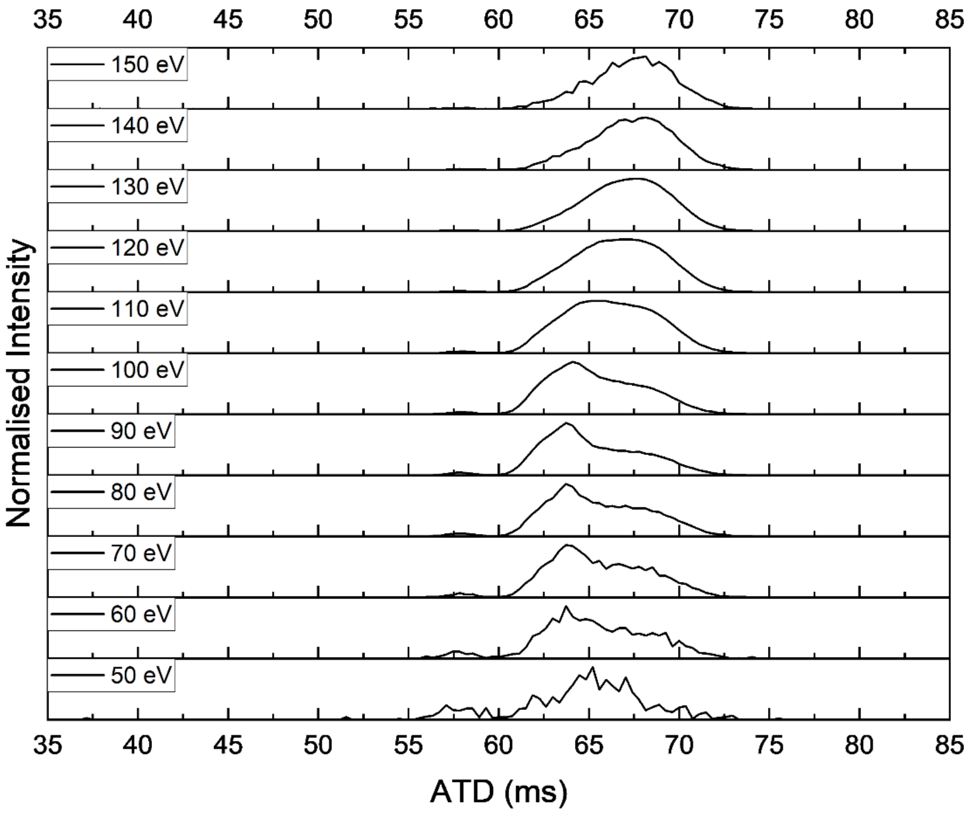

Figure S12: Stacked ATD plot for **1** at E_{lab} = 50 – 150 eV following activation of [**Ring$_{Mn}$**]$^-$ (*m/z* = 2188).

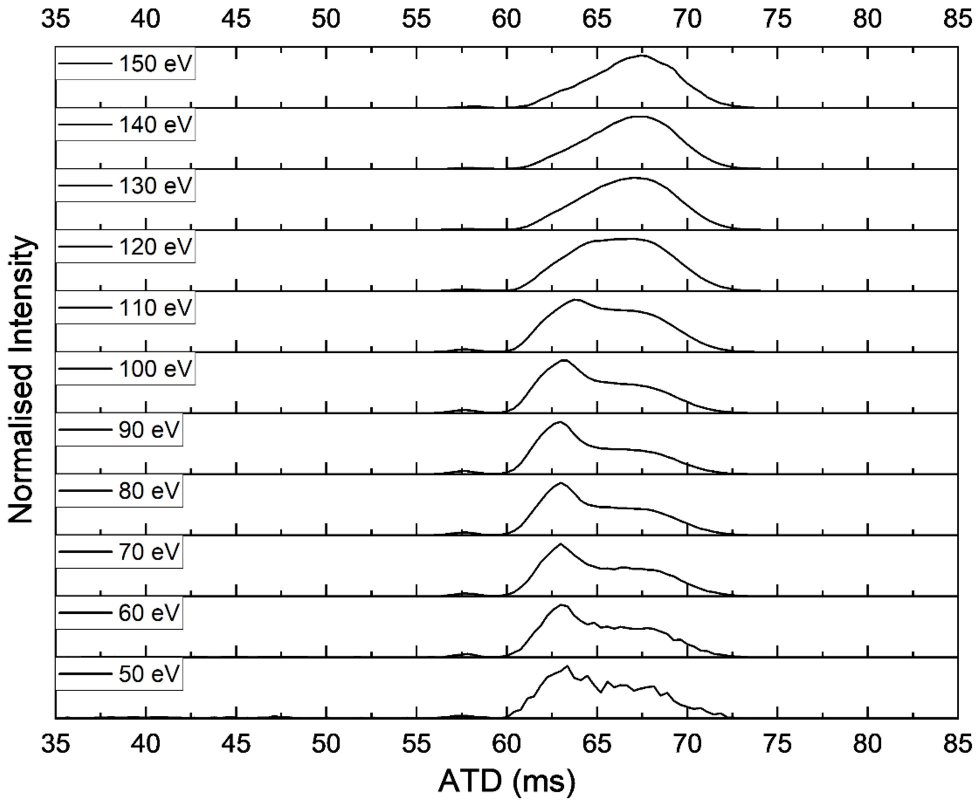

Figure S13: Stacked ATD plot for **1** at E_{lab} = 50 – 150 eV following activation of [**Ring**$_{\mathbf{Ni}}$]$^-$ (*m/z* = 2192).

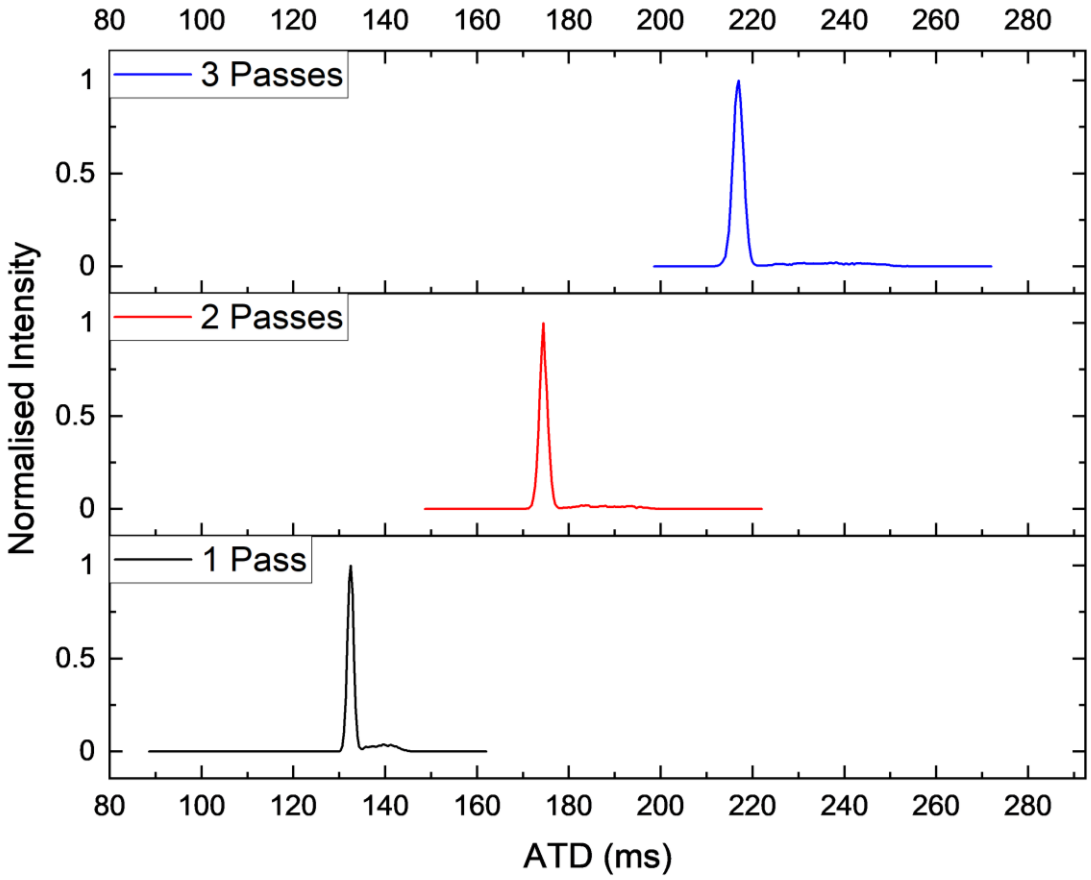

Figure S14: IMS[2] separation of **3C** at E_{lab} = 110 eV (Figure 3b). Separation was performed over 1-3 passes in the Cyclic drift ring. 200 µM $[NH_2(CH_3)_2]$[**Ring$_{Mn}$**] was used in 500 µM NaI and 4:1 toluene/methanol. The small, later arriving signal is likely due to imperfect selection and may correspond to **3E**.

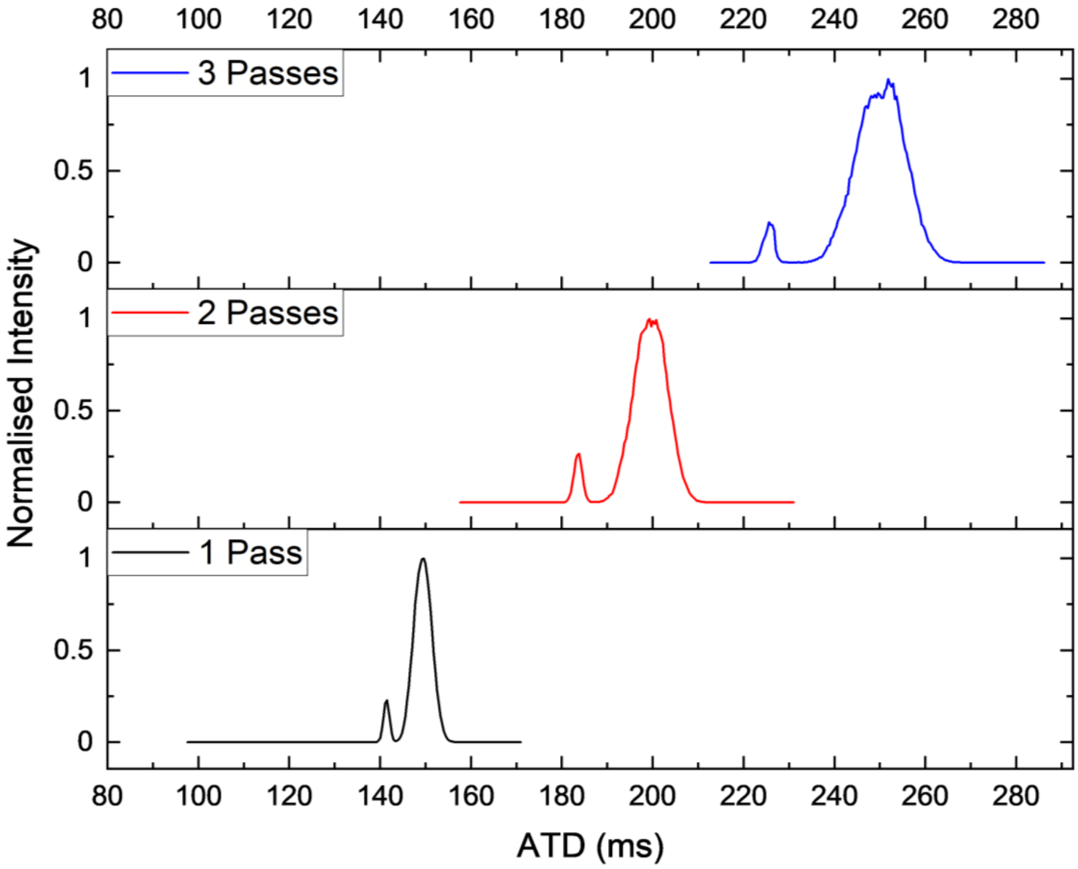

Figure S15: IMS^2 separation of **3E** at E_{lab} = 110 eV (Figure 3b). Separation was performed over 1-3 passes in the Cyclic drift ring. 200 µM [$NH_2(CH_3)_2$][**Ring$_{Mn}$**] was used in 500 µM NaI and 4:1 toluene/methanol. The appearance of **3C** in the IMS^2 spectra of **3E** may be attributed to metastable fragmentation from the parent ion, which is mobility coincident with the selected fragment.

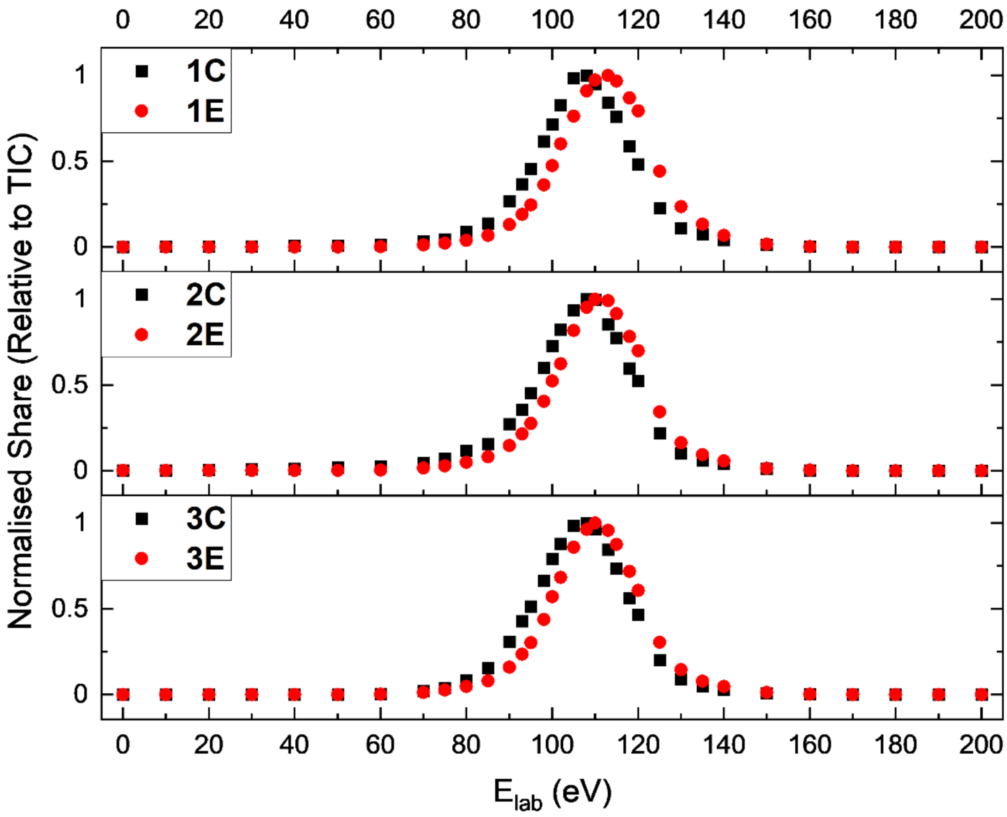

Figure S16: Share of **C** (black) and **E** (red) of fragment ions **1**-**3** in dependence of E_{lab} applied to the precursor [**Ring$_{Mn}$**]$^-$. In this analysis, we compare the share of the *m/z*- and ion mobility selected peaks relative to the total ion count at each E_{lab}. 200 µM $[NH_2(CH_3)_2]$[**Ring$_{Mn}$**] was used in 500 µM NaI and 4:1 toluene/methanol.

Table S4: Predicted $^{TH}CCS_{N2}$ values of different candidate structures for **3** including error. $^{TH}CCS_{N2}$ values were scaled with 0.924 (± 0.002) derived from the averaged ratio of $^{TW}CCS_{N2}/^{TH}CCS_{N2}$ of each [**Ring$_M$**]$^-$ (Table S2). The nomenclature was used as described for Figure S5.

**Structure**	**Nomenclature**	**Unscaled** TH**CCS$_{N2}$ in Å²**	**Scaled** TH**CCS$_{N2(s)}$ in Å²**
Closed Ring **3$_I$**	e2-e2-e2-e2-e2-e2	425.2 ± 0.8	392.9 ± 1.6
Open **3$_{II}$**	e2-e2-e2-e2-e2-a3	435.1 ± 1.8	402.0 ± 2.5
Open **3$_{III}$**	e2-e2-e2-e2-a3-e2	438.4 ± 1.2	405.1 ± 2.0
Open **3$_{IV}$**	e2-e2-e2-a3-e2-e2	430.5 ± 1.1	397.8 ± 1.9
Open **3$_V$**	e2-a3-e2-a3-e2-a3	445.1 ± 1.9	411.3 ± 2.6
Open **3$_{trans}$**	all adjacent F trans	442.7 ± 1.8	409.1 ± 2.5

As discussed in the main text, the scaled $^{TH}CCS_{N2(s)}$ of the closed ring **3$_I$** and open **3$_{IV}$** agree well with the experimental value of **3C**, indicating that the scaling factor has merit for comparing the obtained experimental and theoretical CCS_{N2} values. However after an extensive conformational search for the opened structures, including structures not presented here, we were only able to find candidate geometries that either agreed unscaled with the higher end of the experimental $^{TW}CCS_{N2}$ of **3E**, or in a scaled form with the lower end of **3E** (Table S4, Figure 1b). These results warrant further investigation on the use of the trajectory method for metallosupramolecular assemblies involving cavities, for which gas collisions inside the cavity might not be accounted for correctly.

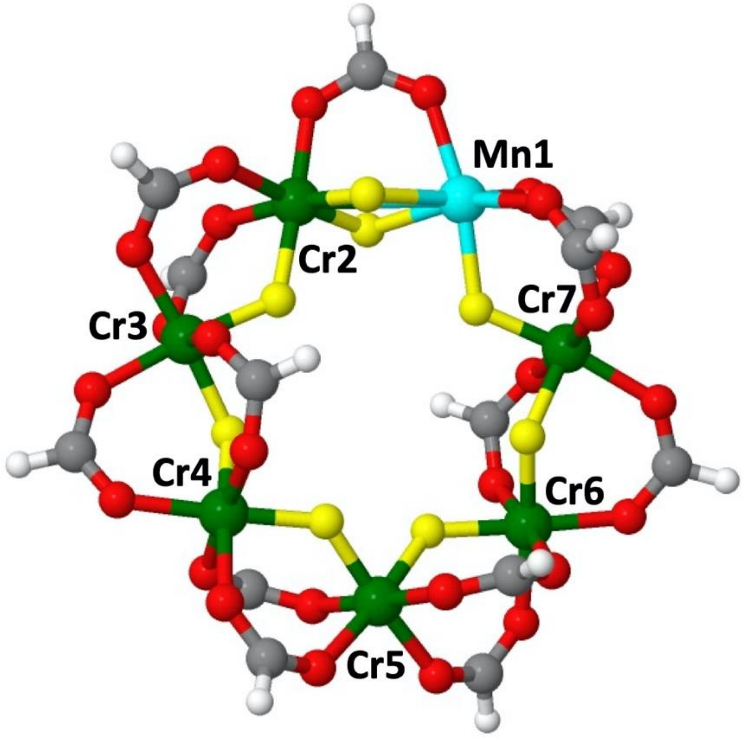

Figure S17: DFT optimised structure of closed ring **3$_{I}$** (Mn: cyan, Cr: green, F: yellow, O: red, C: grey, H: white). *Tert*-butyl groups were replaced with hydrogen for clarity. Optimized coordinates, ESP charges and minimum energies can be found in the Supplementary Dataset.

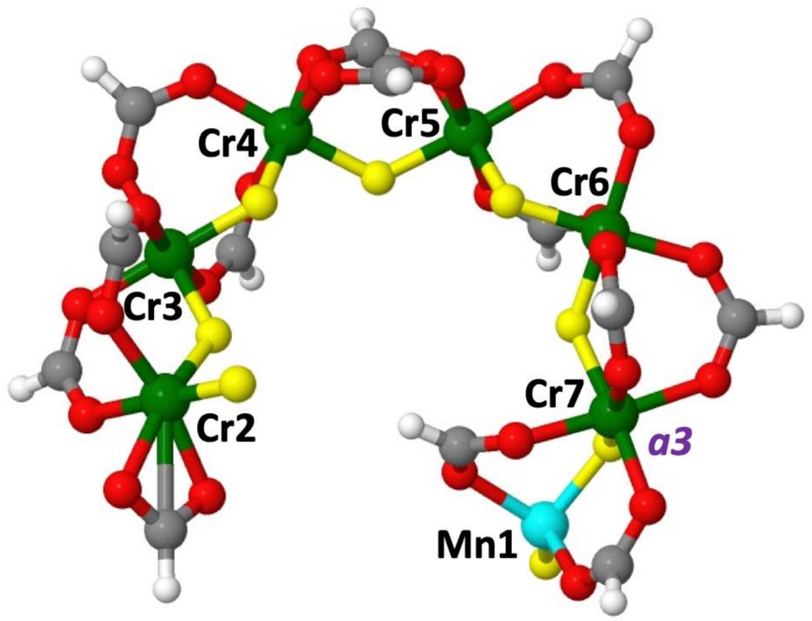

Figure S18: DFT optimised structure of open $\mathbf{3_{II}}$ (Mn: cyan, Cr: green, F: yellow, O: red, C: grey, H: white). *Tert*-butyl groups were replaced with hydrogen for clarity. Optimized coordinates, ESP charges and the minimum energy can be found in the Supplementary Dataset.

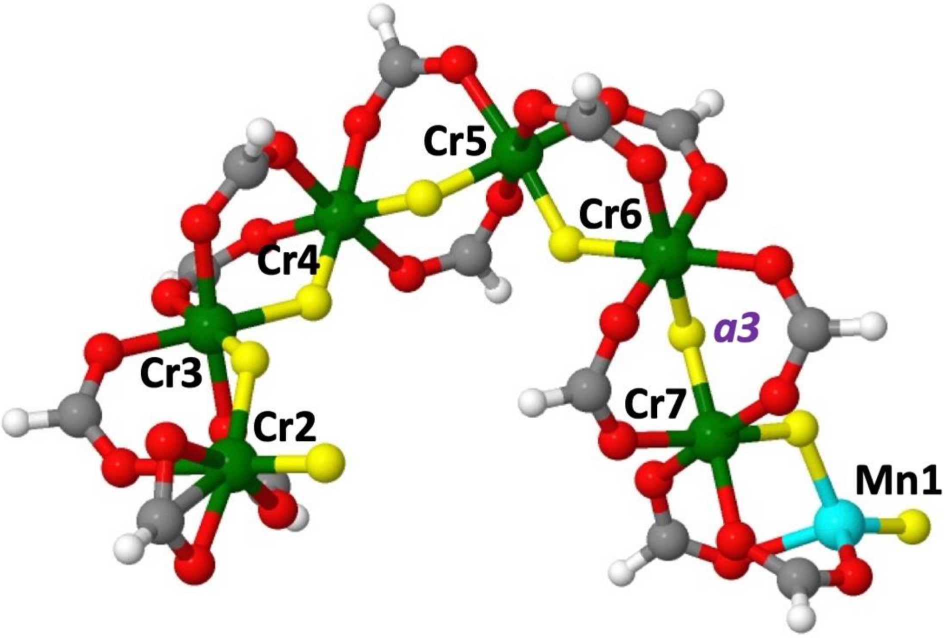

Figure S19: DFT optimised structure of open $\mathbf{3_{III}}$ (Mn: cyan, Cr: green, F: yellow, O: red, C: grey, H: white). *Tert*-butyl groups were replaced with hydrogen for clarity. Optimized coordinates, ESP charges and the minimum energy can be found in the Supplementary Dataset.

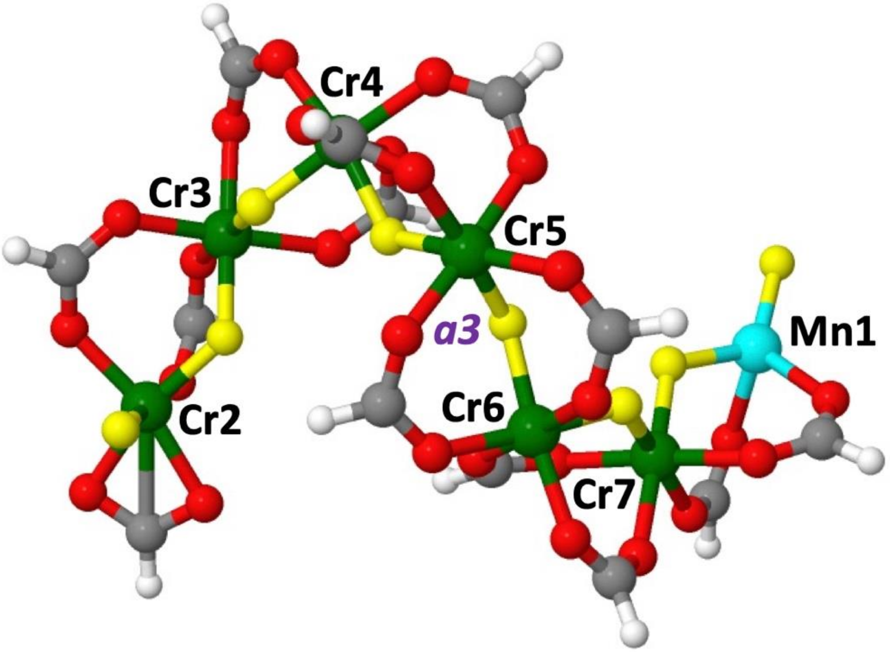

Figure S20: DFT optimised structure of open $\mathbf{3}_{IV}$ (Mn: cyan, Cr: green, F: yellow, O: red, C: grey, H: white). *Tert*-butyl groups were replaced with hydrogen for clarity. Optimized coordinates, ESP charges and the minimum energy can be found in the Supplementary Dataset.

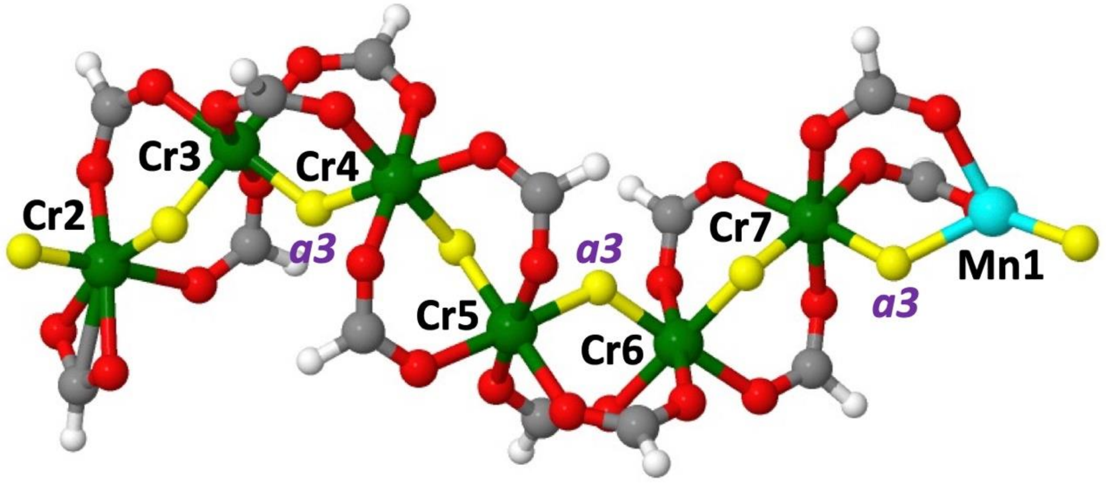

Figure S21: DFT optimised structure of open $\mathbf{3_V}$ (Mn: cyan, Cr: green, F: yellow, O: red, C: grey, H: white). *Tert*-butyl groups were replaced with hydrogen for clarity. Optimized coordinates, ESP charges and the minimum energy can be found in the Supplementary Dataset.

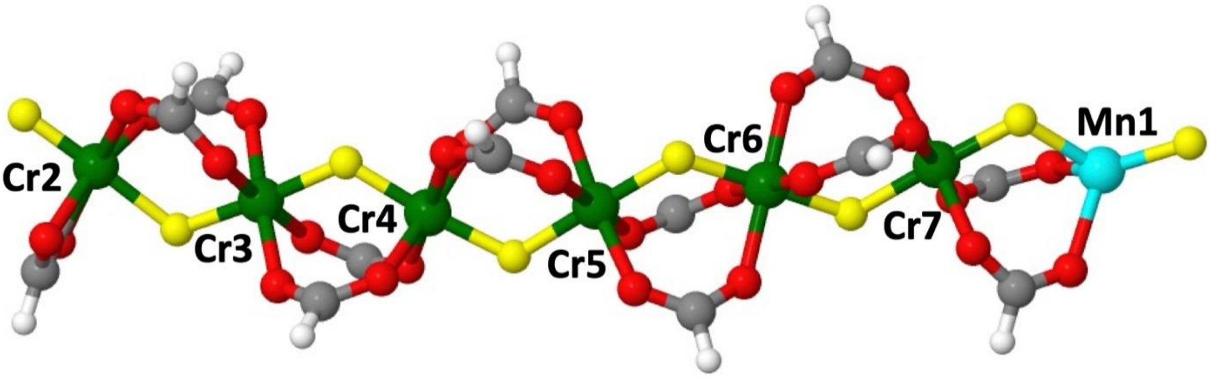

Figure S22: DFT optimised structure of open $\mathbf{3}_{trans}$ (Mn: cyan, Cr: green, F: yellow, O: red, C: grey, H: white). *Tert*-butyl groups were replaced with hydrogen for clarity. Optimized coordinates, ESP charges and the minimum energy can be found in the Supplementary Dataset.

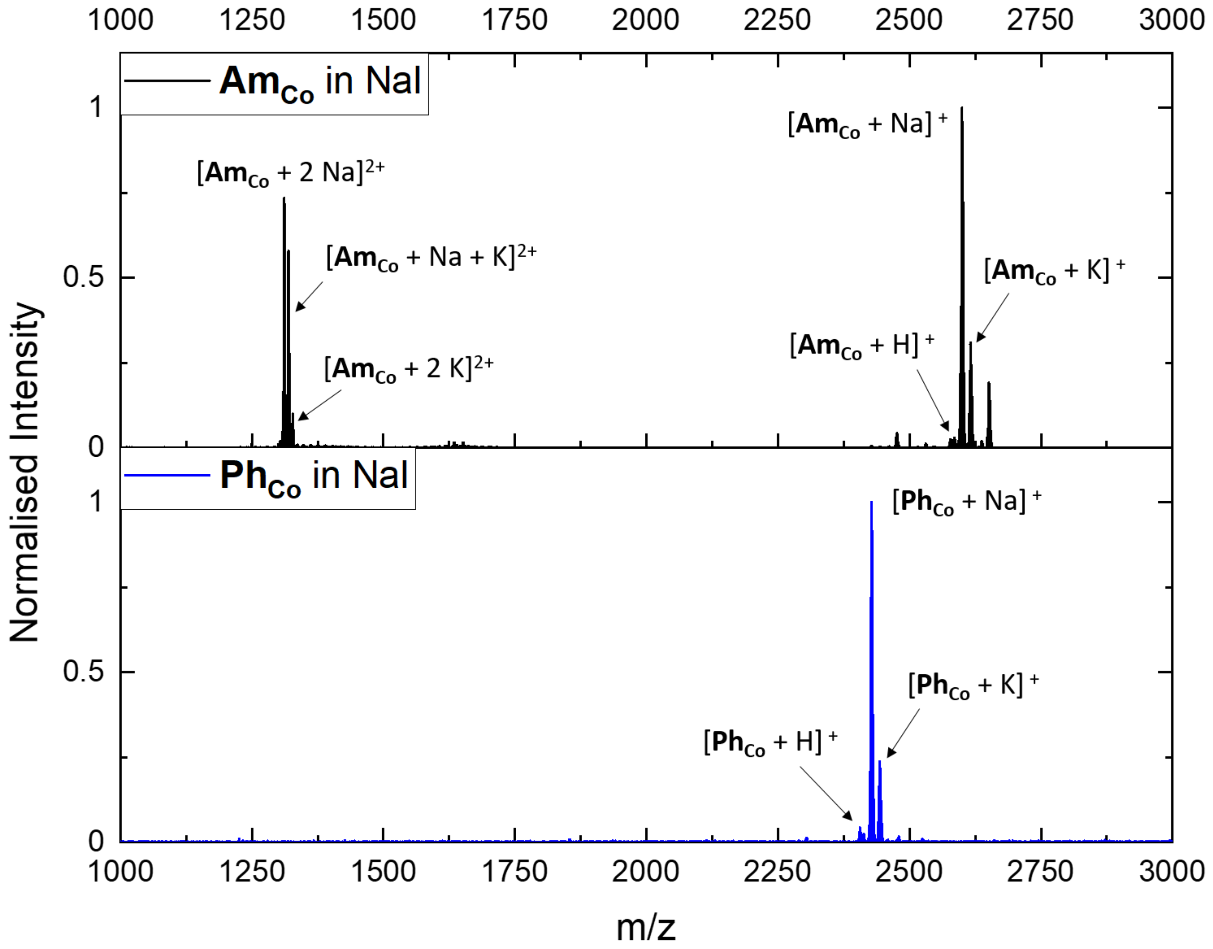

Figure S23: Mass spectra of **Ph$_{Co}$** and **Am$_{Co}$** in NaI. **Am$_{Co}$** in NaI (bottom) yielded dications of the formulas [**Am$_{Co}$** + A + A']$^{2+}$ ($A^+ = A'^+ = Na^+$; $A^+ = Na^+$, $A'^+ = K^+$), whereas almost no dications were observed for **Ph$_{Co}$** (top). The presence of these doubly-charged species is likely caused by the capability of **TAm$^+$** to bind cations *via* its amide end group, which is not possible for the phenyl end groups in **TPh$^+$**. 2 µM **Ph$_{Co}$** and **Am$_{Co}$** were used in 500 µM NaI and 4:1 toluene/methanol (**Ph$_{Co}$**) or 7:3 methanol/DCM (**Am$_{Co}$**).

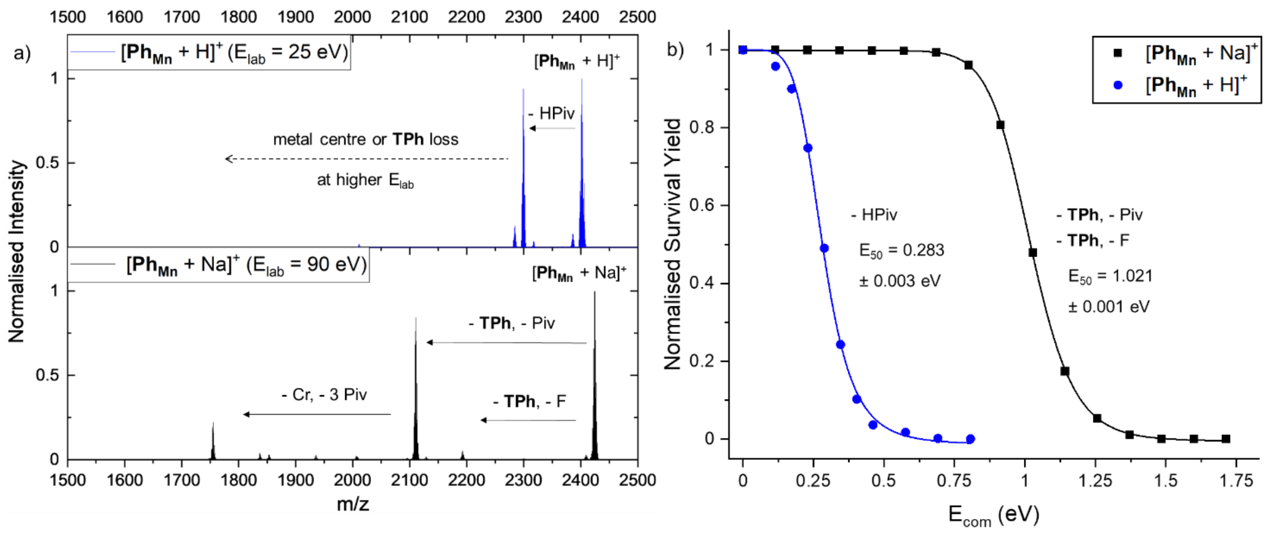

Figure S24: a) MS² spectra of [**Ph$_{Mn}$** + H]$^+$ at E_{lab} = 25 eV and [**Ph$_{Mn}$** + Na]$^+$ at E_{lab} = 90 eV. b) Normalised survival yield vs. E_{com} for [**Ph$_{Mn}$** + H]$^+$ and [**Ph$_{Mn}$** + Na]$^+$. The data was fitted with a sigmoidal Hill function. 2 µM **Ph$_{Mn}$** were used in 500 µM NaI and 4:1 toluene/methanol. [**Ph$_{Mn}$** + H]$^+$ was isolated at *m/z* = 2401 and [**Ph$_{Mn}$** + Na]$^+$ at *m/z* = 2423.

The fragmentation pathways are similar for both families **Ph$_M$** and **Am$_M$** and all seven different ring metals M, but are significantly different between protonated and sodiated forms. For [**Ph$_{Mn}$** + H]$^+$, the loss of one pivalic acid (HPiv, Figure S24a top) was observed at relatively low energies (Figure S24b). Further fragmentation steps include the loss of **TPh**$^+$ and an anionic ligand, before metal centres (Mn^{II}, Cr^{III}) are lost at even higher energies (Figure S25). The sodiated form [**Ph$_{Mn}$** + Na]$^+$ shows a different disassembly channel at higher energies, involving the loss of the thread **TPh**$^+$ and one anionic ligand (Piv or F, Figure S24). These primary fragments dissociate further and loose metal centres (Mn^{II}, Cr^{III}) in subsequent steps (Figures S24a bottom, S26). Differing fragmentation mechanisms for other rotaxane ions [**Ph$_M$** + A]$^+$ and [**Am$_M$** + A]$^+$ (A^+ = H^+, Na^+) are shown and discussed below (Figures S27 - S29).

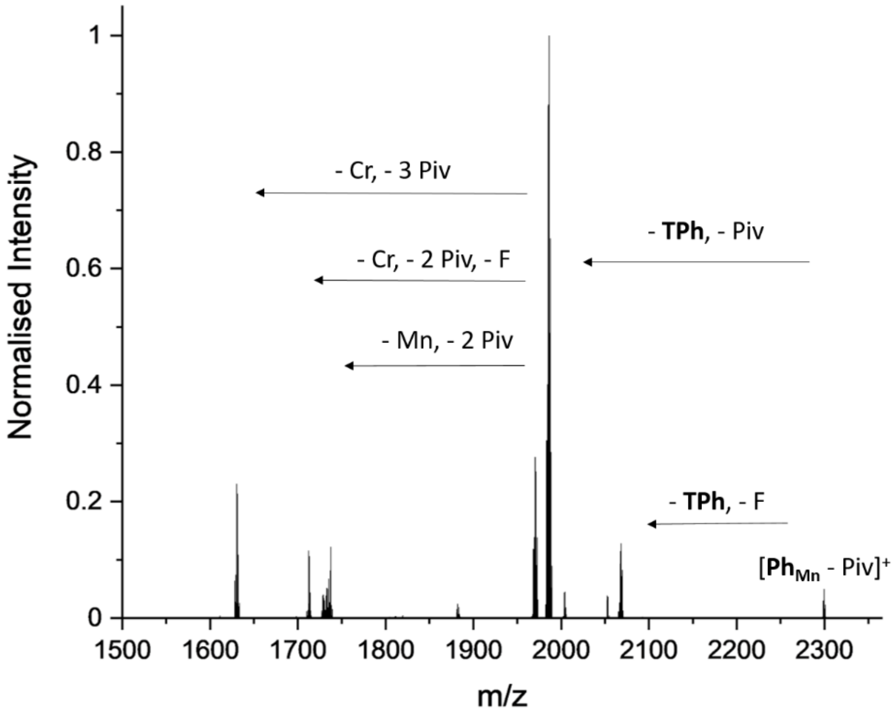

Figure S25: MS^2 spectrum of [**Ph$_{Mn}$** + H]$^+$ at E_{lab} = 110 eV. 2 µM **Ph$_{Mn}$** were used in 500 µM NaI and 4:1 toluene/methanol. [**Ph$_{Mn}$** + H]$^+$ was isolated at *m/z* = 2401.

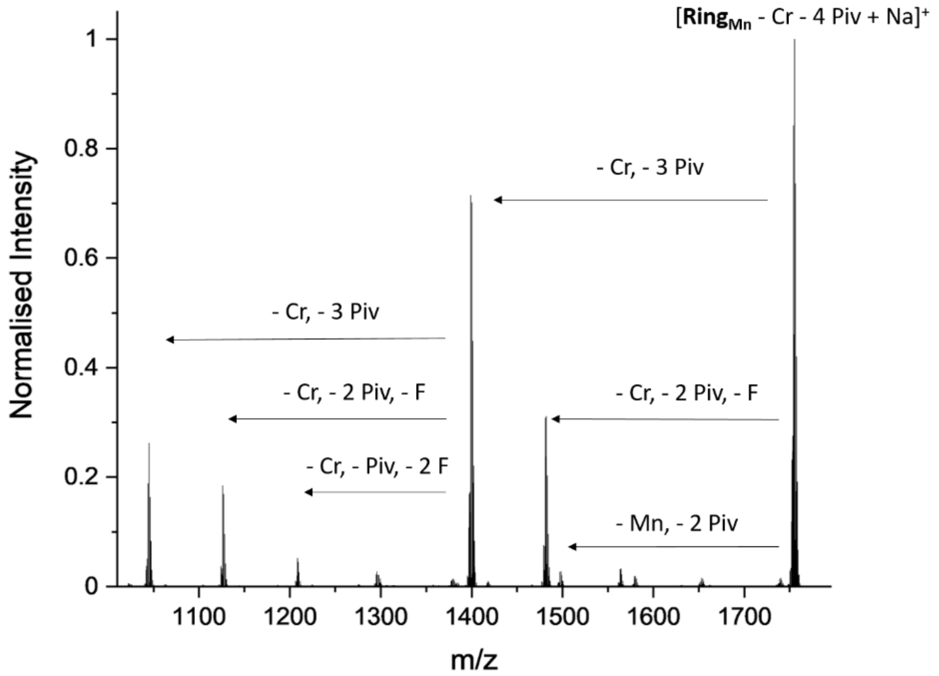

Figure S26: MS^2 spectrum of [**Ph$_{Mn}$** + Na]$^+$ at E_{lab} = 150 eV. 2 µM **Ph$_{Mn}$** were used in 500 µM NaI and 4:1 toluene/methanol. [**Ph$_{Mn}$** + Na]$^+$ was isolated at *m/z* = 2423.

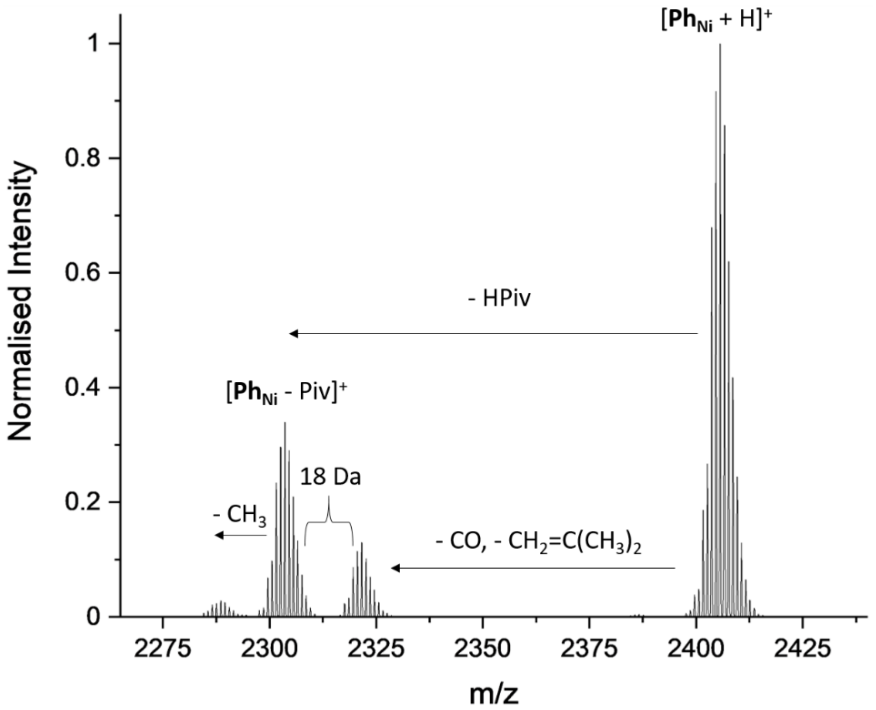

Figure S27: MS^2 spectrum of [**Ph_{Ni}** + H]$^+$ at E_{lab} = 25 eV. 2 µM **Ph_{Ni}** were used in 500 µM NaI and 4:1 toluene/methanol. [**Ph_{Ni}** + H]$^+$ was isolated at *m/z* = 2405. The two competing fragmentation pathways (loss of pivalic acid and loss of CO + isobutene) are illustrated.

Some ions of the type [**Ph_M** + H]$^+$ show another minor fragmentation channel besides the loss of one pivalic acid molecule. As shown in Figure S27 for [**Ph_{Ni}** + H]$^+$, we observed species of the type [**Ph_M**+ H - CO - CH_2=C(CH_3)$_2$]$^+$ (*m/z* = 2321 for M = Ni^{II}). This pathway was observed for the protonated **Ph_M** species with M = Mn^{II} (Figure S24a, *m/z* = 2317), Co^{II}, Ni^{II} and Cd^{II}, with Ni^{II} (Figure S27) showing by far the most intense peak.

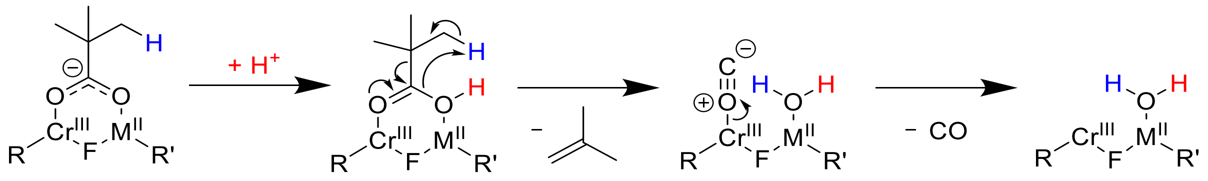

Figure S28: Suggested rearrangement mechanism for [$\mathbf{Ph_M}$ + H]$^+$. Isobutene and CO are lost and M remains with a water ligand. The intermediate with CO and H_2O bound was not found experimentally, but is shown for clarity.

The loss of CO and $CH_2{=}C(CH_3)_2$, which occurs as a minor fragmentation channel for the ions [$\mathbf{Ph_M}$ + H]$^+$ (M = Mn^{II}, Co^{II}, Ni^{II}, Cd^{II}), can potentially be attributed to a rearrangement mechanism involving bond cleavage of the pivalate ligand and release of the neutral molecules carbon monoxide and isobutene, leaving the divalent metal with an attached water molecule instead of a protonated pivalate (Figure S28). The same dissociation pathway was previously found for the hydrogen bromide catalysed decomposition of pivalic acid, in which isobutene, carbon monoxide and water were quantitatively produced.[14] Interestingly, the preference of this pathway for [$\mathbf{Ring_{Ni}}$]$^-$ agrees with its catalytic activity towards olefin polymerization, which was shown to be significantly higher compared to other [$\mathbf{Ring_M}$]$^-$ anions.[15]

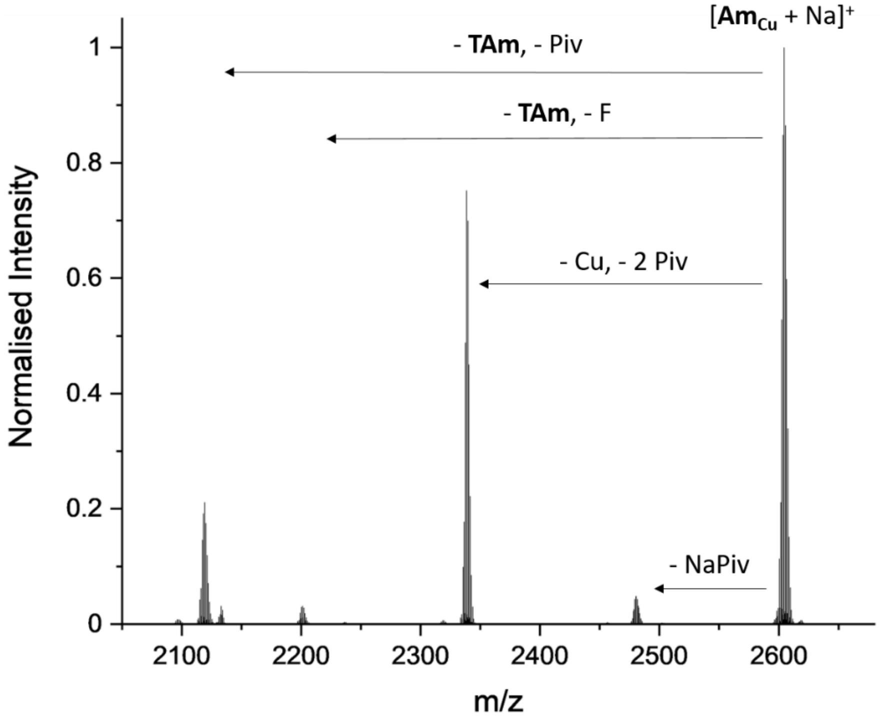

Figure S29: MS^2 spectrum of [$\mathbf{Am_{Cu}}$ + Na]$^+$ at E_{lab} = 100 eV. 10 µM $\mathbf{Am_{Cu}}$ were used in 500 µM NaI and 7:3 methanol/DCM. [$\mathbf{Am_{Cu}}$ + Na]$^+$ was isolated at *m/z* = 2605.

Alternative fragmentation channels are also observed for some of the sodiated forms [$\mathbf{Ph_M}$ + Na]$^+$ and [$\mathbf{Am_M}$ + Na]$^+$. One route is similar to the dissociation of the protonated species and involves the loss of NaPiv, which was observed as a minor pathway for all ions [$\mathbf{Am_M}$ + Na]$^+$ (Figure S29 for M = Cu^{II}) and even less pronounced for some of the ions [$\mathbf{Ph_M}$ + Na]$^+$. In general, the loss of one pivalic acid molecule seems to be energetically more favourable than the loss of sodium pivalate (NaPiv). This behaviour could be attributed to the ability of alkali metal cations to be chelated by multiple ligands, whereas protons are more likely only bound to a single basic site, in this case a pivalate. Alkali metals as bridging atoms are known from the literature[16–18] and would make the dissociation of NaPiv less favourable, whereas the higher charge density of the proton weakens the M-O bond and facilitates the dissociation of HPiv from the ring. Another reason could be that Na^+ preferentially binds to the thread, which would prevent the dissociation of NaPiv. This would involve the transfer of Na^+ to the ring during the collisionally induced thread loss, which we discuss further in another report.[19]

Another pathway appears for the alkali metal species [**Ph**$_{\mathbf{Cu}}$ + Na]$^+$ and [**Am**$_{\mathbf{M}}$ + Na]$^+$ (M = Cu^{II}, Zn^{II}, Cd^{II}) and shows the loss of the divalent metal centre and two pivalate ligands prior to the loss of the thread. This behaviour is strongly exhibited for [**Am**$_{\mathbf{Cu}}$ + Na]$^+$, where the loss of copper and two pivalate ligands is the main fragmentation channel (Figure S29). The preference of these two alternative fragmentation pathways for the ions [**Am**$_{\mathbf{M}}$ + Na]$^+$ can be explained with the higher energy demand of the competing main fragmentation channel compared to [**Ph**$_{\mathbf{M}}$ + A]$^+$ (Figure 2b and Table S1), which involves the loss of the bulky amide thread **TAm**$^{\mathbf{+}}$. Additionally, weaker M-O bonds support the dissociation of the divalent metal prior to thread loss, which is why only the sodiated species with the least stable [**Ring**$_{\mathbf{M}}$]$^-$ ions (M = Cu^{II}, Zn^{II}, Cd^{II}, Figure 1d) undergo this fragmentation channel. Both factors combined promote this disassembly pathway to the main fragmentation channel for [**Am**$_{\mathbf{Cu}}$ + Na]$^+$.

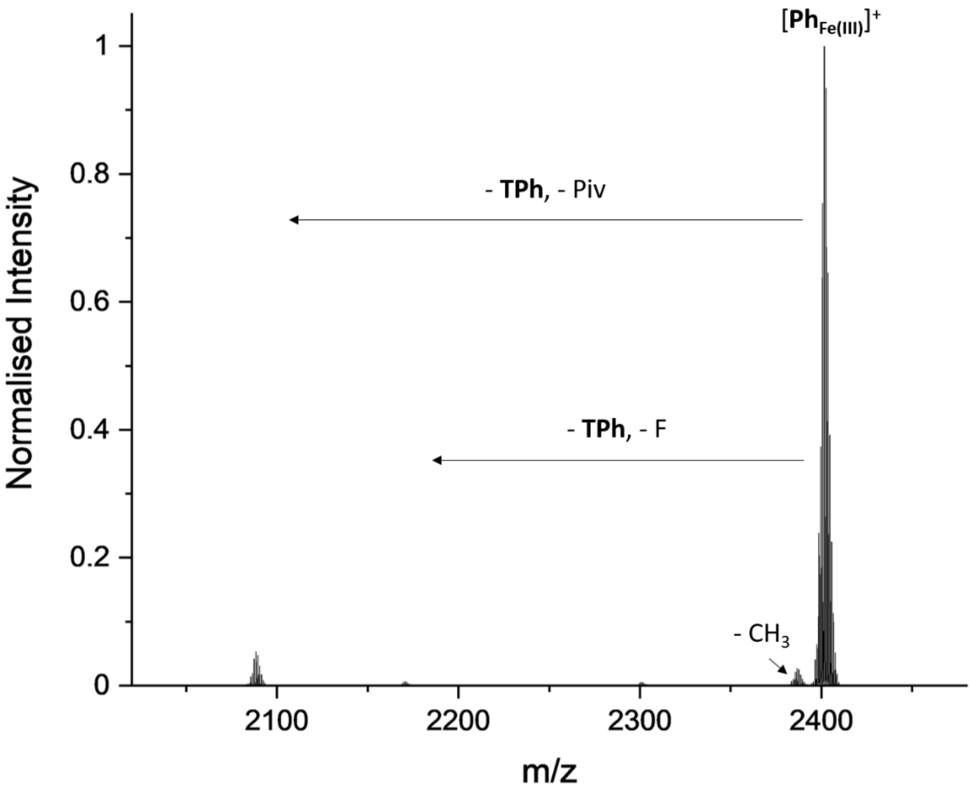

Figure S30: MS^2 spectrum of [$\mathbf{Ph}_{\mathbf{Fe(III)}}$]$^+$ at E_{lab} = 140 eV. 2 µM $\mathbf{Ph}_{\mathbf{Fe}}$ were used in 500 µM NaI and 4:1 toluene/methanol. [$\mathbf{Ph}_{\mathbf{Fe(III)}}$]$^+$ was isolated at *m/z* = 2401. [$\mathbf{Ph}_{\mathbf{Fe(III)}}$]$^+$ and the analogous [$\mathbf{Am}_{\mathbf{Fe(III)}}$]$^+$ each involve a Fe^{III} centre, but no additional cation A^+. The same fragmentation pathway was observed than for the sodiated forms containing M^{II}.

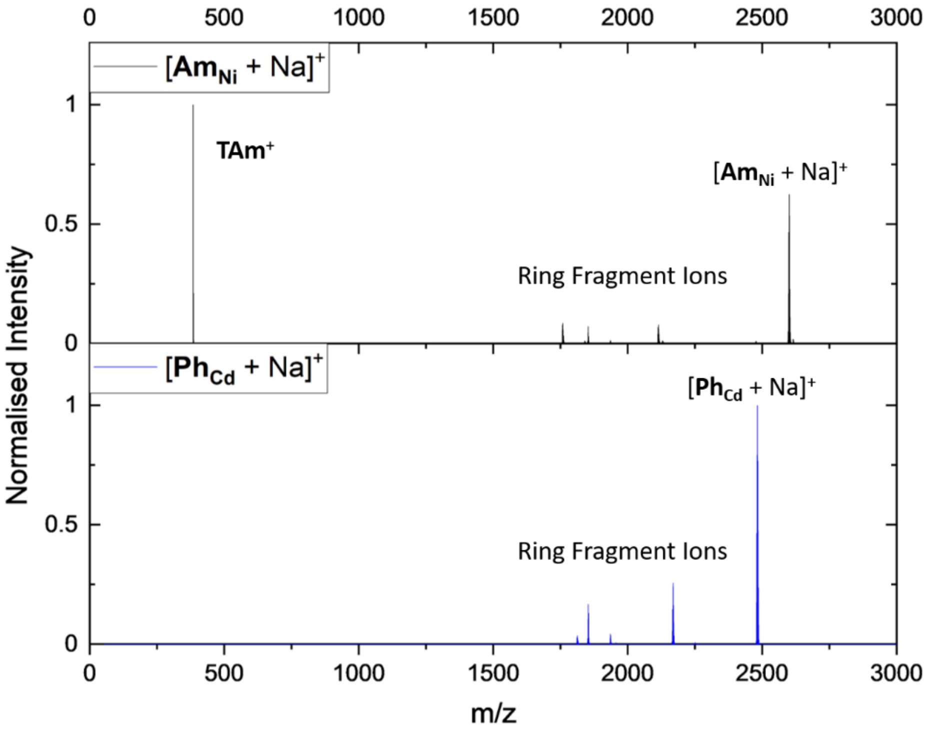

Figure S31: MS^2 spectrum of [**Am$_{Ni}$** + Na]$^+$ at E_{lab} = 110 eV and [**Ph$_{Cd}$** + Na]$^+$ at E_{lab} = 80 eV. 10 µM **Am$_{Ni}$**/**Ph$_{Cd}$** were used in 500 µM NaI and 7:3 methanol/DCM (**Am$_{Ni}$**) resp. 4:1 toluene/methanol (**Ph$_{Cd}$**). [**Am$_{Ni}$** + Na]$^+$ was isolated at *m/z* = 2599 and [**Ph$_{Cd}$** + Na]$^+$ at *m/z* = 2481. Although both spectra show ring fragment ions that do not contain the thread, only free **TAm**$^+$ ions were found at *m/z* = 384. No ions of **TPh**$^+$ (*m/z* = 212) or corresponding fragments were observed in notable amounts.

The absence of the intact ion **TPh**$^+$ and its fragments in the MS^2 spectra of all [**Ph$_M$** + A]$^+$ ions suggests a slightly different dissociation mechanism than for [**Am$_M$** + A]$^+$, where the thread cation **TAm**$^+$ is observed. A likely explanation is the loss of the thread as a neutral molecule [**TPh** – H], which cannot be detected with mass spectrometry. Unpublished data from other polymetallic rotaxanes suggests that a proton transfer from the thread to ring is possible, in which case the proton is possibly lost together with a pivalate ligand in form of a pivalic acid molecule. The thread **TAm**$^+$ might not follow this mechanism due to its amide end groups, holding stronger to the cation than the secondary amine unit in the centre.

Table S5: Water-exchange reaction rate constants k_{H2O} and pK_A values of hexaaqua ions $[M^{II}(H_2O)_6]^{2+}$. k_{H2O} ($T = 298\ K$) values for Mn^{II}, Fe^{II}, Co^{II}, Ni^{II} and Cu^{II} were experimentally obtained, whereas molecular dynamics simulations were used to derive the values for Zn^{II} and Cd^{II}. All pK_A values were experimentally determined.

**Metal M**	k_{H_2O} **in** s^{-1}	$\log(k_{H_2O})$	**Ref.** k_{H_2O}	pK_A **of** $[M(H_2O)_6]^{2+}$	**Ref.** pK_A
**Mn^{II}**	$2.1 \cdot 10^7$	7.3	[20]	10.6	[21]
**Fe^{II}**	$4.4 \cdot 10^6$	6.6	[20]	-	-
**Co^{II}**	$3.2 \cdot 10^6$	6.5	[20]	9.7	[21]
**Ni^{II}**	$3.2 \cdot 10^4$	4.5	[20]	9.86	[21]
**Cu^{II}**	$4.4 \cdot 10^9$	9.6	[22]	8.0	[21]
**Zn^{II}**	$4.1 \cdot 10^8$	8.6	[23]	9.0	[21]
**Cd^{II}**	$6.1 \cdot 10^8$	8.8	[23]	10.1	[24]

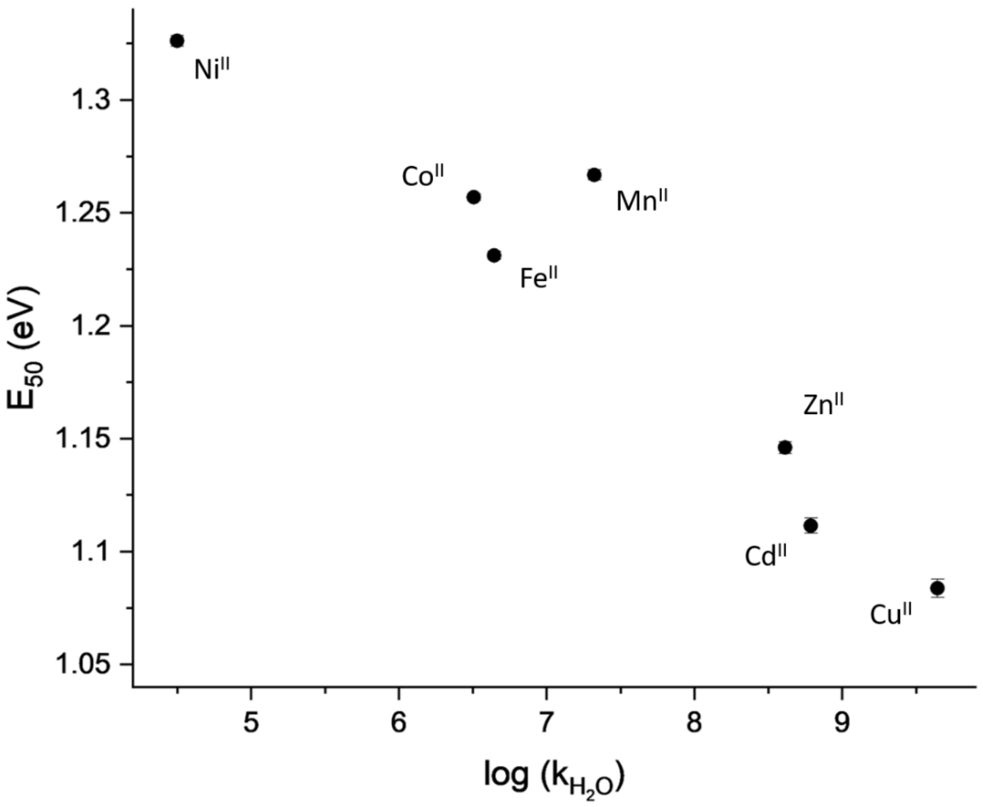

Figure S32: E_{50} values of [**Ring$_M$**]$^-$ vs. logarithm of the water-exchange reaction rate constants of $[M^{II}(H_2O)_6]^{2+}$. Values were used from Tables S1 and S5.

The hexaaqua ions $[M^{II}(H_2O)_6]^{2+}$ are suitable model compounds for the chemical environment of M^{II} in the heterometallic rings [**Ring$_M$**]$^-$ as both species involve M^{II} centres in an (almost) octahedral high-spin complex with at least four M^{II}-O bonds.[25,26] The water-exchange reaction rate constant of $[M^{II}(H_2O)_6]^{2+}$ quantifies the velocity with which water ligands of the first coordination sphere are exchanged for molecules of the second layer. This means that k_{H_2O} and $\log(k_{H_2O})$ can be interpreted as a measure of kinetic lability with the highest k_{H_2O} representing the least stable species. The plot above shows a good correlation with the most labile hexaaqua ion and heterometallic ring involving Cu^{II}, whereas the Ni^{II} species are most stable in both cases.

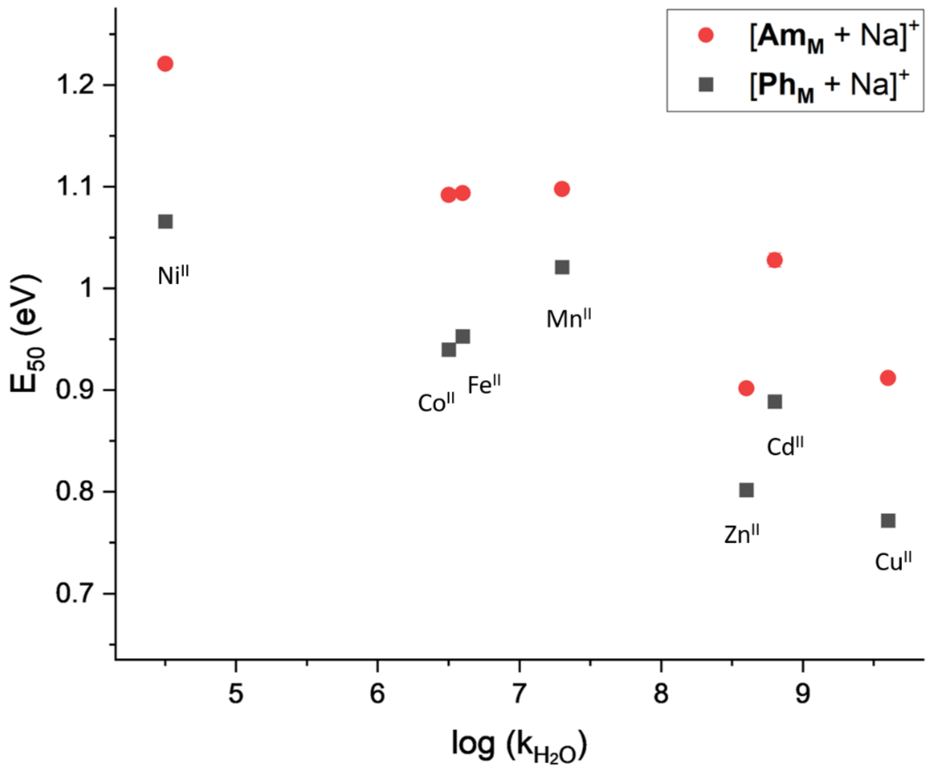

Figure S33: E_{50} values of [$\mathbf{Ph_M}$ + Na]$^+$ and [$\mathbf{Am_M}$ + Na]$^+$ vs. logarithm of the water-exchange reaction rate constants of $[M^{II}(H_2O)_6]^{2+}$. Values were used from Tables S1 and S5.

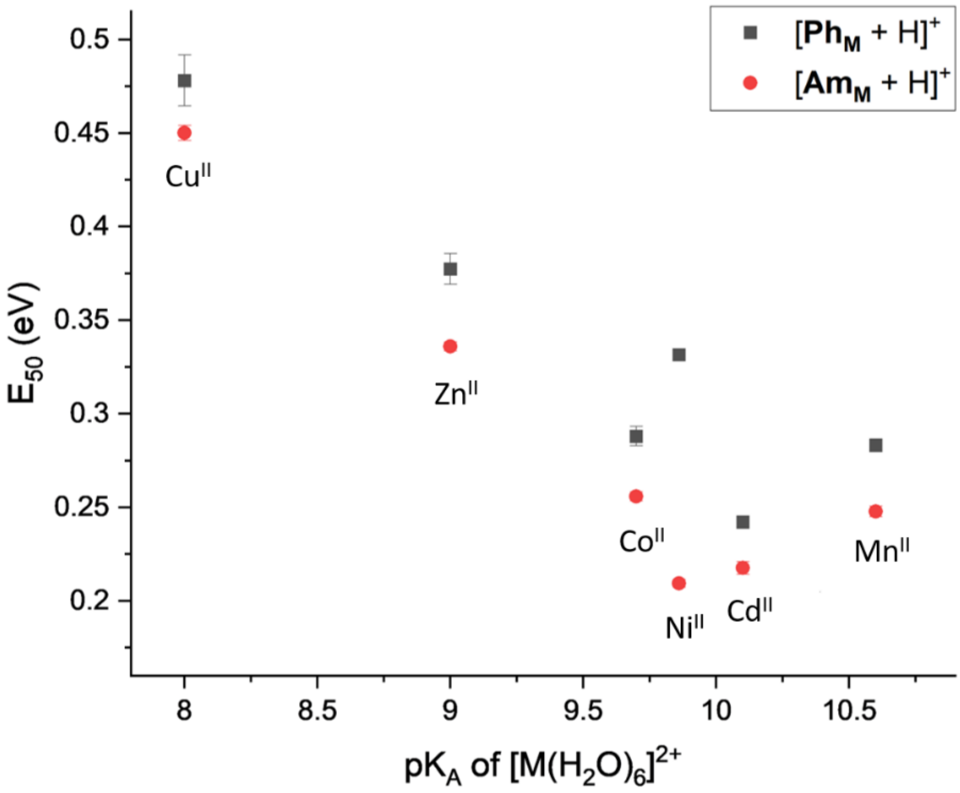

Figure S34: E_{50} values of [$\mathbf{Ph_M}$ + H]$^+$ and [$\mathbf{Am_M}$ + H]$^+$ vs. pK_A values of $[M^{II}(H_2O)_6]^{2+}$. Values were used from Tables S1 and S5.

The plot shows a strong linear correlation except for the ions with M = Mn^{II} and Ni^{II}. The species with M = Mn^{II} were observed to be outliers before (Figures 1d, 2b and S32), which is likely related to differences in the crystal structure of the [$\mathbf{Ring_{Mn}}$]$^-$, caused by a higher covalent radius of Mn.[1] The E_{50} values for the ions [$\mathbf{Ph_{Ni}}$ + H]$^+$ and [$\mathbf{Am_{Ni}}$ + H]$^+$ differ significantly stronger from each other compared to the other metals and in particular [$\mathbf{Ph_{Ni}}$ + H]$^+$ is an outlier in this correlation. A possible explanation is a distortion of the Ni^{II}-octahedron due to its $3d^8$ configuration, which seems to be strongly influenced by the choice of the thread.

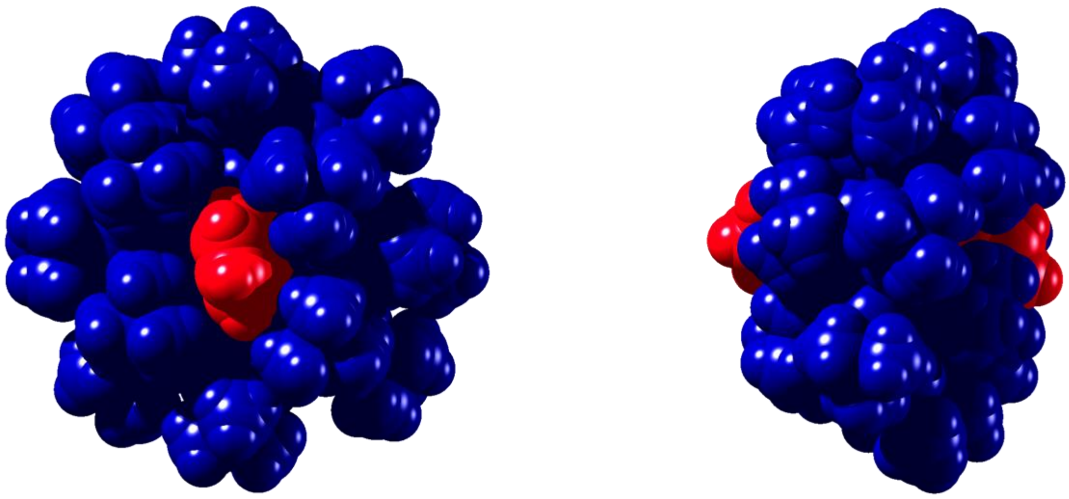

Figure S35: Space-filling model of $\mathbf{Ph_{Cd}}$ involving the heterometallic ring [$\mathbf{Ring_{Cd}}$]$^-$ (blue) and the thread $\mathbf{TPh^+}$ (red) in front view (left) and side view (right). Coordinates were obtained from the crystal structure (Table S7).

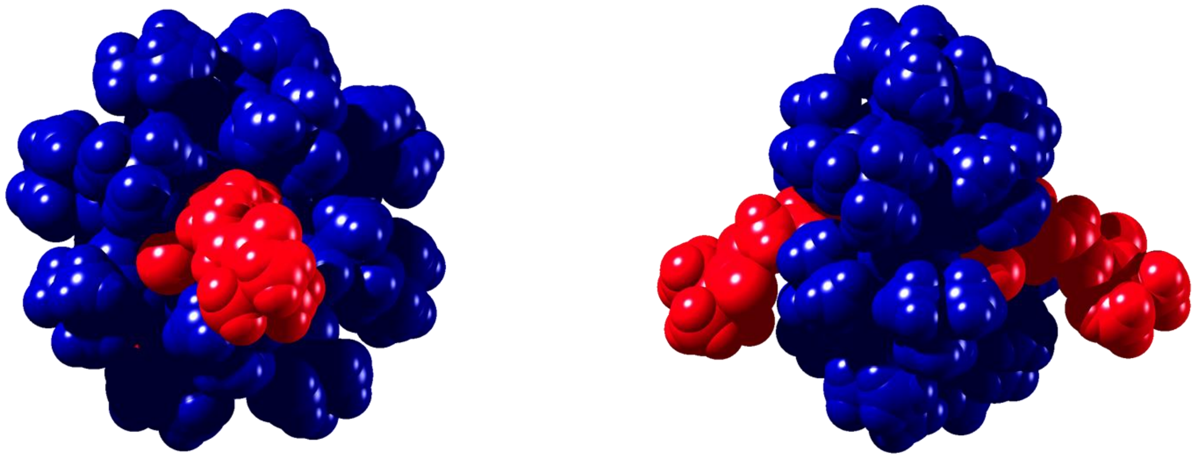

Figure S36: Space-filling model of **Am$_{Ni}$** involving the heterometallic ring [**Ring$_{Ni}$**]$^-$ (blue) and the thread **TAm**$^+$ (red) in front view (left) and side view (right). Coordinates were obtained from the corresponding crystal structure.[2]

The diameter of the [$\mathbf{Ring_M}$]$^-$ cavity can be estimated from the smallest 1,5-fluoride-fluoride distances in the crystal structure and the van der Waals radius of fluoride (r_{VdW} *(F)* = 1.47 Å) with Eq. S1.[27]

$$d_{Cavity} = d_{F-F} - 2 \cdot \mathrm{r_{VdW}}\,(F) \qquad \text{(Eq. S1)}$$

Similarly, the stopper width can be calculated by using the longest carbon-carbon distances in the stopper groups (**TPh**$^+$: 1,4-carbon-carbon distances in the phenyl end group, **TAm**$^+$: 1,3-carbon-carbon distances in the *tert*-butyl end group) and the van der Waals radius of carbon (r_{VdW} *(C)* = 1.70 Å, Eq. S2).[27]

$$d_{Stopper} = d_{C-C} + 2 \cdot \mathrm{r_{VdW}}\,(C) \qquad \text{(Eq. S2)}$$

The results of these calculations are presented in Table S6, showing that in both cases the stopper group is by far larger than the diameter of the ring cavity. Although these are only rough estimations, they indicate that any slipping mechanism must involve ring enlargement.

Table S6: Estimated cavity diameters and stopper widths of $\mathbf{Ph_{Cd}}$ and $\mathbf{Am_{Ni}}$ obtained from Eq. S1 and S2.

	d_{Cavity} in Å	$d_{Stopper}$ in Å
$\mathbf{Ph_{Cd}}$	3.53	5.78
$\mathbf{Am_{Ni}}$	3.48	5.94

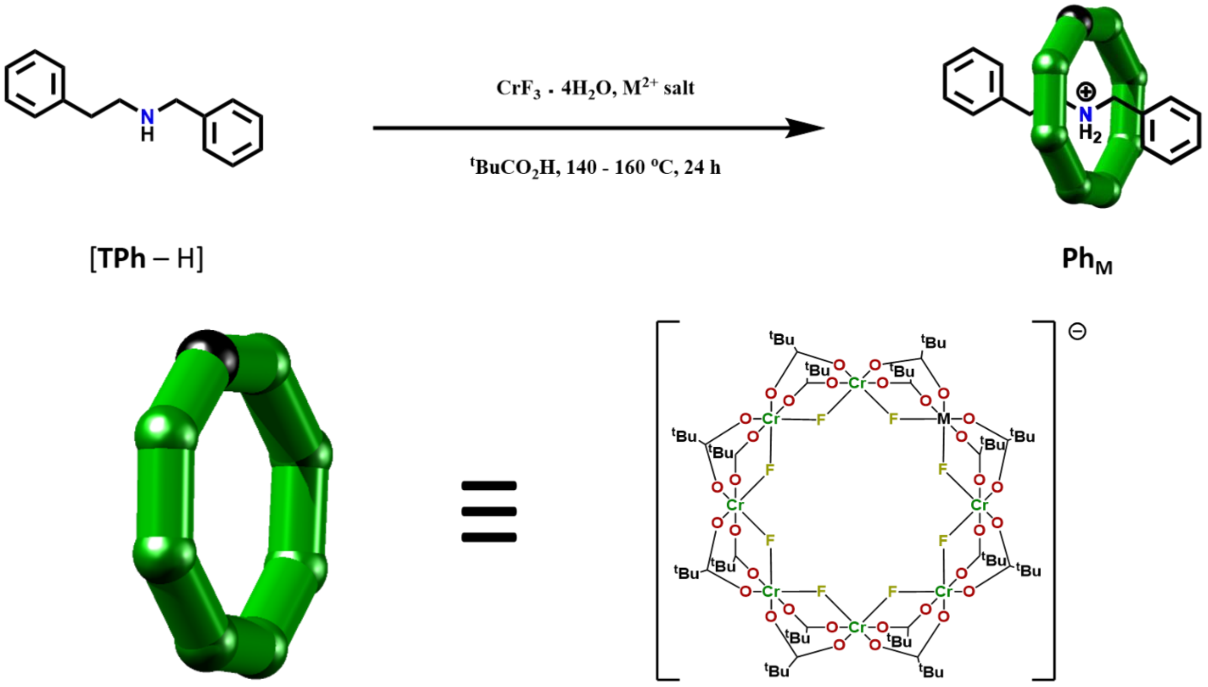

Figure S37: Synthetic procedure for rotaxane family **Ph$_M$** (M = Mn^{II}, Fe^{II}, Co^{II}, Ni^{II}, Cu^{II}, Zn^{II}, Cd^{II}).

N-benzyl-2-phenylethan-1-amine ([**TPh** – H])**:** Benzaldehyde (2.4 mL, 23.66 mmol) was dissolved in 200 mL of methanol and purged under a N_2 atmosphere for 1 h. Phenylethylamine (3.0 mL, 23.66 mmol) was added to the solution with stirring and refluxed for 5 h under an N_2 atmosphere before cooling to RT and left to stir overnight. $NaBH_4$ (4.48 g, 118.3 mmol) was added slowly to the solution before stirring overnight to reduce the imine. The reaction was quenched with 100 mL water and solvent was removed under a reduced pressure. The resultant solid was redissolved in 200 mL chloroform, washed with water (3 x 70 mL), dried over anhydrous magnesium sulphate and filtered. The solvent was removed under a reduced pressure yielding a colourless liquid (4.02 g, 80 %). ^{1}H-NMR δ ppm (500 MHz; $CDCl_3$): 2.75 (t, 2H), 2.83 (t, 2H), 3.72 (s, 2H), 7.10 – 7.25 (m, 10H). Positive ESI-MS (dissolved in THF, run in MeOH): *m/z* = 212.1 **TPh**$^+$.

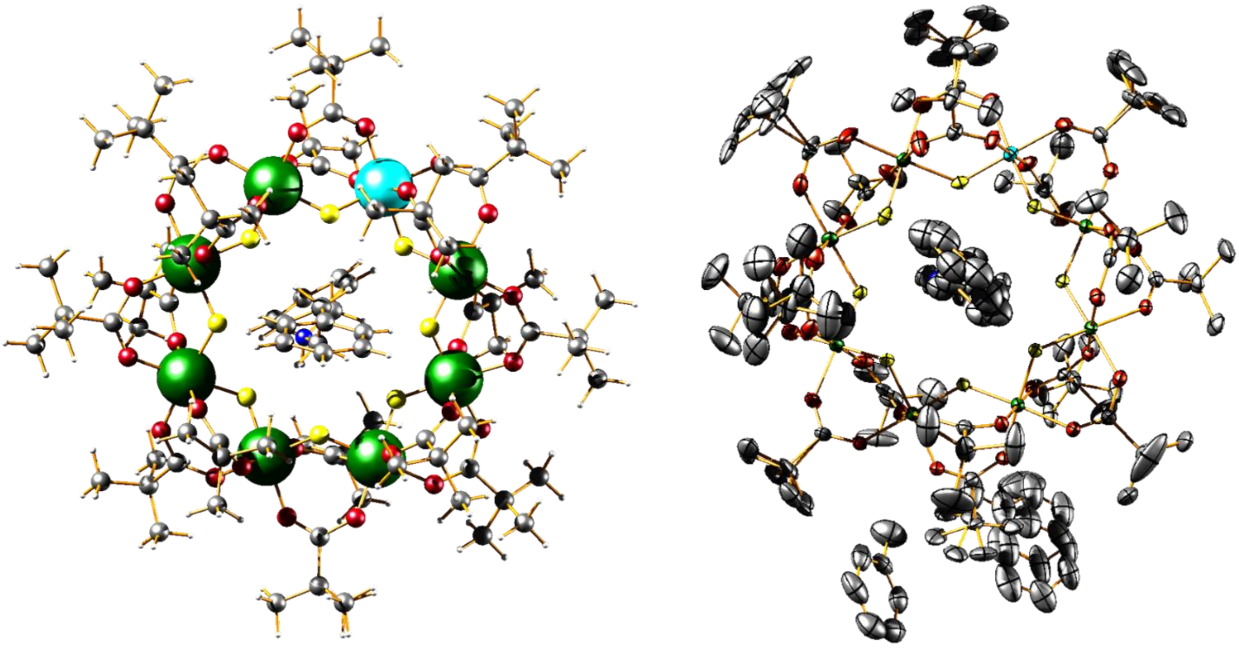

Figure S38: Single crystal X-ray structure of **Ph$_{Mn}$** (Mn: cyan, Cr: dark green, F: yellow, O: red, N: blue, C: grey, H: white) including the ORTEP diagram with the thermal ellipsoids drawn at 40% probability level (right). Solvent molecules in the asymmetric unit have been removed for clarity in the representation on the left. The Mn sites in the [**Ring$_{Mn}$**] unit are disordered over multiple positions.

$[NH_2(CH_2C_6H_5)(CH_2CH_2C_6H_5)][Cr_7Mn^{II}F_8(O_2C^tBu)_{16}]$ (Ph$_{Mn}$): N-benzyl-2-phenylethan-1-amine [**TPh** – H] (0.50 g, 2.37 mmol) was added to 15 g of pivalic acid in an open Teflon flask alongside $CrF_3 \cdot 4\ H_2O$ (3.00 g, 16.57 mmol) and heated to 140 °C. After 2 h, $MnCO_3$ (0.40 g, 3.48 mmol) was added to the reaction mixture and stirred at 140 °C for 24 h. Additional $MnCO_3$ (0.20 g, 1.74 mmol) was added to the reaction mixture before stirring the reaction mixture for a further 24 h in a N_2 atmosphere. The reaction was allowed to cool to RT before addition of acetonitrile (180 mL) precipitating a green solid which was collected by filtration. The product was extracted in toluene (100 mL) and solvent was removed under a reduced pressure. The crude product was purified by flash column chromatography (toluene: EtOAc, 50:1, v/v) to give a green crystalline solid (0.82 g, 14 %). Large, green, single crystals suitable for single crystal XRD were obtained following slow evaporation of toluene. Elemental analysis (%) calcd. for $C_{98}H_{168}Cr_7F_8NMnO_{33}$ (+ 1 acetone): C 47.86, H 6.89, N 0.57, Cr 14.80, Mn 2.23; found: C 47.96, H 6.87, N 0.59, Cr 14.35, Mn 1.96. Positive ESI-MS (dissolved in THF, run in MeOH): *m/z* = 2423.6 [**Ph$_{Mn}$** + Na]$^+$.

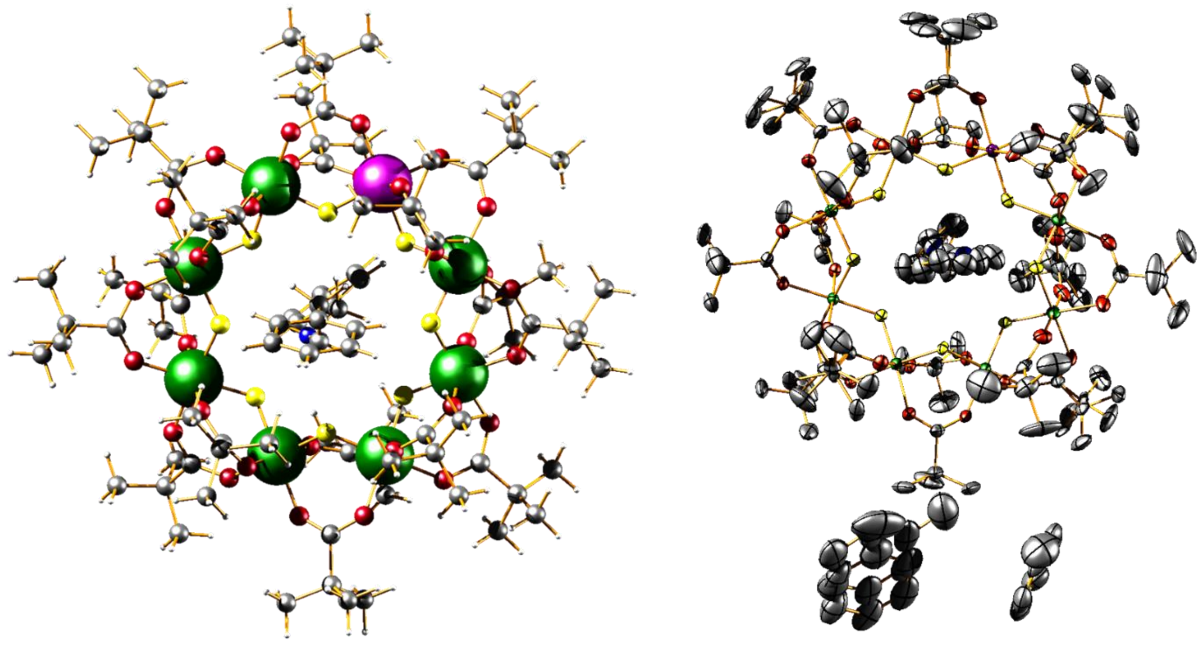

Figure S39: Single crystal X-ray structure of **Ph$_{Fe}$** (Fe: purple, Cr: dark green, F: yellow, O: red, N: blue, C: grey, H: white) including the ORTEP diagram with the thermal ellipsoids drawn at 40% probability level (right). Solvent molecules in the asymmetric unit have been removed for clarity in the representation on the left. The Fe sites in the [**Ring$_{Fe}$**] unit are disordered over multiple positions.

[$NH_2(CH_2C_6H_5)(CH_2CH_2C_6H_5)$][$Cr_7Fe^{II}F_8(O_2C^tBu)_{16}$] (Ph$_{Fe}$): N-benzyl-2-phenylethan-1-amine [**TPh** – H] (0.70 g, 3.30 mmol) was added to 15 g of pivalic acid in an open Teflon flask alongside $CrF_3 \cdot 4\ H_2O$ (3.00 g, 16.57 mmol) and heated to 140 °C under a N_2 atmosphere. After 30 minutes, $FeCl_2 \cdot 4\ H_2O$ (0.66 g, 3.30 mmol) was added to the reaction mixture and stirred at 140 °C for 24 h. The reaction was allowed to cool to RT before addition of acetonitrile (100 mL) precipitating a green solid which was collected by filtration. The product was extracted in toluene (100 mL) and solvent was removed under a reduced pressure. The crude product was purified by flash column chromatography (toluene: EtOAc, 50:1, v/v) to give a green crystalline solid (0.85 g, 11 %). Large, green, single crystals suitable for single crystal XRD were obtained following slow evaporation of toluene. Elemental analysis (%) calcd. for $C_{95}H_{162}Cr_7F_8NFeO_{32}$: C 47.50, H 6.80, N 0.58, Cr 15.15, Fe 2.32; found: C 47.59, H 6.94, N 0.61, Cr 14.35, Fe 2.30. Positive ESI-MS (dissolved in THF, run in MeOH): m/z = 2401.6 [**Ph$_{Fe(III)}$**]$^+$.

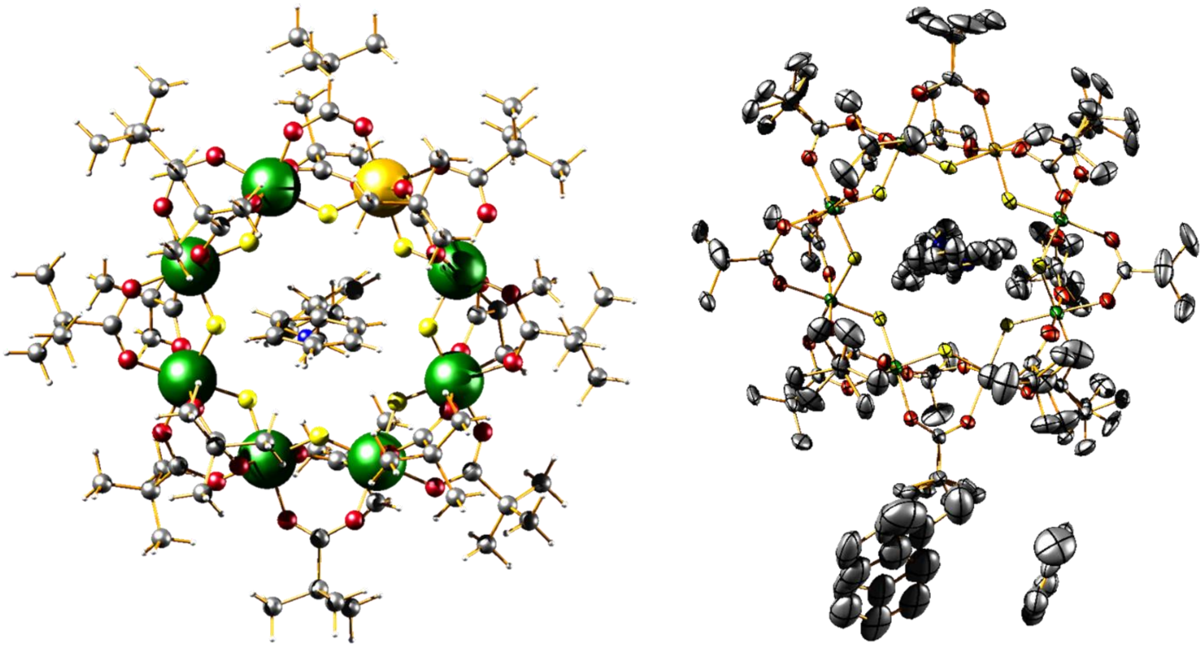

Figure S40: Single crystal X-ray structure of **Ph$_{Co}$** (Co: gold, Cr: dark green, F: yellow, O: red, N: blue, C: grey, H: white) including the ORTEP diagram with the thermal ellipsoids drawn at 40% probability level (right). Solvent molecules in the asymmetric unit have been removed for clarity in the representation on the left. The Co sites in the [**Ring$_{Co}$**] unit are disordered over multiple positions.

[$NH_2(CH_2C_6H_5)(CH_2CH_2C_6H_5)$][$Cr_7Co^{II}F_8(O_2C^tBu)_{16}$] (Ph$_{Co}$): N-benzyl-2-phenylethan-1-amine [**TPh** – H] (0.50 g, 2.37 mmol) was added to 15 g of pivalic acid in a Teflon flask alongside $CrF_3 \cdot 4\ H_2O$ (3.00 g, 16.57 mmol) and $CoCO_3$ (0.47 g, 3.95 mmol) before heating to 160 °C for 24 h under a N_2 atmosphere. The reaction was allowed to cool to RT before addition of acetonitrile (100 mL) precipitating a green solid which was collected by filtration. The product was extracted in toluene (100 mL) and solvent was removed under a reduced pressure. The crude product was purified by flash column chromatography (toluene: EtOAc, 50:1, v/v) to give a green crystalline solid (0.75 g, 13 %). Large, green, single crystals suitable for single crystal XRD were obtained following slow evaporation of toluene. Elemental analysis (%) calcd. for $C_{98}H_{168}Cr_7F_8NCoO_{33}$ (+ 1 acetone): C 47.78, H 6.87, N 0.57, Cr 14.78, Co 2.39; found: C 47.72, H 6.81, N 0.60, Cr 13.98, Co 2.37. Positive ESI-MS (dissolved in THF, run in MeOH): m/z = 2427.6 [**Ph$_{Co}$** + Na]$^+$.

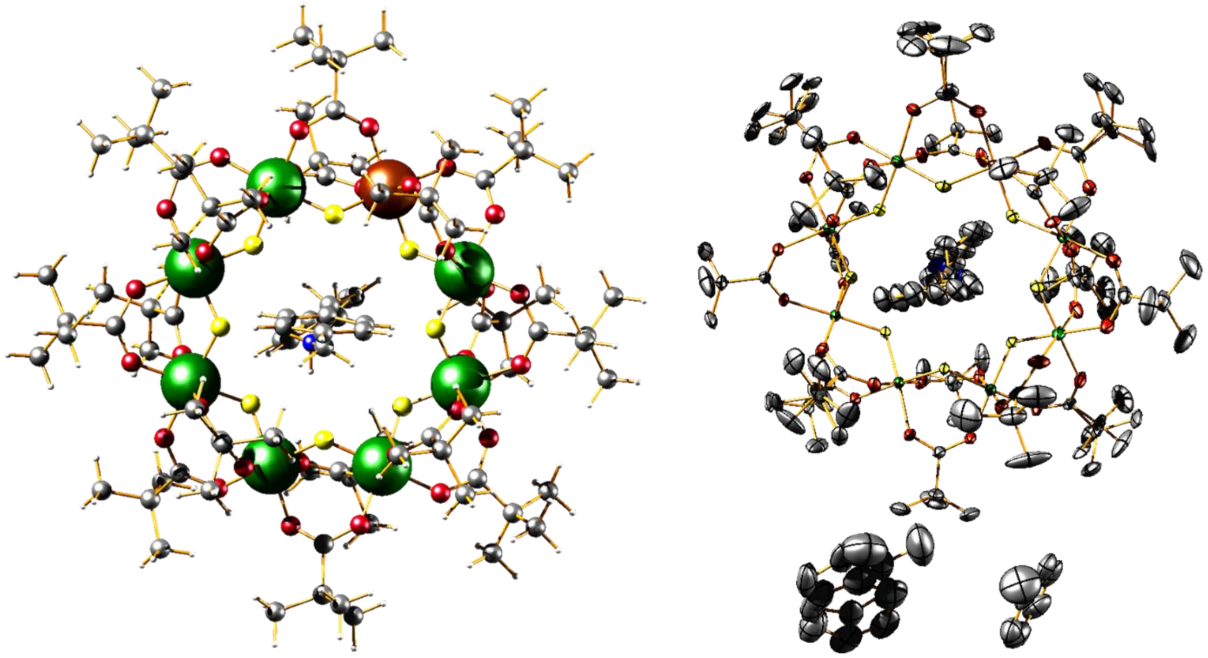

Figure S41: Single crystal X-ray structure of **Ph$_{Ni}$** (Ni: brown, Cr: dark green, F: yellow, O: red, N: blue, C: grey, H: white) including the ORTEP diagram with the thermal ellipsoids drawn at 40% probability level (right). Solvent molecules in the asymmetric unit have been removed for clarity in the representation on the left. The Ni sites in the [**Ring$_{Ni}$**] unit are disordered over multiple positions.

[$NH_2(CH_2C_6H_5)(CH_2CH_2C_6H_5)$][$Cr_7Ni^{II}F_8(O_2C^tBu)_{16}$] (Ph$_{Ni}$): N-benzyl-2-phenylethan-1-amine [**TPh** – H] (0.50 g, 2.37 mmol,) was added to 15 g of pivalic acid in an open Teflon flask alongside $CrF_3 \cdot 4\,H_2O$ (3.00 g, 16.57 mmol) and [$2\,NiCO_3 \cdot 3\,Ni(OH)_2 \cdot 4\,H_2O$] (0.40 g, 0.68 mmol) before heating to 160 °C for 24 h. The reaction was allowed to cool to RT before addition of acetone (100 mL) precipitating a green solid which was collected by filtration. The product was extracted in pentane (50 mL) and diluted in acetone (50 mL) before allowing the solvent to evaporate give a green crystalline solid (4.70 g, 82 %). Large, green, single crystals suitable for single crystal XRD were obtained following slow evaporation of toluene. Elemental analysis (%) calcd. for $C_{95}H_{162}Cr_7F_8NNiO_{32}$: C 47.45, H 6.79, N 0.58, Cr 15.13, Ni 2.44; found: C 47.51, H 6.87, N 0.65, Cr 14.47, Ni 2.38. Positive ESI-MS (dissolved in THF, run in MeOH): m/z = 2427.6 [**Ph$_{Ni}$** + Na]$^+$.

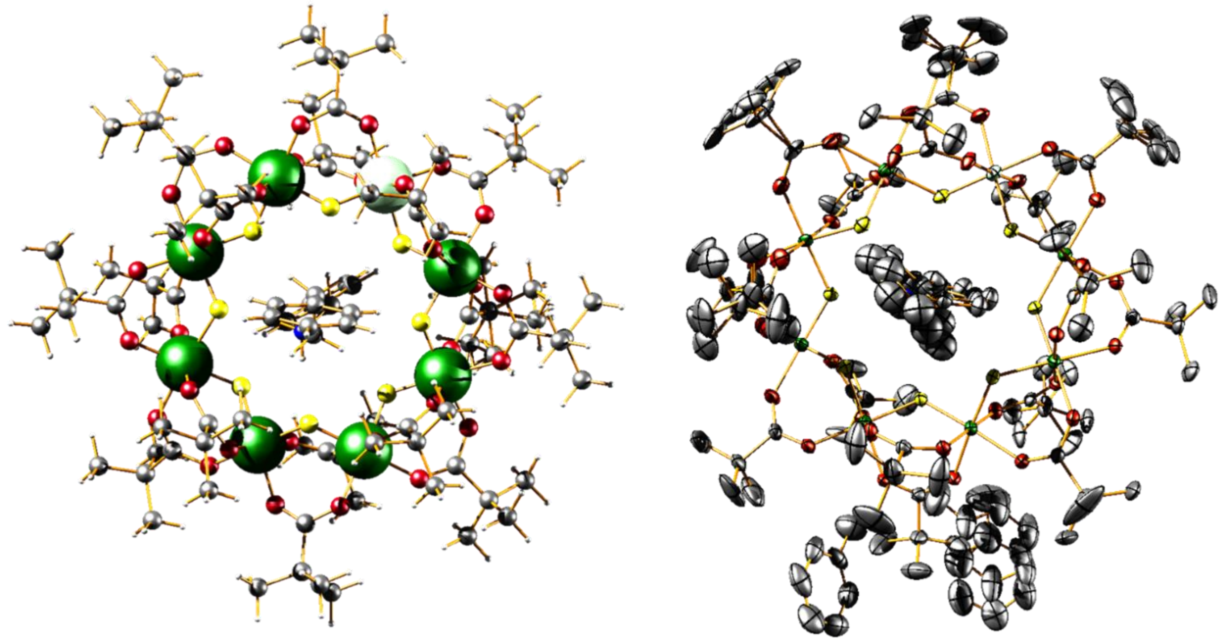

Figure S42: Single crystal X-ray structure of **Ph$_{Cu}$** (Cu: light green, Cr: dark green, F: yellow, O: red, N: blue, C: grey, H: white) including the ORTEP diagram with the thermal ellipsoids drawn at 40% probability level (right). Solvent molecules in the asymmetric unit have been removed for clarity in the representation on the left. The Cu sites in the [**Ring$_{Cu}$**] unit are disordered over multiple positions.

$[NH_2(CH_2C_6H_5)(CH_2CH_2C_6H_5)][Cr_7Cu^{II}F_8(O_2C^tBu)_{16}]$ (Ph$_{Cu}$): N-benzyl-2-phenylethan-1-amine [**TPh** – H] (0.50 g, 2.37 mmol) was added to 15 g of pivalic acid in an open Teflon flask alongside $CrF_3 \cdot 4\ H_2O$ (3.00 g, 16.57 mmol) and $[CuCO_3 \cdot Cu(OH)_2]$ (0.37 g, 1.68 mmol,) before heating to 140 °C for 24 h. The reaction was allowed to cool to RT before addition of acetonitrile (100 mL) precipitating a green solid which was collected by filtration. The product was extracted in toluene (100 mL) and solvent was removed under a reduced pressure. The crude product was purified by flash column chromatography (toluene: EtOAc, 50:1, v/v) to give a green crystalline solid (0.69 g, 12 %). Large, green, single crystals suitable for single crystal XRD were obtained following slow evaporation of toluene. Elemental analysis (%) calcd. for $C_{95}H_{162}Cr_7F_8NCuO_{32}$: C 47.35, H 6.78, N 0.58, Cr 15.10, Cu 2.64; found: C 47.52, H 6.77, N 0.58, Cr 14.56, Cu 2.15. Positive ESI-MS (dissolved in THF, run in MeOH): m/z = 2432.6 [**Ph$_{Cu}$** + Na]$^+$.

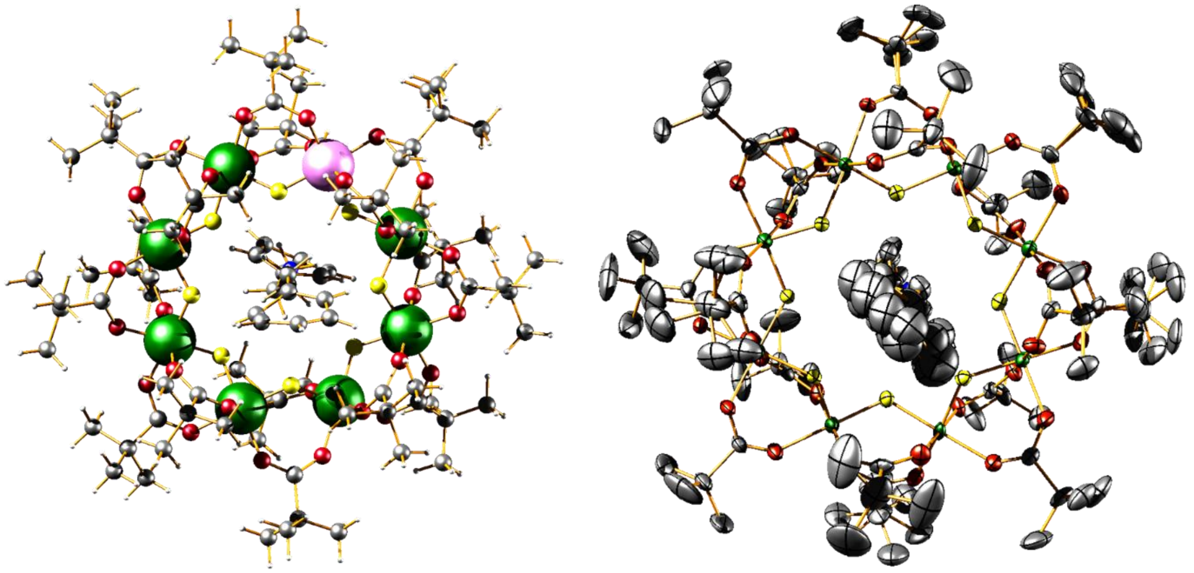

Figure S43: Single crystal X-ray structure of **Ph_{Zn}** (Zn: pink, Cr: dark green, F: yellow, O: red, N: blue, C: grey, H: white) including the ORTEP diagram with the thermal ellipsoids drawn at 40% probability level (right). Solvent molecules in the asymmetric unit have been removed for clarity in the representation on the left. The Zn sites in the [**$Ring_{Zn}$**] unit are disordered over multiple positions.

$[NH_2(CH_2C_6H_5)(CH_2CH_2C_6H_5)][Cr_7Zn^{II}F_8(O_2C^tBu)_{16}]$ (Ph_{Zn}): N-benzyl-2-phenylethan-1-amine [**TPh** – H] (0.75 g, 3.56 mmol) was added to 15 g of pivalic acid in an open Teflon flask alongside $CrF_3 \cdot 4\,H_2O$ (3.00 g, 16.57 mmol) and $[ZnCO_3]_2 \cdot [Zn(OH)_2]_3$ (0.25 g, 0.46 mmol) before heating to 140 °C for 24 h. The reaction was allowed to cool to RT before addition of acetonitrile (100 mL) precipitating a green solid which was collected by filtration. The product was extracted in toluene (100 mL) and solvent was removed under a reduced pressure. The crude product was purified by flash column chromatography (toluene: EtOAc, 50:1, v/v) to give a green crystalline solid (1.84 g, 32 %). Large, green, single crystals suitable for single crystal XRD were obtained following slow evaporation of acetone in acetonitrile. Elemental analysis (%) calcd. for $C_{102}H_{170}Cr_7F_8NZnO_{32}$ (+ 1 toluene): C 48.93, H 6.84, N 0.56, Cr 14.54, Zn 2.61; found: C 49.16, H 6.96, N 0.64, Cr 14.20, Zn 1.99. Positive ESI-MS (dissolved in THF, run in MeOH): m/z = 2433.6 [**Ph_{Zn}** + Na]$^+$.

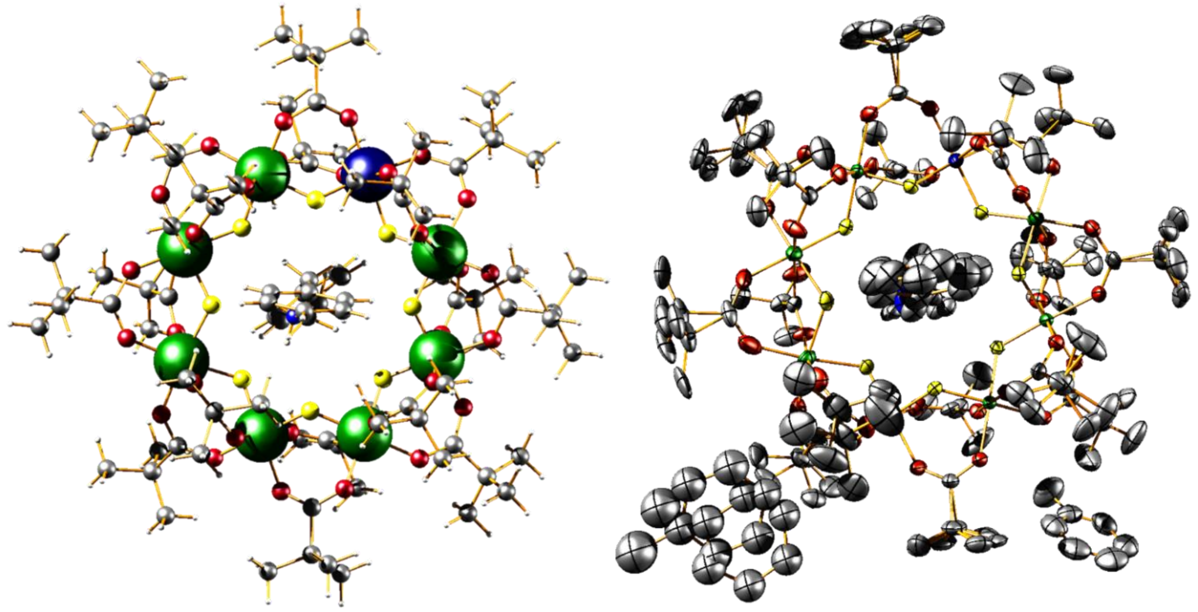

Figure S44: Single crystal X-ray structure of **Ph$_{Cd}$**(Cd: dark blue, Cr: dark green, F: yellow, O: red, N: blue, C: grey, H: white) including the ORTEP diagram with the thermal ellipsoids drawn at 40% probability level (right). Solvent molecules in the asymmetric unit have been removed for clarity in the representation on the left. The Cd sites in the [**Ring$_{Cd}$**] unit are disordered over multiple positions.

[$NH_2(CH_2C_6H_5)(CH_2CH_2C_6H_5)$][$Cr_7Cd^{II}F_8(O_2C^tBu)_{16}$] (Ph$_{Cd}$): N-benzyl-2-phenylethan-1-amine [**TPh** – H] (0.75 g, 3.36 mmol) was added to 15 g of pivalic acid in an open Teflon flask alongside $CrF_3 \cdot 4\ H_2O$ (3.00 g, 16.57 mmol) and heated to 140 °C under a N_2 atmosphere. After 30 minutes, $CdCO_3$ (0.61 g, 3.56 mmol) was added to the reaction mixture and stirred at 140 °C for 24 h. The reaction was allowed to cool to RT before addition of acetonitrile (100 mL) precipitating a green solid which was collected by filtration. The product was extracted in toluene (100 mL) and solvent was removed under a reduced pressure. The crude product was purified by flash column chromatography (toluene: EtOAc, 50:1, v/v) to give a green crystalline solid (0.38 g, 7 %). Large, green, single crystals suitable for single crystal XRD were obtained following slow evaporation of toluene. Elemental analysis (%) calcd. for $C_{102}H_{170}Cr_7F_8NCdO_{32}$ (+ 1 toluene): C 48.03, H 6.72, N 0.55, Cr 14.27, Cd 4.41; found: C 48.06, H 6.80, N 0.61, Cr 13.52, Cd 4.09. Positive ESI-MS (dissolved in THF, run in MeOH): m/z = 2481.6 [**Ph$_{Cd}$** + Na]$^+$.

Table S7: Crystal data and structure refinement for **Ph$_M$** (M = Mn^{II}, Fe^{II}, Co^{II}, Ni^{II}, Cu^{II}, Zn^{II}, Cd^{II}).

Identification code<br>Deposition number	Ph_Mn<br>2176290	Ph_Fe<br>2176293	Ph_Co<br>217691	Ph_Ni<br>2176288	Ph_Cu<br>2176292	Ph_Zn<br>2176289	Ph_Cd<br>2176287
Empirical formula	$Cr_7MnF_8C_{109}H_{178}O_{32}N$	$Cr_7FeF_8C_{109}H_{178}O_{32}N$	$Cr_7CoF_8C_{109}H_{178}O_{32}N$	$Cr_7NiF_8C_{109}H_{178}O_{32}N$	$Cr_7CuF_8C_{109}H_{178}O_{32}N$	$Cr_7ZnF_8C_{99}H_{168}O_{32}N_3$	$C_{109}H_{178}Cd_{0.98}Cr_{6.95}F_8NO_{32}$
Formula weight	2585.45	2586.36	2589.44	2589.22	2594.05	2411.62	2638.55
Temperature/K	100.00(10)	100.00(10)	100.00(10)	100.01(10)	100.01(10)	150.01(11)	100.00(10)
Crystal system	orthorhombic	orthorhombic	orthorhombic	orthorhombic	orthorhombic	orthorhombic	orthorhombic
Space group	Pbca	Pbca	Pbca	Pbca	Pbca	Pbca	Pbca
a/Å	30.6258(7)	30.5511(6)	30.4915(7)	30.5564(7)	30.5956(6)	30.7976(6)	30.6078(5)
b/Å	27.0256(6)	27.0322(6)	27.0369(5)	27.0973(13)	27.1139(6)	27.0967(8)	27.0995(9)
c/Å	32.2049(11)	32.1831(8)	32.3395(5)	32.3136(9)	32.2824(9)	32.5142(7)	32.2232(7)
α/°	90	90	90	90	90	90	90
β/°	90	90	90	90	90	90	90
γ/°	90	90	90	90	90	90	90
Volume/Å^3	26655.4(12)	26578.8(10)	26660.5(9)	26755.5(16)	26780.4(11)	27133.5(11)	26727.7(11)
Z	8	8	8	8	8	8	8
ρ_{calc}g/cm^3	1.289	1.293	1.29	1.286	1.287	1.181	1.311
μ/mm^{-1}	0.718	0.734	6.13	0.761	0.779	0.784	0.774
F(000)	10880	10888	10896	10904	10912	10120	11048
Crystal size/mm^3	0.341 × 0.284 × 0.191	0.346 × 0.221 × 0.171	0.59 × 0.138 × 0.109	0.527 × 0.434 × 0.259	0.527 × 0.434 × 0.259	0.842 × 0.718 × 0.427	0.408 × 0.284 × 0.21
Radiation	Mo Kα (λ = 0.71073)	Mo Kα (λ = 0.71073)	Cu Kα (λ = 1.54184)	Mo Kα (λ = 0.71073)	Mo Kα (λ = 0.71073)	Mo Kα (λ = 0.71073)	Mo Kα (λ = 0.71073)
2Θ range for data collection/°	4.846 to 52.744	4.734 to 52.742	5.152 to 130.174	4.866 to 52.744	5.014 to 52.746	4.258 to 52.744	4.744 to 52.744
Index ranges	-38 ≤ h ≤ 23, -19 ≤ k ≤ 33, -24 ≤ l ≤ 40	-38 ≤ h ≤ 35, -33 ≤ k ≤ 20, -18 ≤ l ≤ 40	-35 ≤ h ≤ 34, -31 ≤ k ≤ 28, -38 ≤ l ≤ 36	-38 ≤ h ≤ 20, -33 ≤ k ≤ 28, -30 ≤ l ≤ 40	-20 ≤ h ≤ 38, -31 ≤ k ≤ 33, -21 ≤ l ≤ 40	-34 ≤ h ≤ 38, -33 ≤ k ≤ 12, -40 ≤ l ≤ 40	-22 ≤ h ≤ 38, -22 ≤ k ≤ 33, -40 ≤ l ≤ 24
Reflections collected	72961	70731	90634	64457	68387	104538	73573
Independent reflections	27231 [R_{int} = 0.0615, R_{sigma} = 0.1055]	27146 [R_{int} = 0.0811, R_{sigma} = 0.1356]	22705 [Rint = 0.0906, Rsigma = 0.0901]	27202 [R_{int} = 0.0729, R_{sigma} = 0.1225]	27360 [R_{int} = 0.0534, R_{sigma} = 0.0785]	27719 [Rint = 0.0881, Rsigma = 0.1033]	27287 [Rint = 0.0520, Rsigma = 0.0820]
Data/ restraints/ parameters	27231/3131/1720	27146/4696/1794	22705/5180/1868	27202/4765/1794	27360/4399/1757	27719/5245/1726	27287/5620/1819
Goodness-of-fit on F^2	1.025	1.023	1.051	1.022	1.023	1.038	1.012
Final R indexes [I>=2σ (I)]	R_1 = 0.0740, wR_2 = 0.1663	R_1 = 0.0702, wR_2 = 0.1521	R1 = 0.0696, wR2 = 0.1911	R_1 = 0.0675, wR_2 = 0.1436	R_1 = 0.0661, wR_2 = 0.1585	R1 = 0.0653, wR2 = 0.1429	R1 = 0.0784, wR2 = 0.1732
Final R indexes [all data]	R_1 = 0.1370, wR_2 = 0.2006	R_1 = 0.1448, wR_2 = 0.1933	R1 = 0.1018, wR2 = 0.2123	R_1 = 0.1369, wR_2 = 0.1766	R_1 = 0.1067, wR_2 = 0.1847	R1 = 0.1214, wR2 = 0.1717	R1 = 0.1311, wR2 = 0.2059
Largest diff. peak/hole / e Å^{-3}	0.64/-0.65	0.57/-0.50	0.56/-0.61	0.63/-0.56	0.94/-0.58	0.69/-0.52	0.72/-0.97

References

(1) Larsen, F. K.; Overgaard, J.; Christensen, M.; Mcintyre, G. J.; Timco, G.; Winpenny, R. E. P. Metal Distribution and Disorder in the Crystal Structure of $[NH_2Et_2][Cr_7MF_8(^tBuCO_2)_{16}]$ Wheel Molecules for M = Mn, Fe, Co, Ni, Cu, Zn and Cd. *Acta Crystallogr.* **2014**, *B70* (6), 932–941. https://doi.org/10.1107/S2052520614019179.

(2) Ballesteros, B.; Faust, T. B.; Lee, C. F.; Leigh, D. A.; Muryn, C. A.; Pritchard, R. G.; Schultz, D.; Teat, S. J.; Timco, G. A.; Winpenny, R. E. P. Synthesis, Structure, and Dynamic Properties of Hybrid Organic-Inorganic Rotaxanes. *J. Am. Chem. Soc.* **2010**, *132* (43), 15435–15444. https://doi.org/10.1021/ja1074773.

(3) Shrivastav, V.; Nahin, M.; Hogan, C. J.; Larriba-Andaluz, C. Benchmark Comparison for a Multi-Processing Ion Mobility Calculator in the Free Molecular Regime. *J. Am. Soc. Mass Spectrom.* **2017**, *28* (8), 1540–1551. https://doi.org/10.1007/s13361-017-1661-8.

(4) McInnes, E. J. L.; Timco, G. A.; Whitehead, G. F. S.; Winpenny, R. E. P. Heterometallic Rings: Their Physics and Use as Supramolecular Building Blocks. *Angew. Chem., Int. Ed.* **2015**, *54* (48), 14244–14269. https://doi.org/10.1002/anie.201502730.

(5) Morsa, D.; Gabelica, V.; De Pauw, E. Effective Temperature of Ions in Traveling Wave Ion Mobility Spectrometry. *Anal. Chem.* **2011**, *83* (14), 5775–5782. https://doi.org/10.1021/ac201509p.

(6) Merenbloom, S. I.; Flick, T. G.; Williams, E. R. How Hot Are Your Ions in TWAVE Ion Mobility Spectrometry? *J. Am. Soc. Mass Spectrom.* **2012**, *23*, 553–562. https://doi.org/10.1007/s13361-011-0313-7.

(7) Jurneczko, E.; Barran, P. E. How Useful Is Ion Mobility Mass Spectrometry for Structural Biology? The Relationship between Protein Crystal Structures and Their Collision Cross Sections in the Gas Phase. *Analyst* **2011**, *136* (1), 20–28. https://doi.org/10.1039/c0an00373e.

(8) Prell, J. S. *Modelling Collisional Cross Sections*, 1st ed.; Elsevier B.V., **2019**; Vol. 83. https://doi.org/10.1016/bs.coac.2018.08.001.

(9) Brocker, E. R.; Anderson, S. E.; Northrop, B. H.; Stang, P. J.; Bowers, M. T. Structures of Metallosupramolecular Coordination Assemblies Can Be Obtained by Ion Mobility Spectrometry-Mass Spectrometry. *J. Am. Chem. Soc.* **2010**, *132* (38), 13486–13494. https://doi.org/10.1021/ja105702y.

(10) Chan, Y. T.; Li, X.; Yu, J.; Carri, G. A.; Moorefield, C. N.; Newkome, G. R.; Wesdemiotis, C. Design, Synthesis, and Traveling Wave Ion Mobility Mass Spectrometry Characterization of Iron(II)- and Ruthenium(II)-Terpyridine Metallomacrocycles. *J. Am. Chem. Soc.* **2011**, *133* (31), 11967–11976. https://doi.org/10.1021/ja107307u.

(11) Wright, V. E.; Castro-Gómez, F.; Jurneczko, E.; Reynolds, J. C.; Poulton, A.; Christie, S. D. R.; Barran, P.; Bo, C.; Creaser, C. S. Structural Studies of Metal Ligand Complexes by Ion Mobility-Mass Spectrometry. *Int. J. Ion Mobil. Spectrom.* **2013**, *16* (1), 61–67. https://doi.org/10.1007/s12127-013-0122-8.

(12) Surman, A. J.; Robbins, P. J.; Ujma, J.; Zheng, Q.; Barran, P. E.; Cronin, L. Sizing and Discovery of Nanosized Polyoxometalate Clusters by Mass Spectrometry. *J. Am. Chem. Soc.* **2016**, *138* (11), 3824–3830. https://doi.org/10.1021/jacs.6b00070.

(13) Ebbert, K. E.; Schneider, L.; Platzek, A.; Drechsler, C.; Chen, B.; Rudolf, R.; Clever, G. H. Resolution of Minor Size Differences in a Family of Heteroleptic Coordination Cages by Trapped Ion Mobility ESI-MS. *Dalton Trans.* **2019**, *48* (29), 11070–11075. https://doi.org/10.1039/c9dt01814j.

(14) Cross, J. T. D.; Stimson, V. R. The Gas-Phase Hydrogen Bromide-Catalysed Decomposition of Trimethylacetic Acid. *Chem. Commun. London* **1966**, No. 12, 350–351. https://doi.org/10.1039/C19660000350.

(15) Lassahn, P. G.; Lozan, V.; Timco, G. A.; Christian, P.; Janiak, C.; Winpenny, R. E. P. Homo- and Heterometallic Carboxylate Cage Complexes as Precatalysts for Olefin Polymerization - Activity Enhancement through "Inert Metals." *J. Catal.* **2004**, *222* (1), 260–267. https://doi.org/10.1016/j.jcat.2003.10.028.

(16) Steiner, A.; Stalke, D. Unexpected Coordination of Aminoiminophosphoranate Ligands with Alkali Metals. *Inorg. Chem.* **1993**, *32* (10), 1977–1981.

(17) Kamalesh Babu, R. P.; Aparna, K.; McDonald, R.; Cavell, R. G. Dimeric Bridged and Chelated Alkali Metal (Li and Na) Bis(Iminophosphorano)Methanide Complexes with Contrasting Structures. *Inorg. Chem.* **2000**, *39* (21), 4981–4984. https://doi.org/10.1021/ic991364z.

(18) Benmansour, S.; Delgado, E.; Gómez-García, C. J.; Hernández, D.; Hernández, E.; Martin, A.; Perles, J.; Zamora, F. Coordination Polymers Based on Diiron Tetrakis(Dithiolato) Bridged by Alkali Metals, Electrical Bistability around Room Temperature, and Strong Antiferromagnetic Coupling. *Inorg. Chem.* **2015**, *54* (5), 2243–2252. https://doi.org/10.1021/ic502789v.

(19) Geue, N.; Bennett, T. S.; Ramakers, L. A. I.; Timco, G. A.; Armentrout, P. B.; McInnes, E. J. L.; Burton, N. A.; Winpenny, R. E. P.; Barran, P. E. Adduct Ions as Diagnostic Probes for Metallosupramolecular Complexes Using Ion Mobility Mass Spectrometry. *ChemRxiv* **2022**. https://doi.org/10.26434/chemrxiv-2022-lnq8t.

(20) Ducommun, Y.; Newman, K. E.; Merbach, A. E. High-Pressure ^{17}O NMR Evidence for a Gradual Mechanistic Changeover from Ia to Id for Water Exchange on Divalent Octahedral Metal Ions Going from Manganese(II) to Nickel(II). *Inorg. Chem* **1980**, *19*, 3696–3703.

(21) Baes, C. F.; Mesmer, R. E. Hydrolysis of Cations; Krieger Publishing Company: Malabar, FL, USA, 1976.

(22) Powell, D. H.; Helm, L.; Merbach, A. E. ^{17}O Nuclear Magnetic Resonance in Aqueous Solutions of Cu^{2+}: The Combined Effect of Jahn-Teller Inversion and Solvent Exchange on Relaxation Rates. *J. Chem. Phys.* **1991**, *95* (12), 9258–9265. https://doi.org/10.1063/1.461206.

(23) Inada, Y.; Mohammed, A. M.; Loeffler, H. H.; Funahashi, S. Water-Exchange Mechanism for Zinc(II), Cadmium(II), and Mercury(II)-Ions in Water as Studied by Umbrella-

Sampling Molecular-Dynamics Simulations. *Helv. Chim. Acta* **2005**, *88* (3), 461–469. https://doi.org/10.1002/hlca.200590030.

(24) Martell, A. E.; Smith, R. M. Critical Stability Constants; Plenum Press: New York City, NY, USA, **1989**.

(25) Lincoln, S. F. Mechanistic Studies of Metal Aqua Ions: A Semi-Historical Perspective. *Helv. Chim. Acta* **2005**, *88* (3), 523–545. https://doi.org/10.1002/hlca.200590036.

(26) Richens, D. T. Ligand Substitution Reactions at Inorganic Centers. *Chem. Rev.* **2005**, *105* (6), 1961–2002. https://doi.org/10.1021/cr030705u.

(27) Bondi, A. Van Der Waals Volumes and Radii. *J. Phys. Chem.* **1964**, *68* (3), 441–451.
